# Occurrence, associated factors, and outcomes of delirium in patients in an adult acute general medicine service in England: a 10-year longitudinal, observational study

**DOI:** 10.1016/j.lanhl.2025.100731

**Published:** 2025-07

**Authors:** Jasmine Ming Gan, Emily Louise Boucher, Nicola Georgia Lovett, Sophie Roche, Sarah Catherine Smith, Sarah Tamsin Pendlebury

**Affiliations:** a Wolfson Centre for Prevention of Stroke and Dementia, Nuffield Department of Clinical Neurosciences, John Radcliffe Hospital, University of Oxford, Oxford, UK; b Departments of Acute General Internal Medicine and Geratology, Oxford University Hospitals NHS Foundation Trust, Oxford, UK; c Medical Sciences Division, University of Oxford, Oxford, UK; d NIHR Oxford Biomedical Research Centre, Oxford University Hospitals NHS Foundation Trust, Oxford, UK

## Abstract

**Background:**

Reliable estimates of delirium occurrence and outcomes are necessary to inform hospital services, research, and policy, but inclusive cohorts with long-term follow-up are scarce. We aimed to assess the age-specific occurrence of delirium in acute general (internal) medicine, associated factors, and 10-year outcomes stratified by age and comorbid dementia status.

**Methods:**

This longitudinal, observational study was done at the John Radcliffe Hospital (Oxford, UK). We included consecutive adult patients aged 16 years and older in an acute general (internal) medicine service over six 8-week periods (between Sept 4, 2010, and Nov 15, 2018). Delirium was diagnosed prospectively using the Confusion Assessment Method and Diagnostic and Statistical Manual of Mental Disorders, fourth edition, criteria and subcategorised as prevalent (≤48 h of admission) or incident (>48 h postadmission). Odds ratios adjusted (_adj_ORs) for demographics, comorbidity, frailty, and illness severity were calculated for binarised outcomes and adjusted hazard ratios (_adj_HRs) were calculated for time to death.

**Findings:**

1846 patients were admitted to acute general (internal) medicine (mean age 68·2 years [SD 20·0], age range 16–102 years), 426 (23% [95% CI 21–25]) of whom had delirium (prevalent n=290 [68%], incident n=73 [17%], both prevalent and incident n=63 [15%]), of whom 134 (31·5%) had dementia. 950 (51·5%) patients were female, 895 (48·5%) were male, and sex data were missing for one patient. Delirium increased with age, from six (2% [95% CI 1–4]) of 340 patients younger than 50 years and 31 (9% [6–13]) of 333 patients at age 50–64 years to 57 (20% [16–25]) of 281 at age 65–74 years, 245 (35% [31–38]) of 704 at age 75–89 years, and 87 (46% [39–54]) of 188 at age 90 years and older. Of the 37 patients younger than 65 years who had delirium, 28 (76%) had an underlying neurological or neuropsychiatric disorder. In those aged 65 years or older, delirium was overall associated (all p<0·001, age and sex adjusted) with dementia (_adj_OR 3·63 [95% CI 2·65–4·98]), pre-admission dependency (2·63 [2·02–3·43]), comorbidity burden (1·04 [1·02–1·05]), and frailty (moderate *vs* low risk 3·62 [2·70–4·85] and high *vs* low risk 11·85 [7·24–19·42]), with stronger associations in patients without comorbid dementia than in those with comorbid dementia. Delirium predicted inpatient stay longer than 7 days (_adj_OR 2·48 [1·84–3·35]), discharge care needs (2·41 [1·70–3·40]), and mortality during admission (2·45 [1·52–3·94]). The increased risk of death in the delirium group was highest in the immediate postadmission period and attenuated thereafter, but was maintained for up to 10 years of follow-up (_adj_HR 2·03 [95% CI 1·40–2·97] for 30-day mortality *vs* 1·52 [1·30–1·77] for 10-year mortality). Excess inpatient mortality was highest in younger age groups versus older age groups (_adj_OR 4·38 [95% CI 1·18–16·31]; p=0·028 at age 65–74 years *vs* 1·96 [1·02–3·75]; p=0·043 at age 75–89 years and 2·86 [1·14–7·16]; p=0·025 at age 90 years or older) and in those without versus with comorbid dementia (_adj_OR 3·02 [1·73–5·25]; p<0·001 *vs* 1·47 [0·58–3·75]; p=0·42).

**Interpretation:**

Our findings support current guidelines for routine on-admission delirium screening from age 65 years. Delirium outcomes are relatively more adverse in those aged 65–74 years without comorbid dementia in whom interventions and clinical trials should be prioritised.

**Funding:**

National Institute for Health and Care Research and the Medical Research Council.

## Introduction

Delirium is characterised by an acute and fluctuating disturbance in attention, awareness, and cognition. It is common in the acute hospital population, the majority of whom are admitted to general medicine services.^1^ The social and economic burdens of delirium are considerable, with hospital costs estimated at US$800–24 000 per admission and expected to rise with population ageing.^2^

**Research in context**

**Evidence before this study**

We reviewed the evidence on delirium occurrence, factors associated with delirium, and outcomes of delirium in patients admitted to hospital in acute general (internal) medicine services, the largest acute hospital specialty. In a 2020 systematic review and meta-analysis of medical services, pooled delirium occurrence was 23% (95% CI 19–26%, *I*^*2*^=94%, n=33 studies, mean age 80 years) but 30 of the included studies had restrictive eligibility criteria, which might have reduced observed estimates, and only three studies of acute general medicine included adults of any age, of which only one reported age-specific delirium occurrence across the full adult age range. In a 2023 systematic review of delirium associated factors (n=315 studies), more than three-quarters of the 38 acute general (internal) or geriatric medicine studies had selection criteria potentially affecting observed relationships. In a 2020 systematic review of delirium outcomes, the pooled odds ratio for death up to 3 years was 3·64 (95% CI 2·99–4·44, n=29 studies), but no study adjusted for all three important confounders of comorbidity burden, illness severity, and frailty, or followed up patients beyond 3 years. Very few studies stratified findings by comorbid dementia status or other important clinical factors. We did an updated literature search of MEDLINE, from Jan 1, 2017, to Feb 28, 2025, using search terms including “delirium”, “risk factors”, and “outcomes” with no language restrictions and found no studies addressing all the limitations we identified.

**Added value of this study**

We filled the identified evidence gaps by studying an inclusive consecutive cohort of nearly 2000 well characterised patients in the acute general medical setting with follow-up to 10 years. Overall, a quarter of participants had delirium; however, delirium was very uncommon in participants younger than 50 years and affected only 9% of those aged 50–64 years, increasing markedly thereafter to affect 20% of those aged 65–74 years, 35% of those aged 75–89 years, and 46% of those aged 90 years or older. Most delirium, especially in younger patients, occurred in the absence of a comorbid dementia diagnosis and prevalent (on-admission) delirium was much more common than incident delirium (arising during admission). Patients younger than 65 years with delirium often had underlying brain conditions, alcohol misuse, or severe illness. Delirium in older patients was associated with both vulnerability and precipitating factors, in keeping with previous reports. However, history of depression, Charlson Comorbidity Index, stroke, hyponatraemia, and constipation were only associated with delirium in those without comorbid dementia, after adjustment for age and sex. Our study showed that delirium is an independent predictor of poor outcomes over and above comorbidity, illness severity, and frailty. The increased risk of death was front-loaded but persisted in the longer term to 10 years of follow-up. The excess mortality with delirium was greater in younger patients without dementia residing at home.

**Implications of all the available evidence**

Our findings support current UK guidance (NHS Getting It Right First Time) for routine on-admission delirium screening in patients aged 65 years and older who are admitted to hospital, with targeted screening in younger patients who are at risk. Most delirium occurs in the absence of a dementia diagnosis and delirium screening is therefore necessary to identify patients with cognitive frailty in whom physical frailty, multimorbidity, and high care needs are common. Delirium is an independent predictor of adverse outcomes, particularly in younger, more robust patients without comorbid dementia in whom delirium interventions and future clinical trials should be prioritised.

Reliable estimates of delirium occurrence are important for service planning, resource allocation, and policy. A systematic review (of 33 studies) reported delirium occurrence of 23% in medical inpatients,^3^^,^^4^ but heterogeneity was substantial and many studies excluded patients with characteristics associated with delirium (eg, unable to consent, communication difficulties, and pre-existing dementia^4^), and dementia prevalence varied substantially from 8% to 100%.^3^ Furthermore, data on younger patients (<65 years) were scarce (appendix pp 4–10).

Existing studies on factors associated with delirium are also largely derived from selective cohorts. Only around 10% of 315 studies in a recent systematic review^5^ were from acute general (internal) or geriatric medicine settings encompassing a broad range of medical conditions, and only two were conducted without selection criteria (appendix pp 11–16). Studies on delirium outcomes, including increased length of hospital stay, risk of institutionalisation, and mortality^4^^,^^6^^,^^7^ also have limitations. In a meta-analysis of 71 studies on mortality,^6^ less than half included medical inpatients, long-term follow-up beyond 5 years was rare, and none adjusted for all three key confounders of comorbidity burden, illness severity, and frailty (appendix pp 17–24). The extent to which delirium is an independent predictor of adverse outcomes therefore remains uncertain.

Studies from representative cohorts of adults of all ages are therefore required. We previously assessed delirium occurrence, associated factors, and outcomes in a consecutive cohort of patients in adult acute general medicine.^8^ However, this earlier study was relatively small (n=503), resulting in uncertainty in delirium occurrence in younger patients, and follow-up was only for 2 years. Furthermore, analyses were not stratified by comorbid dementia or frailty. In this Article, we report our findings from a cohort of nearly 2000 patients with over 10 years of follow-up. We aimed to assess age-specific delirium occurrence (both on admission and arising during admission) in patients aged 16 years and older, the factors associated with delirium, and whether delirium predicted adverse outcomes over and above comorbidity burden, illness severity, and frailty. We stratified analyses by age, comorbid dementia, frailty, and residence status where appropriate.

## Methods

### Study design and population

This longitudinal, observational study was done at the John Radcliffe Hospital (Oxford, UK), which provides acute medicine services to the Oxfordshire region (population approximately 800 000) and has no separate geriatric admissions service (appendix p 29). We included all consecutive adult patients aged 16 years or older in acute medical settings admitted under the care of two senior Consultant Physicians (equivalent to Attending Physician in the USA) accredited in both acute general medicine and geriatrics (STP and SCS) over six 8-week periods (cohort 1: Sept 4–Oct 27, 2010; cohort 2: May 13–July 15, 2012; cohort 3: Oct 25–Dec 17, 2015; cohort 4: June 5–July 28, 2016; cohort 5: Nov 19, 2017–Jan 11, 2018; cohort 6: Sept 23–Nov 15, 2018). No exclusion criteria were applied. Data on race or ethnicity were not collected as part of this study but approximately 86% of the background Oxfordshire population is White. Data on sex were collected from medical records.

The study was conducted as audit with subsequent approval for research use as part of the Oxford and Reading Cognitive Comorbidity, Frailty, and Ageing Research Database-Electronic Patient Records (ORCHARD-EPR; South Central Oxford Research Ethics Committee reference 23/SC/0258), and consent was not required. Some data on cohort 1 and cohort 2 have been reported previously.^8^

### Delirium ascertainment

As part of the admission clerking (history and physical examination), all patients aged 65 years and older, and patients younger than 65 years with altered behaviour or who were at risk for delirium (eg, history of stroke, Parkinson’s disease, or alcohol excess), received a cognitive test and screening for delirium using the Confusion Assessment Method (appendix p 30).^8^ The cognitive test was the Mini-Mental State Examination (MMSE) for cohort 1 and, for subsequent cohorts, the Abbreviated Mental Test Score (AMTS).^8^ Low cognitive score was defined as MMSE less than 24 or AMTS less than 9.^9^ All patients were reviewed by STP or SCS as part of standard care on the immediate postadmission ward round and at least every 48 h thereafter. Delirium diagnosis was based on the Diagnostic and Statistical Manual of Mental Disorders, fourth edition, criteria and was made by STP or SCS after discussion with the wider multidisciplinary team where appropriate.

Delirium was categorised as: prevalent if present on admission or within 48 h; incident if developing more than 48 h after admission; or both prevalent and incident where prevalent and incident delirium occurred in the same patient (in patients with prevalent delirium, incident delirium was considered to have occurred if the patient was delirium free for 48 h before a new delirium episode developed). Any delirium was used for overall analyses if either prevalent or incident delirium was present during admission.^8^

### Clinical covariates and outcomes

De-identified data were extracted (paper medical notes, 2010–18; electronic patient records, Cerner Millennium, 2015 onwards) and transcribed onto an electronic data collection form with prespecified data fields based on pre-existing literature.^8^^,^^10^^,^^11^ Prospectively acquired data were supplemented by administrative diagnostic coding (ICD-10 codes) applied by the hospital coding team after discharge according to usual practice. Pre-existing dementia diagnosis was obtained from the patient clerking and medical records.

For patients aged 65 years or older, previous dependency was defined as pre-admission residence at home with a care package, or in a care home. Comorbidity burden was calculated using the updated Charlson Comorbidity Index (CCI)^12^^,^^13^ based on the ICD-10 secondary diagnosis codes. Illness severity measures were obtained from vital observations on admission to the emergency department or medical assessment unit and initial blood test results. Patients with more than one of the systemic inflammatory response syndrome (SIRS) criteria (white cell count, blood pressure, respiratory rate, and temperature)^14^ were considered SIRS positive, indicating severe illness. Frailty was measured using a modified version of the Hospital Frailty Risk Score (HFRS), excluding delirium because delirium was the exposure of interest, and categorised as low (<5 points), intermediate (5–15 points), or high (>15 points) risk.^15^ Patients in cohorts 1 and 2 were assessed as being at risk of pressure sores if they had a pressure sore prediction score of 6 or more. Patients in cohorts 3 to 6 were assessed as being at risk of pressure sores if they had a Braden score of less than 19 (nursing pressure sore risk assessment tool changed over the study period).

Factors that occurred during admission including urinary incontinence, faecal incontinence, being bedbound, sleep disturbance, inpatient falls, and new urinary catheterisation were obtained from hand-searching of medical notes, including documentation by doctors, nurses, and allied health professionals during routine clinical review. Constipation was defined as new prescription of laxatives, suppository, or enema. For patients younger than 65 years, clinical covariates were not collected routinely but hand-searching of records was undertaken in those with delirium to establish the associated factors for descriptive purposes.

Mortality data were available for all patients via the hospital information analysts according to the Office of National Statistics, with data censored on April 20, 2023. For patients aged 65 years or older, length of hospital stay was defined as the time from the day of admission to the day of acute hospital discharge. Increased care needs at discharge were defined as a new placement in a care home, new or increased care package at home, discharge to community hospital, or intermediate care.

### Statistical analysis

The occurrence of any delirium by age was the primary analysis. The proportions of patients with any delirium and dementia with 95% CIs were calculated across age groups. For incident and prevalent delirium, the estimated percentages with 95% CIs were calculated for the cohort as a whole without age stratification. Baseline characteristics between the delirium and no delirium groups were compared using *t* test, Mann–Whitney *U* test, and χ^2^ test, as appropriate. Odds ratios (ORs) were calculated for factors associated with delirium for the cohort overall for any, prevalent, and incident delirium, and then after stratification by the presence or absence of comorbid dementia. Unadjusted ORs were calculated and then adjusted for age and sex followed by multivariable analysis entering all factors remaining significant after age and sex adjustment into the model. In a sensitivity analysis, re-admissions were excluded (ie, only first admissions were considered). Missing data were not imputed, and where these exceeded 5%, the denominator for the respective variable was reported accordingly.

Unadjusted and adjusted ORs with 95% CIs were calculated for binarised outcomes (length of hospital stay >7 days, increased care needs, and death during admission) for all admissions and in a sensitivity analysis after exclusion of re-admissions. Unadjusted and adjusted hazard ratios (_adj_HRs) for time to death from day of admission to death at 30 days and beyond were calculated using Cox proportional hazards after checking for the proportional hazard assumption, for the cohort overall and after stratification by age group (<65, 65–74, 75–89, and ≥90 years). For those aged 65 years and older, mortality on follow-up was also stratified by comorbid dementia, frailty status, and residence and adjusted for age, sex, illness severity, frailty, and comorbidity burden. In a sensitivity analysis, outcomes were adjusted for pre-admission dependency and previous dementia in place of frailty. SPSS version 29 was used to perform the statistical analyses.

### Role of the funding source

The funders of the study had no role in the study design, data collection, data analysis, data interpretation, or writing of the report.

## Results

**Figure 1:**
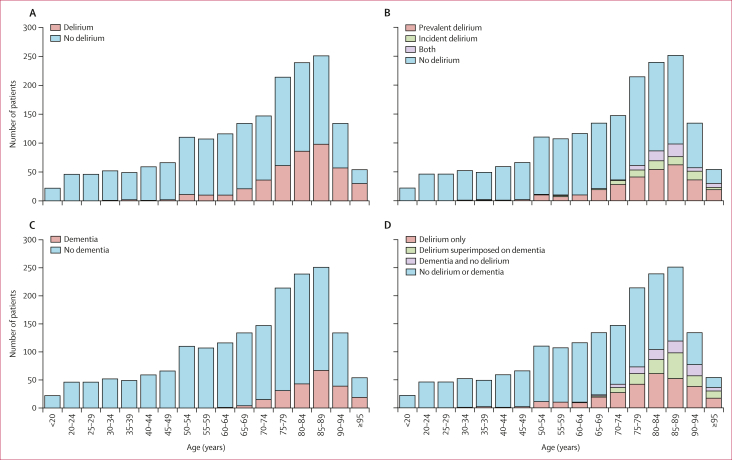
Age-specific delirium and dementia occurrence (A) Delirium occurrence. (B) Delirium occurrence by subtype. (C) Dementia prevalence. (D) Delirium occurrence by dementia status. Charts with specific percentages listed are in the appendix (p 31).

Among 1846 consecutive patients admitted to acute general medicine over the study period (cohort 1 n=240, cohort 2 n=258, cohort 3 n=316, cohort 4 n=283, cohort 5 n=472, and cohort 6 n=277), the median age was 74 years (IQR 55–84; range 16–102), mean age was 68·2 years (SD 20·0), 950 (51·5%) were female, 895 (48·5%) were male, and sex data were missing for one patient (table 1). Any delirium occurred in 426 (23% [95% CI 21–25]) patients, of whom 290 (68% [63–72]) had prevalent delirium and 73 (17% [14–21]) had incident delirium, with 63 (15% [12–19]) having both prevalent and incident delirium (figure 1, appendix p 31). Delirium increased markedly with age, increasing from six (2% [1–4]) of 340 patients younger than 50 years and 31 (9% [6–13]) of 333 at age 50–64 years to 57 (20% [16–25]) of 281 at age 65–74 years, 245 (35% [31–38]) of 704 at age 75–89 years, and 87 (46% [39–54]) of 188 at age 90 years and older (figure 1A, B; appendix pp 32–33). Pre-existing dementia diagnosis was present in 219 (11·9%) of 1846 patients overall, but increased with age from one (0·1%) of 673 patients younger than 65 years to 19 (6·8%) of 281 at age 65–74 years, 141 (20·0%) of 704 at age 75–89 years, and 58 (30·9%) of 188 at age 90 years and older (figure 1C; appendix pp 32–33). Delirium superimposed on dementia (134 [31·5%] of 426 patients with delirium overall) was therefore relatively more common in older versus younger patients.

**Table 1:** Characteristics of cohorts by delirium status

	All (n=1846∗)	Delirium (n=426)	No delirium (n=1420)	p value
**Whole cohort**				
Number of patients	1846∗	426	1420	··
Age, years				
Median (IQR)	74 (55–84)	83 (76–88)	68 (50–81)	<0·001
Mean (SD)	68·2 (20·0)	80·9 (11·3)	64·4 (20·5)	<0·001
Sex†	··	··	··	0·084
Female	950 (51·5%)	235 (55·2%)	715 (50·4%)	··
Male	895 (48·5%)	191 (44·8%)	704 (49·6%)	··
Pre-existing dementia diagnosis	219 (11·9%)	134 (31·5%)	85 (6·0%)	<0·001
Death during admission	119 (6·4%)	63 (14·8%)	56 (3·9%)	<0·001
**Younger than 65 years**				
Number of patients	673	37	636	··
Age, years				
Median (IQR)	49 (35–57)	55 (52–60)	48 (34–57)	<0·001
Mean (SD)	45·7 (13·5)	54·5 (7·3)	45·2 (13·6)	<0·001
Sex†	··	··	··	0·035
Female	341 (50·7%)	25 (67·6%)	316 (49·7%)	··
Male	331 (49·2%)	12 (32·4%)	319 (50·2%)	··
Pre-existing dementia diagnosis	1 (0·1%)	1 (2·7%)	0	<0·001
Death during admission	19 (2·8%)	3 (8·1%)	16 (2·5%)	0·046
**Aged 65 years or older**				
Number of patients	1173	389	784	··
Age, years				
Median (IQR)	82 (75–88)	84 (78–89)	80 (74–86)	<0·001
Mean (SD)	81·1 (8·3)	83·4 (7·9)	79·9 (8·3)	<0·001
Sex	··	··	··	0·32
Female	609 (51·9%)	210 (54·0%)	399 (50·9%)	··
Male	564 (48·1%)	179 (46·0%)	385 (49·1%)	··
Pre-existing dementia diagnosis	218 (18·6%)	133 (34·2%)	85 (10·8%)	<0·001
Death during admission	100 (8·5%)	60 (15·4%)	40 (5·1%)	<0·001

Data are n (%), unless specified otherwise. Approximately 86% of the background Oxfordshire population are White based on the 2021 Census Data.

∗ The 1846 admissions occurred in 1805 patients (41 patients were re-admitted over the study period, 39 had one re-admission, and two had two re-admissions).

† Sex data were missing in one patient.

**Figure 2:**
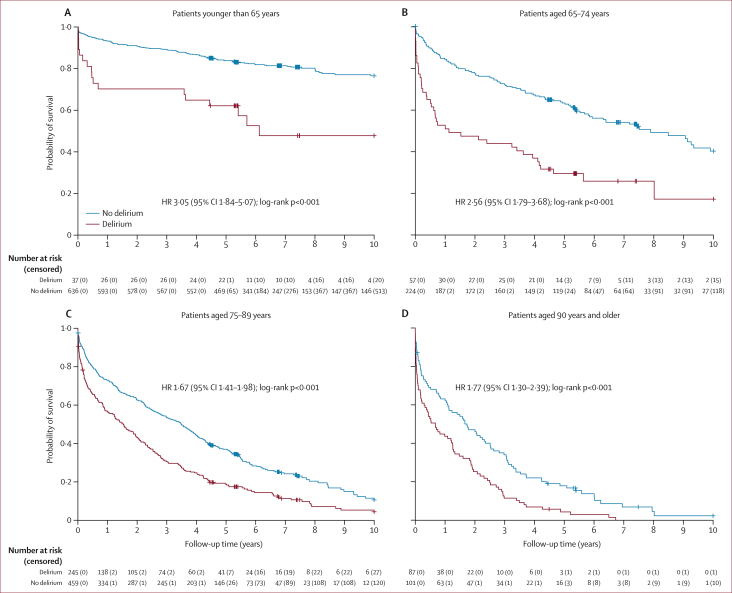
Kaplan–Meier survival curve from admission and up to 10 years' follow-up stratified by age group (A) Patients younger than 65 years. (B) Patients aged 65–74 years. (C) Patients aged 75–89 years. (D) Patients aged 90 years and older. Tick marks on all charts show censored patients. HR=hazard ratio.

Of the 37 patients younger than 65 years with delirium, 31 (84%) had prevalent delirium, four (11%) had incident delirium, and two (5%) had both (appendix pp 34–35). An underlying neurological or neuropsychiatric disorder was present in 28 (76%) of these patients. The most common acute medical conditions were infection (17 [46%] of 37) and metabolic disturbances (seven [19%]; eg, electrolyte imbalances, hyperglycaemia or hypoglycaemia, or acute kidney injury). 18 (49%) of the 37 patients younger than 65 years with delirium had severe illness (SIRS ≥2). In this age group, there were no significant associations of delirium with death during admission (OR 3·42 [95% CI 0·95–12·30]; p=0·060) but the 10-year unadjusted mortality risk was increased in patients with delirium compared with patients with no delirium (HR 3·05 [1·84–5·07]; p<0·001; figure 2A).

In the 1173 patients aged 65 years and older, those with delirium were older and more likely to have dementia than those without delirium (table 1). Patients with delirium also had lower cognitive scores on admission than those without delirium (median MMSE 19·0 [IQR 15·5–25·0] *vs* 25·0 [18·0–28·0]; p=0·0086 and median AMTS 6·0 [3·0–8·0] *vs* 9·0 [8·0–10]; p<0·001).

After adjustment for age and sex, predisposing factors associated with delirium (all p<0·001) included dementia diagnosis, falls history, visual impairment, dependency, comorbidity burden (CCI score) and frailty (HFRS score; table 2). In multivariable analysis, dementia, visual impairment, previous dependency, and frailty remained significant (table 2). Admission clinical parameters associated with delirium (all p<0·001 unless otherwise specified) included low cognitive score, clinical dehydration, abnormal white cell count, C-reactive protein concentration more than 6 mmol/L (p=0·0041), and pressure sore risk (table 2). Low cognitive score, dehydration, and pressure sore risk remained significant in multivariable analysis. Infection was positively associated with delirium and cardiac or other diagnoses were negatively associated with delirium; only cardiac diagnoses remained significant in multivariable analysis (table 2).

**Table 2:** Factors associated with any delirium in patients aged 65 years or older

	Number of patients (n=1173)	Delirium (n=389)	No delirium (n=784)	OR (95% CI)	p value	Age and sex adjusted OR∗ (95% CI)	Adjusted p value∗	Multivariable adjusted OR† (95% CI)	Adjusted p value†
**Demographic factors**									
Age >75 years	871	327 (84·1%)	544 (69·4%)	2·33 (1·71–3·18)	<0·001	··	··	··	··
Female	609	210 (54·0%)	399 (50·9%)	1·13 (0·89–1·44)	0·32	··	··	··	··
Male	564	179 (46·0%)	385 (49·1%)	0·88 (0·69–1·13)	0·32	··	··	··	··
**Medical history**									
Dementia	218	133 (34·2%)	85 (10·8%)	4·27 (3·14–5·81)	<0·001	3·63 (2·65–4·98)	<0·001	2·05 (1·28–3·29)	0·0029
Falls	517	238 (61·1%)	279 (36·0%)	2·84 (2·21–3·65)	<0·001	2·41 (1·86–3·13)	<0·001	1·31 (0·92–1·87)	0·14
Transient ischaemic attack or stroke	217	87 (22·4%)	130 (16·6%)	1·45 (1·07–1·96)	0·018	1·25 (0·91–1·71)	0·16	··	··
Depression	179	75 (19·3%)	104 (13·3%)	1·56 (1·13–2·16)	0·0075	1·68 (1·20–2·34)	0·0025	1·24 (0·79–1·94)	0·36
Other psychiatric history	105	37 (9·5%)	68 (8·7%)	1·10 (0·72–1·68)	0·66	1·19 (0·77–1·83)	0·44	··	··
Visual impairment	175	91 (23·4%)	84 (10·7%)	2·55 (1·84–3·53)	<0·001	2·08 (1·49–2·91)	<0·001	1·61 (1·06–2·45)	0·027
Hearing impairment‡	53	29/294 (9·9%)	24/580 (4·1%)	2·54 (1·45–4·44)	0·0011	1·96 (1·10–3·49)	0·022	1·65 (0·85–3·21)	0·14
More than three medications	914	323 (83·0%)	591 (76·1%)	1·42 (1·03–1·96)	0·034	1·35 (0·97–1·87)	0·077	··	··
More than seven medications	477	149 (38·3%)	328 (41·8%)	0·82 (0·64–1·05)	0·11	0·83 (0·65–1·08)	0·16	··	··
Previous dependency	396	198 (50·9%)	198 (25·3%)	3·05 (2·37–3·95)	<0·001	2·63 (2·02–3·43)	<0·001	1·50 (1·03–2·19)	0·034
Care home	110	63 (16·2%)	47 (5·9%)	3·02 (2·03–4·51)	<0·001	2·60 (1·73–3·92)	<0·001	1·09 (0·60–1·96)	0·78
Charlson Comorbidity Index	··	··	··	1·04 (1·03–1·05)	<0·001	1·04 (1·02–1·05)	<0·001	0·99 (0·97–1·01)	0·37
**HFRS**									
HFRS per point	··	··	··	1·18 (1·15–1·21)	<0·001	1·17 (1·14–1·20)	<0·001	1·13 (1·09–1·17)	<0·001
HFRS category									
Low risk	564	95 (24·4%)	469 (59·8%)	1 (ref)	··	1 (ref)	··	1 (ref)	··
Moderate risk	458	204 (52·4%)	254 (32·4%)	3·97 (2·98–5·29)	<0·001	3·62 (2·70–4·85)	<0·001	··	··
High risk	108	80 (20·6%)	28 (3·6%)	14·11 (8·70-22·88)	<0·001	11·85 (7·24–19·42)	<0·001	··	··
**Clinical parameters**§									
Low cognitive score¶	365	214/280 (76·4%)	151/531 (28·4%)	8·16 (5·84–11·40)	<0·001	7·93 (5·62–11·18)	<0·001	8·26 (5·22–13·08)	<0·001
Clinical dehydration	350	195 (50·1%)	155 (19·8%)	4·10 (3·14–5·35)	<0·001	3·96 (3·02–5·19)	<0·001	3·31 (2·11–5·17)	<0·001
Respiratory rate >20 breaths per min	213	81/290 (27·9%)	132/569 (23·2%)	1·28 (0·93–1·77)	0·13	1·27 (0·92–1·77)	0·15	··	··
Abnormal temperature	295	113 (29·0%)	182 (23·2%)	1·31 (0·99–1·72)	0·056	1·23 (0·93–1·63)	0·15	··	··
Abnormal white cell count	379	155 (39·8%)	224 (28·6%)	1·65 (1·28–2·14)	<0·001	1·64 (1·26–2·13)	<0·001	0·97 (0·57–1·65)	0·92
Sodium <135 mmol/L	273	105/314 (33·4%)	168/631 (26·6%)	1·39 (1·03–1·86)	0·030	1·36 (1·01–1·83)	0·045	1·48 (0·93–2·33)	0·095
C-reactive protein >6 mmol/L	627	245/303 (80·9%)	382/537 (71·1%)	1·71 (1·22–2·41)	0·0020	1·67 (1·18–2·36)	0·0041	1·71 (0·98–3·00)	0·061
Urea:creatinine ratio >100:1	314	120/310 (38·7%)	194/621 (31·2%)	1·39 (1·05–1·85)	0·023	1·27 (0·95–1·70)	0·11	··	··
SIRS ≥2	391	153 (39·3%)	238 (30·4%)	1·47 (1·14–1·90)	0·0033	1·43 (1·10–1·86)	0·0072	0·92 (0·54–1·54)	0·74
PSPS ≥6 or Braden <19	431	229/331 (69·2%)	202/571 (35·4%)	4·10 (3·07–5·48)	<0·001	3·75 (2·80–5·03)	<0·001	2·28 (1·47–3·53)	<0·001
MUST >0	119	58/232 (25·0%)	61/345 (17·7%)	1·55 (1·03–2·33)	0·034	1·40 (0·92–2·11)	0·12	··	··
**Diagnosis**									
Infection	559	257 (66·1%)	302 (38·5%)	3·11 (2·41–4·01)	<0·001	2·90 (2·24–3·75)	<0·001	1·63 (0·93–2·86)	0·086
Cardiac	145	28 (7·2%)	117 (14·9%)	0·44 (0·29–0·68)	<0·001	0·42 (0·27–0·66)	<0·001	0·36 (0·21–0·62)	<0·001
Stroke	53	22 (5·7%)	31 (4·0%)	1·46 (0·83–2·55)	0·19	1·36 (0·77–2·40)	0·30	··	··
Other	479	104 (26·7%)	375 (47·8%)	0·40 (0·31–0·52)	<0·001	0·43 (0·33–0·56)	<0·001	0·53 (0·29–0·97)	0·039
**During admission**									
Urinary incontinence	344	201 (51·7%)	143 (18·2%)	4·79 (3·66–6·27)	<0·001	4·27 (3·25–5·62)	<0·001	3·21 (2·26–4·58)	<0·001
Faecal incontinence	137	82 (21·1%)	55 (7·0%)	3·53 (2·45–5·10)	<0·001	3·07 (2·11–4·46)	<0·001	1·15 (0·70–1·90)	0·58
Bedbound	139	86 (22·1%)	53 (6·8%)	3·91 (2·71–5·65)	<0·001	3·77 (2·58–5·48)	<0·001	2·28 (1·44–3·59)	<0·001
Sleep disturbance	178	130 (33·4%)	48 (6·1%)	7·72 (5·39–11·07)	<0·001	7·46 (5·17–10·76)	<0·001	4·60 (3·05–6·93)	<0·001
Constipation	166	86 (22·1%)	80 (10·2%)	2·48 (1·78–3·46)	<0·001	2·27 (1·61–3·18)	<0·001	1·14 (0·75–1·72)	0·54
Inpatient falls	62	43 (11·1%)	19 (2·4%)	5·01 (2·88–8·73)	<0·001	4·92 (2·80–8·65)	<0·001	1·66 (0·87–3·16)	0·12
Urinary catheter insertion	131	83 (21·3%)	48 (6·1%)	4·15 (2·84–6·07)	<0·001	3·80 (2·59–5·60)	<0·001	2·45 (1·55–3·87)	<0·001
CT brain scanning	335	191 (49·1%)	144 (18·4%)	4·27 (3·26–5·58)	<0·001	4·33 (3·29–5·70)	<0·001	4·15 (3·03–5·69)	<0·001

Data are n (%) or n/N (%) except where otherwise specified. Denominator for respective variables are shown where missing data exceed 5%. AMTS=Abbreviated Mental Test Score. HFRS=Hospital Frailty Risk Score. MMSE=Mini-Mental State Examination. MUST=Malnutrition Universal Screening Tool. OR=odds ratio. PSPS=Pressure Sore Prediction Score. SIRS=systemic inflammatory response syndrome.

∗ Adjusted for age and sex.

† Multivariable analysis performed separately for medical history including Hospital Frailty Risk Score, clinical parameters, diagnosis and during admission factors.

‡ All missing data are from cohort 1 or 2.

§ Clinical parameters are defined as: low cognitive score: AMTS <9 or MMSE <24; abnormal temperature: temperature >38°C or <36°C; abnormal white cell count <4 × 10^9^ cells per L or >12 × 10^9^ cells per L.

¶ Reason for missing data: uncooperative or too unwell n=100, dysphasic n=20, language barrier n=6, inappropriate setting n=3, hearing impairment n=2, not done n=231.

Factors occurring during admission that were strongly related to delirium (all p<0·001) included urinary incontinence, faecal incontinence, being bedbound, sleep disturbance, constipation, inpatient falls, urinary catheterisation, and CT brain scanning (table 2). All remained significant in multivariable analysis except for faecal incontinence, constipation, and inpatient fall (table 2). Factors were broadly similar in prevalent and incident delirium except that low cognitive score on admission was more strongly associated with prevalent delirium, and sleep disturbance, constipation, and inpatient falls were more strongly associated with incident delirium (appendix pp 36–37). Findings were similar after excluding re-admissions (appendix p 38).

After stratification by comorbid dementia status, some frailty markers (history of falls or depression, previous dependency, and low cognitive score), multimorbidity (CCI), hyponatraemia (ie, sodium <135 mmol/L), and constipation were only associated with delirium in those without comorbid dementia after adjustment for age and sex (appendix pp 39–40). Other markers of frailty were associated with delirium regardless of dementia status, but associations were stronger in those without dementia for frailty (HFRS), pressure sore risk, urinary incontinence, being bedbound, sleep disturbance, inpatient falls, and CT brain scanning. Notably, stroke diagnosis was associated with delirium only in those without dementia.

The odds of poor outcome were higher for patients with versus without delirium after adjustment for age and sex (appendix pp 41–42). All outcomes except new placement (length of hospital stay >7 days, increased care needs at discharge, and death during admission) remained robust to further adjustment for illness severity, comorbidity burden, and frailty (all p<0·001): length of stay longer than 7 days (_adj_OR 2·48 [95% CI 1·84–3·35]), increased care needs at discharge (2·41 [1·70–3·40]), and death during admission (2·45 [1·52–3·94]), although after stratification by comorbid dementia status, associations were only significant in those without dementia (appendix p 43). Findings were similar after replacing frailty with pre-admission dependency and comorbid dementia in the fully adjusted model (all p<0·001) and after excluding re-admissions (appendix pp 41–42)

During 3764 person-years of follow-up in those aged 65 years and older, there were 882 deaths. There were 4·1 deaths (95% CI 3·7–4·6) per 10 person-years with delirium and 1·8 deaths (1·7–2·0) per 10 person-years without delirium. The increased risk of death in the delirium group was highest in the immediate postadmission period and attenuated thereafter, but was maintained up to 10 years of follow-up (_adj_HR 2·03 [95% CI 1·40–2·97] for 30-day mortality *vs* 1·52 [1·30–1·77] for 10-year mortality; figure 2B–D; appendix p 44). The proportion with delirium surviving at 5 years and 10 years is shown in appendix p 45.

**Figure 3:**
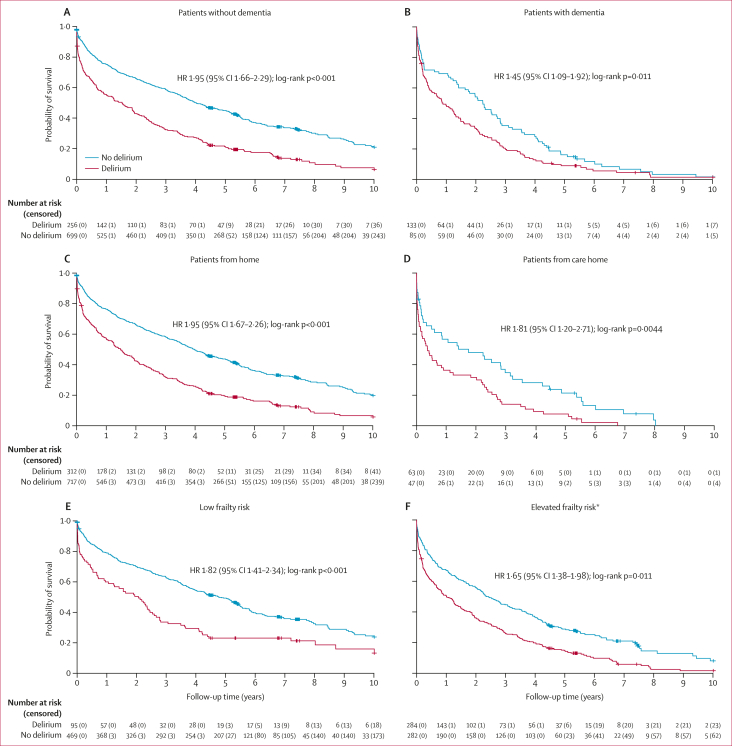
Kaplan–Meier survival curve from admission up to 10 years of follow-up in patients aged 65 years and older with and without delirium stratified by status of comorbid dementia, pre-admission residence, and frailty (A) Patients without dementia. (B) Patients with dementia. (C) Patients from home. (D) Patients from care home. (E) Low frailty risk. (F) Elevated frailty risk. Tick marks on all charts show censored patients. HR=hazard ratio. ∗Elevated frailty risk includes patients with moderate and high frailty risk.

Associations between delirium and inpatient death were strongest, in patients aged 65–74 years (_adj_OR 4·38 [95% CI 1·18–16·31]; p=0·028 *vs* 1·96 [1·02–3·75]; p=0·043 at age 75–89 years and 2·86 [1·14–7·16]; p=0·025 at age 90 years and older; appendix p 46). After stratification by clinical characterestics, delirium was associated with excess risk of death during admission in those without but not in those with dementia (_adj_OR 3·02 [1·73–5·25]; p<0·001 *vs* 1·47 [0·58–3·75]; p=0·42); in patients living at home pre-admission but not in care home residents (2·85 [1·64–4·93]; p<0·001 *vs* 1·49 [0·47–4·69]; p=0·50) and in people with less and moderate frailty but not high frailty (appendix pp 46–47). The relatively greater mortality with delirium in younger versus older patients, those without versus with dementia, living at home versus care home, and more robust versus frail was maintained throughout follow-up, although attenuated by 2 years (figure 3; appendix pp 46–47).

## Discussion

Delirium occurred in a quarter of patients in acute general medicine and increased markedly with age from 2% at younger than 50 years and one-tenth at age 50–64 years, to a fifth of those aged 65–74 years, a third of those aged 75–89 years, and half of those aged 90 years or older. Most delirium was prevalent rather than incident. Predisposing factors in those aged 65 years or older, after age and sex adjustment, included pre-existing dementia, and also frailty, depression, and multimorbidity, with associations generally stronger in people without dementia than in people with dementia. Precipitants included infection, clinical dehydration, hyponatraemia, raised inflammatory markers, and SIRS 2 or greater. Delirium was an independent predictor of poor outcomes with a two to three times greater likelihood of length of hospital stay beyond 7 days and increased care needs at discharge. The excess mortality risk associated with delirium was highest earlier in follow-up but persisted out to 10 years of follow-up and was highest in patients aged 65–74 years, those without comorbid dementia or frailty, and those resident at home.

The occurrence of delirium in our study was similar to the pooled results reported previously,^3^ but the mean age in our study was lower (80 years for pooled results *vs* 68·2 years in this study), suggesting a higher measured age-specific occurrence. Given the strong relationship with age, delirium will become even more common as the population ages. In addition, the proportion of patients with delirium in hospital will likely be increased by the development of admission avoidance services (eg, same day emergency care) which are predominantly applicable to younger, fitter people and less suitable for patients with delirium and frailty.^16^ Less than one fifth of delirium was solely incident, in line with previous studies.^3^ Although multicomponent interventions can prevent incident delirium by around 30%,^11^^,^^17^ there is little evidence for an effect on prevalent delirium.

Data on delirium in younger patients are scarce.^8^^,^^18^^,^^19^ A South African study (n=808, >80% younger than 70 years) found urinary catheter and central nervous system disease to be predictors of delirium.^19^ In our study, 84% of the younger patients with delirium had underlying neurological or neuropsychiatric conditions, half had severe illness, and half had infection. In the older patients, predisposing (vulnerability) factors and precipitating factors linked to acute illness were important associates of delirium, in line with previous reports. However, in those without dementia, delirium identified a frailer, more multimorbid group with inpatient falls. In contrast, although frailty and being dependent also increased the odds of delirium in those with dementia, associations were weaker because frailty is common in patients with dementia irrespective of delirium. Similarly, hyponatraemia, constipation, and stroke were precipitants of delirium only in those without dementia, possibly again because of frequent occurrence in patients with dementia irrespective of delirium status. Of note, we measured frailty using the HFRS, a recently developed tool using ICD-10 diagnostic coding with known prognostic value,^20^ because prospectively applied tools such as the Clinical Frailty Scale were unavailable. Two previous studies found associations of HFRS with delirium in patients with hip fractures^21^ and in geriatric medicine,^22^ but comorbid dementia status was not considered.

Delirium was associated with long length of hospital stay and increased care needs on discharge after adjustment for illness severity, frailty, and comorbidity. A previous meta-analysis in medical inpatients^4^ found conflicting findings, although a recent study of acute hospital admissions reported a 3·5-day increase in length of hospital stay with delirium after adjustment for age, sex, dementia, specialty, and frailty.^23^ Longer hospital stays in patients with delirium could be related to the need for care at home, rehabilitation, or care home transfer,^24^ but illness severity, frailty, and complications including inpatient falls might also contribute.

That mortality after delirium was highest earlier in follow-up, with a two to three times increased risk in the first 2 years, is consistent with studies from multiple settings including general medicine.^6^ However, previous studies did not have long-term follow-up with adjustment for key confounders, and stratification by key characteristics. We found the increased risk of death persisted over 10 years, albeit attenuating with time, and was relatively greater in patients aged 65–74 years, those without dementia, home residents, and more robust individuals, in keeping with the scarce existing evidence.^25^^,^^26^ Delirium might be a particular marker of vulnerability in younger people, whereas older patients have an overall high prevalence of frailty and multimorbidity and high absolute mortality risk irrespective of delirium status. Alternatively, the greater physiological insult needed to precipitate delirium in more robust individuals might result in increased risk of death.

The main strengths of our study include the large, well characterised, inclusive cohort with prospective ascertainment of delirium by experienced physicians enabling reliable age-specific delirium estimates, adjustment for important covariates, and long-term follow-up. Our study also has several limitations. First, the single-centre study design from general medicine in a high-income country might affect generalisability. Notably, the occurrence of incident delirium was low, in contrast to postoperative settings, limiting comparison of incident versus prevalent delirium and understanding of factors as causes or consequences of delirium. Second, there were fewer available data in patients younger than 65 years, although we hand-searched records to characterise those with delirium. Third, we did not evaluate delirium subtype, duration, and severity, which could have influenced associations and outcomes. Fourth, stratification by dementia was based on pre-existing dementia diagnosis rather than prospective diagnosis during admission and some patients likely had undiagnosed dementia. Fifth, delirium diagnosis was not made by a dedicated research team but by the senior physicians providing patient care throughout admission using all available information. Finally, we used the HFRS to measure frailty, which is dependent on ICD-10 coding accuracy; however, this meant that frailty status was available for all patients.

Our findings have important implications for the planning and delivery of health services. Delirium occurred sufficiently often to justify on-admission screening for all patients aged 65 years and older, supporting current guidelines.^27^ In addition, most delirium, especially in patients aged 65–74 years, occurred in the absence of a pre-existing dementia diagnosis, showing the need for routine cognitive screening, which we recently showed to be feasible at scale through electronic patient record implementation.^28^^,^^29^ Staffing and training need to be sufficient to meet the complex needs of those with delirium, including around falls prevention, capacity, consent,^30^ and early discharge planning. Hospital systems and clinical trials should focus particularly on patients aged 65–74 years and previously independent people without dementia in whom the effect of delirium on outcomes, including future cognitive decline, is greatest.^31^ Finally, given that delirium is a risk factor for dementia, our findings on long-term survival after delirium will inform the likely dementia burden and sample size calculations for future trials of preventive treatments.

In conclusion, delirium occurrence in patients in acute medicine settings increases sharply from age 65 years upwards, usually occurs in the absence of comorbid dementia diagnosis, and is most often present on admission rather than arising de novo during admission. Delirium identifies a frail, multimorbid, acutely unwell group overall but the relative hazard of long-term adverse outcomes is greater in those aged 65–74 years, fitter, more robust patients without dementia. Effective treatment is lacking and future studies on the pathophysiology of delirium and its subtypes are required to inform future therapies.

## Data sharing

At present, the ethics and governance approvals for ORCHARD-EPR cover use by University of Oxford and Oxford University Hospitals NHS Foundation Trust researchers only. Fully anonymised data may be available in the future pending revised ethics and governance arrangements.

## Declaration of interests

JMG is supported by the Oxford-Medical Research Council Doctoral Training Partnership. ELB was supported by the Rhodes Trust and the Canadian Institutes for Health Research. ELB received support for attending meetings or travel from the Guarantors of Brain Travel Award and Alzheimer’s Research UK Travel Award. STP is supported by the National Institute for Health and Care Research (NIHR) Oxford Biomedical Research Centre and NIHR Invention for Innovation programme grant NIHR204290, which also funds ORCHARD-EPR. All other authors declare no competing interests.
