# Supplementary material for: Occurrence, associated factors, and outcomes of delirium in patients in an adult acute general medicine service in England: a 10-year longitudinal, observational study
Source: Lancet Healthy Longev. 2025 Jul;6(7):None. doi: 10.1016/j.lanhl.2025.100731 (PMC12316638; doi:10.1016/j.lanhl.2025.100731)
Supplement: Supplementary appendix [file mmc1.pdf]

THE LANCET
Healthy Longevity

Supplementary appendix

This appendix formed part of the original submission and has been peer reviewed.
We post it as supplied by the authors.

Supplement to: Gan JM, Boucher EL, Lovett NG, et al. Occurrence, associated factors, and outcomes of delirium in patients in an adult acute general medicine service in England: a 10-year longitudinal, observational study. *Lancet Healthy Longev* 2025. https://doi.org/10.1016/j.lanhl.2025.100731

Contents

Supplementary Methods and Results of Literature Search 2
Methods of updated literature search 2
Literature search results 3
Supplementary Table of Previous Studies 4
Supplementary Table 1. Details of studies included in recent meta-analysis on delirium occurrence and from updated literature search. 4
Supplementary Table 2. Selected studies (of acute general (internal) medicine or geriatric wards) included in recent systematic review aimed at identifying delirium risk factors and from updated literature search 11
Supplementary Table 3. Selected studies (of acute general (internal) medicine or geriatric wards) included in meta-analysis on delirium-associated mortality and from updated literature search. 17
References 24
Supplementary Methods 29
Description of the acute medical admissions service at the John Radcliffe Hospital 29
Cognitive screen and delirium diagnosis 30
Supplementary Results 31
Supplementary Figure 1. Venn diagram displaying numbers with prevalent delirium, incident delirium and both across the entire adult age range 31
Supplementary Figure 2. Age-specific a) delirium occurrence, b) delirium occurrence by subtype, c) dementia prevalence, and d) delirium occurrence by dementia status. 31
Supplementary Figure 3. Delirium occurrence vs dementia prevalence by age group 32
Supplementary Figure 4. Venn diagram displaying number with delirium, dementia and delirium superimposed on dementia stratified by age groups 32
Supplementary Figure 5. Age-specific occurrence of delirium only, delirium superimposed on dementia and dementia only ... 33
Supplementary Table 4. Characteristics of younger patients with delirium. 34
Supplementary Table 5. Factors associated with prevalent delirium only in patients aged >65 years (n=259) 36
Supplementary Table 6. Factors associated with incident delirium only in patients aged >65 years (n=69) 37
Supplementary Table 7. Factors associated with delirium in patients aged >65 years excluding readmissions 38
Supplementary Table 8. Factors associated with delirium in patients aged >65 years stratified by comorbid dementia status ... 39
Supplementary Table 9a. Outcomes in any delirium (patients aged >65 years). 41
Supplementary Table 9b. Sensitivity analysis of outcomes associated with delirium in patients aged >65 years, replacing frailty with pre-admission dependency and comorbid dementia. 41
Supplementary Table 10a. Outcomes in prevalent delirium only (patients aged >65 years, n=259) 42
Supplementary Table 10b. Outcomes in incident delirium (patients aged >65 years, n=69) 42
Supplementary Table 11. Outcomes in delirium (patients aged >65 years) excluding patients with readmissions 42
Supplementary Table 12. Outcomes in delirium (patients aged >65 years) stratified by comorbid dementia status. 43
Supplementary Figure 6. Kaplan-Meier survival curve from admission and up to 10 years follow-up for all patients aged >65 years 44
Supplementary Table 13a. Mortality risk across follow-up time 44
Supplementary Table 13b. Sensitivity analysis of mortality risk across follow-up time, replacing frailty with pre-admission dependency and comorbid dementia. 44
Supplementary Table 13c. Mortality risk of patients who survived preceding follow-up time. 44
Supplementary Table 14. Proportion of patients aged > 65 years who were alive after 5 years and 10 years of follow-up from admission. 45

Supplementary Table 15a. Inpatient mortality risk in patients with delirium compared to those without delirium stratified by age group, dementia status, residence and frailty. 46

Supplementary Table 15b. Mortality risk over 10-year follow-up period in patients with delirium compared to those without delirium stratified by age group..... 46

Supplementary Table 15c. Mortality risk over 10-year follow-up period in patients with delirium compared to those without delirium stratified by dementia status..... 46

Supplementary Table 15d. Mortality risk over 10-year follow-up in patients with delirium compared to those without delirium stratified by residence status...... 47

Supplementary 15e. Mortality risk over 10-year follow-up in patients with delirium compared to those without delirium stratified by frailty status. 47

Supplementary Methods and Results of Literature Search

Methods of updated literature search

Updated literature search on delirium occurrence

The systematic review and meta-analysis by Gibb et al[1,2] of 33 studies reported delirium occurrence of 23% (95% CI 19-26%) in medical inpatients. We updated this review by searching Medline using similar search terms (see below) from one year prior to the previous end date (2018) to 28th February 2025. We included prospective cohort or cross-sectional studies of adults aged ≥18 years who were admitted to acute general (internal) medicine or geriatric medicine. Delirium diagnosis was required to be ascertained in accordance with the Diagnostic and Statistical Manual of Mental Disorders (DSM) or the International Statistical Classification of Diseases (ICD) criteria by trained personnel (in accordance with the recent systematic review). We excluded studies restricted to participants with COVID-19 or referred solely to liaison psychiatry services, studies conducted in mixed settings or specialty-specific wards (unless delirium occurrence from acute general (internal) medicine or geriatric medicine population were specifically reported) and studies that diagnosed delirium retrospectively by chart review or routine clinical ascertainment. We found eight articles that met inclusion criteria (Supplementary Table 1)

Search terms: (Delirium[Title]) AND (epidemiology[Title/Abstract] OR prevalence[Title/Abstract] OR incidence[Title/Abstract] OR occurrence[Title/Abstract] OR rate[Title/Abstract]) Filters: Case Reports, Clinical Study, Clinical Trial, Dataset, Meta-Analysis, Multicenter Study, Observational Study, Overall, Randomized Controlled Trial, Case Reports, Clinical Study, Clinical Trial, Dataset, Meta-Analysis, Multicenter Study, Observational Study, Overall, Randomized Controlled Trial

Updated literature search on delirium risk factors

The systematic review by Ormseth et al[3] found 315 studies on risk factors associated with delirium, of which 38 were from acute general (internal) or geriatric medicine. We updated this review by searching Medline using similar search terms (see details below) from one year prior to the previous end date (2020) to 28th February 2025. We included prospective cohort or case-control studies with the objective of determining risk factors associated with delirium. Studies that met the following criteria were included: i) population aged ≥18 years, ii) admitted to acute general (internal) medicine or geriatric medicine and iii) delirium was diagnosed using the DSM, Confusion Assessment Method (CAM), Delirium Rating Scale (DRS) or DRS-Revised-98 by trained personnel (in accordance with the recent systematic review) and iv) multivariate analysis were performed to determine independent risk factors of delirium. The exclusion criteria were: i) delirium diagnosis by bedside nurse, ii) studies restricted to patients with COVID-19, or iii) studies in mixed populations, unless risk factors associated with delirium from acute general (internal) medicine or geriatric medicine population were specifically reported. We found 11 articles that met inclusion criteria (Supplementary Table 2).

Search terms: (Delirium[Title]) AND ((causality OR disease susceptibility[MeSH Terms]) OR (risk[Title/Abstract] OR factor*[Title/Abstract])) Filters: Case Reports, Clinical Study, Clinical Trial, Dataset, Meta-Analysis, Multicenter Study, Observational Study, Overall, Randomized Controlled Trial, Case Reports, Clinical Study, Clinical Trial, Dataset, Meta-Analysis, Multicenter Study, Observational Study, Overall, Randomized Controlled Trial

Updated literature search on delirium-associated mortality

The systematic review and meta-analysis by Aung Thein et al[4] reported pooled unadjusted OR for delirium-associated mortality of 3.64, 95% CI 2.99-4.44, across 29 studies in the medical setting. We updated this review by searching Medline using similar search terms (see details below) from one year prior to the previous end date (2017) to 28th February 2025. We included studies of adults aged ≥18 years who were admitted to acute general (internal) medicine or geriatric medicine. Prospective delirium ascertainment using validated diagnostic instrument (including but not limited to DSM, CAM, 4AT) was required. We excluded i) studies restricted to patients with COVID-19, ii) studies in mixed populations, unless delirium-associated mortality in acute general (internal) medicine or geriatric medicine population were specifically reported. We found 13 articles that met inclusion criteria (Supplementary Table 3).

Search terms: (Delirium[Title]) AND (mortality[Title/Abstract] OR death[Title/Abstract] OR prognos*[Title/Abstract] OR outcome*[Title/Abstract]) Filters: Case Reports, Clinical Study, Clinical Trial, Dataset, Meta-Analysis, Multicenter Study, Observational Study, Overall, Randomized Controlled Trial, Case Reports, Clinical Study, Clinical Trial, Dataset, Meta-Analysis, Multicenter Study, Observational Study, Overall, Randomized Controlled Trial

Literature search results

Delirium occurrence

From 2018 onwards, we identified eight studies reporting delirium occurrence. All studies ascertained delirium in accordance to the DSM-V criteria. Four studies were conducted in the acute or internal medicine wards, two studies in geriatrics wards and two studies included both settings. Study participants were aged ≥60 years. Dementia prevalence ranged from 23%-37%, where reported. Delirium occurrence ranged from 11.6%-34.8%. Only one study reported the prevalence and incidence of delirium and found delirium prevalence to be almost two-times that of delirium incidence[5]. Two studies reported the frequency of delirium motor subtypes[6,7]. No study reported delirium occurrence over the full adult age range or by age group.

Delirium risk factors

From 2020 onwards, we identified 11 studies on risk factors associated with delirium with a total of 1,066 delirium cases. Five studies were performed in acute medical wards and six were conducted in geriatrics wards. Only one study included the full adult age range[8]. Eight studies assessed multiple demographic and clinical risk factors associated with delirium and three studies determined specific potential factors associated with delirium (i.e. single vs multiple bed rooms[9], IGF-1/GH level[10], stress hyperglycaemia and MRI-visible perivascular spaces[11,12]). Most studies (10 out of 11) were selective and had set exclusion criteria and/or required consent from patient to be included. No study stratified patients by comorbid dementia status.

Delirium associated-mortality

From 2016 onwards, we identified 13 studies that reported the mortality risk associated with delirium. Nine studies were from general or internal medicine wards, four studies were from geriatric wards. Only one study included participants from the full adult age range and reported a higher risk of death over 12-months in the delirium group without adjusting for confounders. Other studies included older participants with minimum age of 60 years, of which six studies did not adjust for any of the three confounders of comorbidity burden, illness severity or frailty and none of the remainder seven studies adjusted for all three confounders of comorbidity burden, illness severity or frailty. One study conducted in the U.K. among acute medical patients had mortality data follow-up of just over 4 years[13] and demonstrated increased risk of death with delirium (HR 1.55, 95% CI=1.10-2.18) after adjustment for age, sex, Charlson Comorbidity Index, Waterlow and APACHE II. No study stratified mortality outcome analysis by important clinical characteristics e.g. comorbid dementia status.

Supplementary Table of Previous Studies

Supplementary Table 1. Details of studies included in recent meta-analysis on delirium occurrence and from updated literature search.

Author	Setting	Study Design	Sample Size	Age (years)	Dementia prevalence	Inclusion	Exclusion	Consent process	Prevalence	Incidence	Occurrence
**Botero Urrea 2024**[6]	Internal Medicine Ward	Prospective cross-sectional	422	≥ 60 years<br><br>Mean Delirium= 82.6<br><br>No delirium = 73.6	24.9% (105)	Admitted during the preceding 24-72 h	Stupor, coma, severe communication difficulties (e.g., severe deafness, aphasia)	Consent or proxy required.<br><br>(n=17 no consent/ no family or caregiver)	24.6% (104)- DSM-5-TR<br><br>Note:<br>22.0% (93)- DRS-R98<br><br>27.7% (117)- DDT-Pro	N/A	24.6% (104)- DSM-5-TR<br><br>Note:<br>22.0% (93)- DRS-R98<br><br>27.7% (117)- DDT-Pro
**Pouw 2023**[14]	Acute geriatric ward at two sites<br><br>Data from ED not included here	Prospective observational/ Diagnostic accuracy study	71	≥ 65<br><br>Median (IQR)= 83 (78-88)	22.5% (16)	Convenience sample	Non-Dutch speaking, acute life-threatening illness, communication difficulties (e.g., severe hearing impairment)	Consent required (verbal).<br><br>No mention about proxy or number refused.	11.6%	N/A	11.6%- DSM-IV
**Paddick 2023**[15]	Internal medicine department	Nested cross-sectional	66	≥60<br><br>Median (IQR)= 75 (66-82)	34.8% (23)	Consecutive admission	Died/ discharged before consent was obtained	Consent required.<br><br>No mention about proxy or number refused.	33.3% (22)	N/A	33.3% (22) - DSM-V
**Geriatric Medicine Research Collaborative 2023**[16]<br><br>(Round 1 cohort- likely some overlap with study published from same group in 2019 as listed below)	Multi-centre across various specialty, including acute medicine and geriatric medicine<br><br>Data from other specialties not included in this table	Prospective observational	Acute medicine =918<br><br>Geriatric medicine = 599	≥ 65 years<br><br>No breakdown by specialty	No breakdown by specialty	Round 1: Emergency admission during the 48h period before data collection on 14/03/2018 and still in hospital at the time of assessment.<br><br>Round 2: Emergency admission between 00:00 and 23:59 on 14/09/2018.<br><br>Round 3: Emergency admission 48–72 h before 8 am on 13/03/2019	Admitted to critical care, imminently dying, elective admissions, clinical records not obtainable, logistical issues, incomplete frailty data.	Not required.	Acute medicine = 18.2% (167)<br><br>Geriatric medicine= 28.0% (168)	N/A	Acute medicine = 18.2% (167)<br><br>Geriatric medicine= 28.0% (168)<br><br>DSM-V
**Franco 2021**[17]	Internal Medicine Ward	Prospective cross-sectional	233	≥ 60	36.5% (85)	Consecutive admission, assessed within 12–48h of admission	Stupor/coma, severe hearing difficulty, non-Spanish speaker	Consent or proxy required.	24.9% (58)	N/A	24.9% (58)<br><br>DSM-V

Author	Setting	Study Design	Sample Size	Age (years)	Dementia prevalence	Inclusion	Exclusion	Consent process	Prevalence	Incidence	Occurrence
								(n=19 no consent, communication issues or stupor/coma)			
**Franco 2020[7]**	Internal Medicine Wards	Cross-sectional	200	≥60<br><br>Mean No delirium= 73.4/8.2<br><br>Delirium= 80.9/7.7<br><br>Sub-syndromal delirium= 78.2/9.4	37% (74)	Consecutive evaluated participants, assessed within 12–48 h of admission from Monday to Saturday	Stupor/coma, severe language or hearing difficulty	Consent or proxy required.	25.0% (50)- DSM-V<br><br>Note:<br>19.5% (39)- TMF<br><br>20.0% (40)- DRS-R98	N/A	25.0% (50)- DSM-V<br><br>Note:<br>19.5% (39)- TMF<br><br>20.0% (40)- DRS-R98
**Lagarto 2020[5 c]**	Acute Male Geriatric Ward	Prospective observational	269	≥ 65	43.5% (117)	Unplanned acute admission, available caregiver to be interviewed	Sensory deficits, aphasia, clinically unstable, hospital stay <48h	Consent/ legal representative required.	15.4% (35/227)-<br><br>n=42 could not be cognitively tested (RASS < -3)	9.2% (23/249)	23.4% (58/248)<br><br>DSM-V
**Geriatric Medicine Research Collaborative 2019[18]**	Multi-centre across various specialty, including acute medicine and geriatric medicine<br><br>Data from other specialties not included here	Prospective observational	Acute medicine = 648<br><br>Geriatric medicine = 265	≥ 65 years<br><br>No breakdown by specialty	No breakdown by specialty	Admitted between 08:00 on 12/03/2018 and 07:59 on 14/03/2018, still in hospital during assessment, available data on 4AT score and delirium status	Admitted to critical care, approaching end of life, logistical issue (e.g. undergoing operation, not at bedside)	Not required.	Acute medicine= 16.4% (106)<br><br>Geriatric medicine= 22.3% (59)	N/A	Acute medicine= 16.4% (106)<br><br>Geriatric medicine= 22.3% (59)<br><br>DSM-V
**Bellelli 2018[19]**	Acute geriatric unit	Cross-sectional	588	≥70 years<br><br>Mean 80.9	12% (68)	Consecutive admissions (multiple hospitals).	Incomplete data, no proxy available for consent.	Consent required (*"many patients due to delirium were excluded"*)	3.9% (23)	N/A	3.9% (23)
**Yam 2018[20]**	Acute General Medical Ward	Prospective Cohort	575	≥65 years<br><br>Mean 80.8	NR	Admissions to general medical wards.	Direct admissions to ICU/ CCU/ acute stroke unit; coma/ persistent vegetative state; severe aphasia; clinically unstable/too unwell; previously recruited; non-Chinese ethnicity	Informed consent or from informant<br><br>(n=26 refused, but none had delirium)	15.8% (91)<br><br>(within 6 hours of admission)	N/A	15.8% (91)

Author	Setting	Study Design	Sample Size	Age (years)	Dementia prevalence	Inclusion	Exclusion	Consent process	Prevalence	Incidence	Occurrence
**Chan 2016**[21 a,c]	NIPPV unit	Prospective	153	All age ≥18<br><br>Mean 74.2	7.8% (12)	Admitted to respiratory wards for acute respiratory failure with NIPPV administered	Coma; unavailable within 48h of admission	Consent required (n=3 no proxy, n=3 declined)	26% (40/153)<br><br>(within 48 hours of NIPPV use)	6% (9)<br><br>(reassessed every 48 hours until discharge, death, or up to 14 days)	32% (49)
**Grandahl 2016**[22 a]	Cancer ward	Cross-sectional	81	≥18 years<br><br>Mean 68.5	NR	Histologically verified cancer diagnosis.	Non-Danish speaking; previously in study	Consent required	N/A	N/A	33% (27)
**Jackson 2016**[23]	Acute medicine	Prospective cohort	1327	≥70 years	NR	Unplanned admissions to acute medicine, screened for delirium	Not screened for delirium	No consent required for determining delirium occurrence.	N/A	N/A	17% (228)
**Kozak 2016**[24 a]	Non-intensive stroke unit	Prospective	60	≥18 years<br><br>Mean age 66.2	NR	Consecutive admission, within first 24 hour of acute ischemic stroke onset	TIA/ ICH/ SAH; reduced GCS; severe aphasia or dysphasia; history of brain tumour/ MI; renal dysfunction; symptomatic PAD; immunosuppressed; GI inflammatory disease; autoimmune/ rheumatic disease, metabolic syndrome recent infection/ trauma or surgery/antidepressant use	Consent required.	N/A	N/A	18.3% (11)
**Adamis 2015**[25]	Acute medical admission	Prospective observational study	200	≥70 years<br><br>Mean age 81.1	63% (126-pre-existing cognitive impairment)	Within 3 days of admission	Been in hospital >3 days; readmission to unit; severe aphasia; intubated; severe sensory impairment; non-English speaking	Consent required (n=8 declined)	DSM-IV criteria: 19.5% (39)<br><br>DSM-V criteria: 13.0% (26)-<br><br>Within 3 days of admission	N/A	20% (39)
**Holtta 2015**[26]	Acute geriatric Wards	Prospective	95	≥ 70 years<br><br>Mean age 86.6	100%	Consecutive admission; patients with dementia	Coma	Consent required.	N/A	N/A	43.2% (41)
**Pendlebury 2015**[27 a,b,c]	Acute medical inpatients	Prospective, observational	503	All age<br><br>Median 72	10% (51)	Admissions to acute medical unit	NIL	No consent required	14% (71)	6% (30)	20% (101) Note: 17 had both prevalent and incident delirium
**Uchida 2015**[28 c]	Elderly cancer patients	Prospective	61 on admission	≥65 years<br><br>Mean 72	NR	Consecutive admission; within 4 days of admission;	Too ill to complete the survey; non-Japanese speaking	Consent required (n=12 refused)	43% (26/61)	20% (7/35)	54% (33/61)

Author	Setting	Study Design	Sample Size	Age (years)	Dementia prevalence	Inclusion	Exclusion	Consent process	Prevalence	Incidence	Occurrence
						incurable lung/ gastroenterological cancer; years; planned admission of ≥2 weeks; ECOG Performance Status score ≥ 2			(within 4 days after admission)	(2 weeks after admission)	
**Bonetti 2012[29]**	Internal medicine/ Geriatric unit	Observational	578	>64 years<br><br>Mean age 82	NR		NIL	No consent mentioned.	N/A	N/A	24% (140)
**Praditsuwan 2012[30 c]**	General Medical Ward	Prospective Observational	225	≥70 years<br><br>Mean age 78	41.8% (94)	Consecutive	Intubated, communication difficulty, uncooperative, transferred to other units, death within 48 h, too unwell	Consent required.	40.4% (91)<br><br>(within 24 hours of admission)	8.4% (19)<br><br>(reassessed every 48 hours until delirium occurred or until discharge)	48.9% (110)
**Thomas 2012[31]**	Academic Geriatric Hospital	Prospective Cohort	79	≥80 years<br><br>Mean age 84.1	75% (59)		Global aphasia; terminal condition	Consent required. (n=14 refused)	28%<br><br>(on third day after admission)	N/A	28%
**Travers 2012[32 c]**	General Medical Ward	Prospective observational study	294	≥70 years;	26% (76)	Expected stay of ≥48 hours; Sunday-Thursday admission; maximum of three patients recruited daily at any one hospital (computer generated random selection)	Transferred from another hospital/ ward, admitted >48 h previously; immunocompromised and isolated; imminent death	Consent required (unclear how many from general medical ward cohort refused)	12.6% (37/294)<br><br>(on admission)	7% (18/257)<br><br>(delirium not present on admission)	19% (55/294)
**Paci 2008[33a]**	Stroke unit	Observational	150	Mean age 67.5±12.5	NR	Admissions to the stroke unit during the first 5 days of hospitalisation	NIL	No consent mentioned.	N/A	20% (30)	20% (30)
**Sheng 2006[34]**	Acute stroke	Cohort study	156	≥65 years<br><br>Mean age 79.2	7.7%(12)	Consecutive admission; delirium within 3 days of stroke	TIAs; SAH; previous severe head trauma or neurosurgery; stroke due to tumour or cerebral venous sinus thrombosis; uncertain delirium diagnosis (due to coma, aphasia, advanced dementia, acute psychosis)	Consent required.	N/A	N/A	25% (39) (within 72 hours of stroke onset)
**Lundstrom 2005[35]**	General Internal Medicine	Prospective intervention study	400	≥70 years<br><br>Mean Control ward= 80.7<br><br>Interventio n ward = 79.4	4.5% (18)	Consecutive admission	-	Consent required.	31% (125)	N/A	31% (125)

Author	Setting	Study Design	Sample Size	Age (years)	Dementia prevalence	Inclusion	Exclusion	Consent process	Prevalence	Incidence	Occurrence
**Laurila 2004**[36]<br><br>(Reported as Pitkala on most recent review[1] but no referenced. Likely to be same study referenced in earlier review[2] as Laurila)	Acute geriatric wards<br><br>Data from nursing home resident not included here.	Cross-sectional	230	≥70 years<br><br>Mean 88.4	45.2%		Coma	Consent required (Overall including nursing home resident population- n=23 refused, n=29 proxy unavailable)	N/A	N/A	34.8%
**Laurila 2004**[37]	Acute geriatric wards	Cross-sectional	219	≥70 years	40% (88)		Coma<br><br>Review of medical records not done, n=11	Consent required. (n=11 no consent)	N/A	N/A	35% (77)
**Cole 2002**[38 c]	General medical unit	Randomised Trial	1925	≥65 years<br><br>Mean Usual care=82.0<br><br>Interventio n group=82.7	Only in people who consent to the randomised trial (58%, 131/227)	Consecutive admission	Stroke, >48 hours in ICU or CCU; admitted to geriatric/ oncology service/ long-term care unit, language barrier, communication difficulties, non-Montreal resident, discharged, dead	No consent required for determining delirium occurrence.	12.6% (243/1925)<br><br>(within 24 hours of admission)	3.3% (56/1682)<br><br>(delirium screen after a week in patients without prevalent delirium)	15.5% (299/1925)
**Regazzonni 2000**[39]	Internal Medicine Area	Prospective, observational	61	>70 years	Not reported		Delirium on admission, on antipsychotic medication, language barrier, severe hearing impairment, transferred from other service or hospital, incomplete follow-up	No consent mentioned	-	21.3% (13)	21.3%
**Zanocchi 1998**[40c]	Geriatric ward inpatients	Retrospective	536 for incidence<br><br>585 for occurrenc e	Range 55-86<br><br>Mean<br><br>Delirium= 79.37<br><br>No delirium= 76.52	Delirium= 13% (17/130)<br>No delirium= 4% (18/455)<br><br>No diagnosis of dementia, “chronic cognitive impairment used”		N/A	No consent mentioned	8.4% (49/585)	15% (81/536)	22% (130/585)

Author	Setting	Study Design	Sample Size	Age (years)	Dementia prevalence	Inclusion	Exclusion	Consent process	Prevalence	Incidence	Occurrence
**O'Keeffe 1996[41c]**	Acute geriatric unit	Prospective	225	Age inclusion not stated<br><br>Mean 82	Only in patients who did not have prevalent delirium (23%, 42/184)	Consecutive admission	Elective admission, rehabilitation or respite care patients, aphasic, deaf expected LOS <48 h, not assessed within 48h of admission	No consent mentioned	18% (41/225)	29% (53/184)	42% (94/225)
**Cole 1994[42]**	Medical department (excluding primary diagnosis of cerebrovascular accident)	Randomised Controlled Trial	484	≥75 years<br><br>Mean 83.3	Not given		Admission to ICU or CCU, referred to geriatrics/ oncology services, language barrier, discharged, died, multiple reasons	Assent.<br>(n=2 refused)	18% (88)	N/A	18% (88)
**Braekhus 1994[43]**	Medical department in a medium Norwegian Hospital<br>(11% of patient population is >75)	Observational study	58	>75 years<br><br>Mean 83.1	8.6% (5/58)		Delirium/acute confusion on admission	No consent mentioned (but described in the text that n=1 considered for inclusion refused).	N/A	N/A	24% (14)
**Kolbeinsson 1993[44]**	Acute medical patients	Prospective	272	≥70<br><br>Mean 80.7	18% (50/272)		Unable to assess due to severe illness; elective or overnight observation admission	No consent mentioned	14% (37)	N/A	14% (37)
**Gaudet 1993[45 c]**	Acute geriatric medicine	Prospective	487 for occurrence<br><br>466 for incidence	≥60<br><br>Mean 84.5<br><br>Range 60-105	44% (only in delirium cases)	Consecutive admissions over 5 months	Unclear	No consent mentioned	6% (28/487)	5% (24/466)	11% (52/487)
**Jitapunkul[46] 1992**	Acute geriatric ward	Prospective	184	≥60<br><br>Mean 81.7	18% (33)	Consecutive admission	Rehabilitation or respite care patients	No consent mentioned	N/A	N/A	22% (40)
**Johnson 1990[47 c]**	Internal Medicine Unit	Prospective	235	≥70<br><br>Mean 77	52% (only in delirium cases)	Consecutive admission; between Sunday afternoon and Friday evening	Transferred from another service, terminal care, planned short stay e.g., chemotherapy, transfusion, diagnostic study	Consent required.<br>(n=117 no consent)	16% (38)	4.3% (10)<br><br>(Delirium after first hospital day)	20% (48)
**Francis 1990[48 c]**	General Medical Ward	Prospective	229	≥70	Severe dementia excluded	Consecutive admission; from community	Nursing home resident, terminal care, metastatic cancer, overnight admission for invasive procedure, current psychiatric treatment, blind, deaf, aphasic, non-English speaking, severe dementia	Consent required.<br>(n=22 refused, n=11 unable to locate within 48h of admission, n=1 died before evaluation)	16% (36/229)	7% (14/193)	22% (50/229)

Author	Setting	Study Design	Sample Size	Age (years)	Dementia prevalence	Inclusion	Exclusion	Consent process	Prevalence	Incidence	Occurrence
**Rockwood 1989**[49] [c]	General Medical Ward	Prospective	80	≥65<br><br>Mean 76.8	30%, 6/20 (only in delirium cases)	Consecutive admission	Admission to CCU or ICU	No consent mentioned	16% (13)	11% (9)<br><br>Note: 2 also had prevalent delirium	25% (20)
**Cameron 1987**[50] [a,b,c]	Acute Medical Ward	Prospective	133	Likely all age (range 32-97)<br><br>Mean of delirium group= 68.8	20%, 4/20 (only in delirium cases)	Consecutive admission	Substance abuse; transferred from other services	No consent mentioned	11.3% (15/133)	4.2% (5/118)	15% (20/133)
**Anthony 1982**[51] [a,b]	General Medical Ward	Prospective	97	All age (≥20)<br><br>Mean not given	14% (14)	Consecutive admission	Non-consenting; Discharge before protocol completed	Consent required. (n=2 no consent)	10% (10)<br><br>(within 24 hours of admission)	N/A	10% (10)

[a]Studies including adult of all age (n=7, 21%). Note: Of these, four studies were from specialist settings.

[b]Studies including adult of all age from general medical ward/non-specialised ward (n=3). Note: Only two studies[50,51] in addition to our previous study[27], were from acute general medical services and both were conducted some decades ago (1982 and 1987) reporting overall delirium occurrence of 10% and 15% (prevalence (11%) and incidence (4%) in one study) without age-specific estimates.

[c]Studies reporting both prevalent and incident delirium (n=14; n=12 from non-specialised ward, n=2 from specialised ward)

Supplementary Table 2. Selected studies (of acute general (internal) medicine or geriatric wards) included in recent systematic review aimed at identifying delirium risk factors and from updated literature search.

Author	Setting	Study Design	Sample Size	Age (years)	Inclusion	Exclusion	Consent process
**Ajmera 2024**[52]	Geriatric Ward	Prospective observational	200<br><br>Delirium =40	≥60 years<br><br>Mean = 73.1 + 8.83	Consecutive admission	Hospital stay <24h, history of psychiatric illness, alcohol withdrawal delirium	Written informed consent from the patient/immediate caregiver.<br>(n=1 refused consent)
**Al Farsi 2023**[53,54]	Acute Medical Ward	Prospective cohort	327<br><br>Delirium =181	≥65<br><br>Median (IQR)= 71 (66–78)		Admission to ICU, coronary care unit, haematology and oncology wards, language barriers or aphasia	Informed consent from patients or next of kin
**Dolan 2023**[54]	General medical wards	Prospective cohort study	198<br><br>Delirium =58	≥70<br><br>Mean= 80.6/6.81	Still inpatient within 72 hours of admission	Severe aphasia, intubated, severe sensory problems, terminally ill, non-English speaking, ICU admission	Consent/assent required.<br>(n=24 declined, n=5 no assent)
**Omuojine 2023**[55]	Medical wards admission from the outpatient clinic or A&E	Prospective observational	483<br><br>Delirium=260 (Factors were determined for participants with delirium on the first day of hospital admission, n=250)	≥ 60<br><br>Median= 70 (range 60-98)	Consecutive	Deafness, non-English speaking or non-Akan-Twi (local language) speaking, discharged or died before enrolment, suspected or confirmed COVID-19	Consent from patients and/or spouses or adult children<br>(n=113 no consent)
**Song 2022**[12,56] **(Same cohort as Zhao 2021 with study period of March 2016-July 2017, but different factors assessed)**	Department of Geriatrics-Internal medical patients	Prospective	**Stress hyperglycaemia** 487<br><br>Delirium =50	≥ 70<br><br>Mean= 83.0 (5.9)	LOS ≥3 days, HbA1c value available	Delirium on admission, communication difficulty (e.g. severe deafness/ severe dementia), history of psychiatric illness, terminal illness, incomplete data	Consent required. No mention about proxy.
			**MRI-visible perivascular spaces** 114<br><br>Delirium =20	≥ 70<br>Mean= 84.3 ± 4.8 years	LOS ≥3 days, MRI imaging available with adequate quality	Delirium on admission, communication difficulty (e.g. severe deafness/ severe dementia), terminal illness, incomplete data	Consent required. No mention about proxy.
**Zhang 2022** [57] (some overlap with Song 2022 and Zhao 2021, but limited to study period of June 2016-May 2017)	Geriatric Department	Prospective study	637<br><br>Delirium=109	≥ 80	LOS >2 days	Delirium on admission, communication difficulty (e.g. severe deafness/ severe dementia), history of psychiatric illness, terminal illness	Consent required. No mention about proxy.<br><br>(n=84 declined)
**Adamis 2020**[10]	Elderly Medical Wards	Prospective, longitudinal, observational	198<br><br>Delirium=40	≥70 years<br><br>Mean= 80.6 + 6.81	Consecutive admission<br><br>Assessed for eligibility and recruited within 72 hours of admission	Readmission of existing participants, intubated, aphasia, terminal illness, non-English speaking	Informed consent required.

Author	Setting	Study Design	Sample Size	Age (years)	Inclusion	Exclusion	Consent process
**Blandfort 2020**[58] (some overlap with study below by Blandfort 2020)	Geriatric Department	Prospective	306<br><br>Delirium=58	Median (IQR)<br>Delirium= 88 (83-93)<br><br>No delirium= 86 (82-89)	Consecutive admission to acute department	No CAM assessment, somnolent/ dying, communication difficulty, non-Danish speaking, readmission, staffing holiday	Consent required to contact family<br>(n=4 refused, n=11 no relatives)
**Blandfort 2020**[9]	Geriatric department	Prospective observational	1014<br><br>Old ward = 461<br>New ward= 553<br><br>Delirium=105	≥ 75<br><br>Median (IQR)<br>Old ward= 87 (82-91)<br>New ward= 86 (81-90)	Consecutive	No CAM assessment, somnolent/moribund, communication difficulty, non-Danish speaker, readmissions	Consent not required
**Du Plooy 2020**[8]	General Medical Wards	Prospective cohort study	808<br><br>Delirium=99	≥18<br><br>Median (IQR)= 51 (IQR) 36-65	Random selection of 10 patients from daily weekday acute medical intake<br><br>First acute admission during study period	Unavailable, died, aphasia, GCS <12	Consent required (n=35 refused).<br>No mention of proxy.
**Rigor 2020**[59]	Internal medicine ward	Cohort study	198<br><br>Delirium=56	≥65<br><br>Mean=79.9/7.5		Not reported	No consent mentioned
**Zhao 2021**[60]	Geriatric ward	Prospective cohort	740	≥70 years<br><br>Mean= 84	Anticipated length of stay >2 days	Prevalent delirium; <6 months life expectancy; severe dementia/ severe deafness limiting communication; missing data	Yes
**Chouet 2020**[61]	Acute geriatric care unit	Case control study	240	≥70<br><br>Mean= 84.8	CAM +ve matched with CAM -ve	N/A	Not mentioned
**Du Plooy 2020**[8]	General medical inpatient service	Prospective cohort (random selection)	808	≥18 years<br><br>Median= 51	Acute medical admission	GCS < 12/15; not direct admission into general medical ward; communication difficulties; died before assessment; missing data; died before assessment	Consent required<br><br>(n=35 refused)
**Zrour 2020**[62]	Mixed medical, surgical or ICU	Prospective	230	≥65 years<br><br>Delirium=75<br>No delirium= 79	Convenient sampling	Unconscious, <1 day hospitalisation	Informed consent or proxy from first-degree family member
**Feast 2018**[63]	Acute medical admission unit	Exploratory secondary analysis of a longitudinal, prospective cohort study investigating pain in people with dementia	230	≥70<br><br>Mean: not given	Unplanned acute medical admission;<br>AMTS ≤7/10; people with dementia	Moribund, non-English speaking, persistent delirium without known dementia	Yes<br><br>Informed consent or by consultee
**Yam 2018**[20]	Acute General Medical Ward	Prospective Cohort	575	≥65 years<br><br>Mean 80.8	Admissions to general medical wards.	Direct admissions to ICU/ CCU/ acute stroke unit; coma/ persistent vegetative state; severe aphasia; clinically unstable/too unwell; previously recruited; non-Chinese ethnicity	Informed consent or from informant<br><br>(n=26 refused, but none had delirium)

Author	Setting	Study Design	Sample Size	Age (years)	Inclusion	Exclusion	Consent process
**Lewis 2017**[64]	Acute medical wards	Prospective	494	≥60<br><br>Median (IQR)= 75 (60-104)	Consecutive admission	Died, discharged, transferred out of the medical wards before enrolment, missing data	Consent or assent required
**O'Regan 2018**[65]	Medical admission from ED	Prospective observational (non-consecutive, but recruitment days pre-defined)	191	≥70<br><br>Mean=80	Eligibility screened ≤36 h of ED presentation (usually within 24 h)	Prevalent delirium, too unwell, ICU admission, coma or severe communication difficulty, discharged within 3 days without incident delirium, required	Yes<br>(n=19 refused; n=36 withdrew)
**Moorey 2016**[66]	Medical admission unit	Case control study	251	≥70	unplanned admission	severe sensory impairment; non-English speaking; moribund	Yes for cases<br><br>(Consultee declined/unavailable n=59)
**Egberts 2015**[67]	Internal Medicine and Geriatrics wards	Prospective	86	≥65 years		Lewy body dementia, Parkinson's disease, neuroleptic malignant syndrome, tardive dyskinesia, on psychiatric medications except haloperidol and benzodiazepine, aphasic, insufficient understanding of the Dutch language; Mini-Mental State Examination (MMSE) score <10/30	Yes
**Pendlebury 2015**[27] [a]	Acute general medicine	Prospective observational cohort	308	≥65<br><br>Median age= 82	Consecutive admission	None	No
**Fortini 2014**[68]	Two internal medicine wards from two site	Prospective observational	560	≥65 years<br><br>Mean= 80.35	Consecutive admission	Prevalent delirium	Yes
**Hein 2014**[69]	Acute geriatric ward	Prospective observational cohort	410	≥65 years<br><br>Men= 84.8<br><br>Women= 86.2	Taking >5 long-term drugs vs <6 drugs	Head injury/ stroke	No mention
**Carrasco 2014**[70]	Medical unit	Prospective cohort	Development n=542<br><br>Validation n=85	Aged ≥ 65 years<br><br>Mean= 78	Admitted in last 48 hours	Aphasic, coma, language barriers; unable to participate in cognitive test	Yes
**Joosten 2014**[71]	Acute geriatric ward	Prospective, consecutive	220	Aged ≥ 70 years<br><br>Only delirium with frailty vs non-frail		Terminally ill; non-Dutch speaking; communication difficulties; severe hearing/ visual problems; infection-related isolation; very poor health; readmission during the study period; discharged/ death ≤24 hours after admission; incomplete frailty data	Yes<br>(n=80 declined; n=27 dropped out)
**Ritchie 2014**[72]	Acute medical admissions unit	Longitudinal cohort	710	≥70<br><br>Mean 83.05	Unplanned acute admission for >48 hours	Inadequate English to complete cognitive assessment, discharged before assessment	Yes<br><br>(n=30 refused assessment)

Author	Setting	Study Design	Sample Size	Age (years)	Inclusion	Exclusion	Consent process
**Douglas 2013**[73]	Medical inpatients (medicine, cardiology, or neurology services)	Prospective cohort	Derivation cohort n=209<br><br>Validation cohort n=165	≥ 50 years<br><br>Derivation= 68.0<br><br>Validation= 70.7	non-ICU; admitted from the emergency department	Delirium on admission, admitted for alcohol withdrawal or comfort care; aphasic; non-English speaking	Yes
**Martinez 2012**[74 a]	Internal medicine wards	Prospective cohort	397	≥18 years<br><br>Mean= 75.9	Consecutive	No exclusion criteria	Yes.<br>All gave consent
**Srinonprasert 2011**[75]	General medical wards	Prospective observational study	225	≥70<br><br>Mean age= 78	Consecutive admission	Endotracheal intubated, aphasic, coma, uncooperative.	Yes<br><br>Patient and proxy
**Franco 2010**[76]	Internal medicine wards (Geriatric patients)	Nested case control	291	≥60 years<br><br>Mean= 74.4	Consecutive	Prevalent delirium; coma/stupor; died/ transferred to ICU or surgery before discharge or delirium diagnosis	Yes
**van Munster 2010**[77]	Department of Medicine	Prospective	720<br>(605 medical)	≥65	Consecutive admission	Non-Dutch or non-English speaking;<br>Missing CAM score/ genotyping for all SNPs	Yes<br>(n=399 no blood sample due to refusal/ logistic reason)
**van Munster 2010**[78]	Department of Medicine	Prospective cohort	412	≥65<br><br>Delirium= 81.6<br>No delirium= 76.6	Acute admission	Non- Dutch or non-English speaking, ward stay ≤ 48 h	Yes<br><br>Patients or proxy<br>(n=634 declined consent for blood withdrawal)
**Yang 2008**[79]	General medicine service	Prospective cohort	779<br><br>(Cohort 1= 404<br><br>Cohort 2=375)	≥70<br><br>Cohort 1= 78.5<br><br>Cohort 2=79.8	without dementia	refrained from answering ≥1 activity questions	Yes
**de Rooij 2007**[80]	Department of Internal Medicine	Cohort	185	≥65 years	Consecutive	Non-Dutch or non-English speaking,<br>transferred from or to non-Internal Medicine ward;<br>discharged within 48 h	Yes<br><br>(n=182 no informed consent)
**Inouye 2007**[81]	General medicine units (non-ICU)	Cohort 1: Observational study<br><br>Cohort 2: Prospective cohort from usual care group in a controlled clinical trial	Development n=491<br><br>Validation cohort= 461	≥70 years<br><br>Development= 79.1<br><br>Validation= 80	Consecutive;<br><br>No delirium on admission and survived to discharge	Intubated, coma, severe aphasia, terminally ill; discharged in <48 hours; previously recruited	Yes<br>n=85 patient/ physicians declined<br><br>n=250/1169 total eligible patients/family/physicians declined
**McAvay 2007**[82]	General medicine service	Secondary analysis of a prospective cohort study	416	≥70<br><br>Mean= 80.2	Intermediate / high delirium risk, not taking antidepressants at admission	Delirium at admission; profound dementia; aphasic; intubated	Yes
**Edlund 2006**[83]	General internal medicine wards	Prospective	400	≥70<br><br>Delirious= 81.8<br>Non- delirious= 79.4	Consecutive admission	Unwillingness to participate	Yes<br><br>Oral consent from patient/ next of kin

Author	Setting	Study Design	Sample Size	Age (years)	Inclusion	Exclusion	Consent process
**Jones 2006**[84]	General Medicine Service	Cohort 1: Observational study<br><br>Cohort 2: Prospective cohort from usual care group in a controlled clinical trial	Cohort 1= 491<br><br>Cohort 2=461	≥70 years<br><br>Study 1=79<br><br>Study 2=80	No delirium at baseline No delirium at baseline	Cohort 1:<br>Intubated,<br>coma, severe aphasia, terminally ill; discharged within <48 hours; previously recruited<br><br>Cohort 2:<br>As per cohort 1;<br>Profound dementia, language barrier, in isolation	Yes<br><br>(n=85 patient/ physicians declined<br><br>n=250/1169 total eligible patients/family/physicians declined)
**Joosten 2006**[85]	Acute geriatric ward	Prospective	190	≥ 70<br><br>Mean= 82.7	Participation in a recent study on delirium detection by bedside nurses	Limited verbal communication; terminally ill; readmission; discharged or transferred from another ward within 48 h of admission, Red blood cell transfusion within 3 months; ≤2 CAM tests and only negative score available	Yes<br>(n=9 refused)
**Korevaar 2005**[86]	Department of Internal Medicine	Prospective cohort	306<br>(126 randomly selected)	≥65 years<br><br>Non-selected=78.1<br><br>Selected= 79.1	Consecutive	Non-Dutch or non- English speaking; transferred from or to non-Internal Medicine ward; discharged within 48h	Yes<br><br>(n=182 did not give consent)
**Wilson 2005**[87]	Acute medical wards	Prospective	100	≥75 years<br><br>Mean=84.5	Consecutive admission of patients with significant physical illness (APACHE 11 score >8)	Delirium on admission<br><br>Insulin-dependent diabetes; severe communication problem; discharged/transferred within 48 hours; required blood transfusion	Yes<br>(n=44 refused<br>n=23 unable to provide consent as in coma/ very ill)
**Villalpando-Berumen 2003**[88]	General acute care wards	Nested case-control	667	≥60<br><br>Delirium= 75.8<br>Control= 71.3	LOS ≥ 48 hours	Delirium on arrival, sedated, intubated, aphasia	No mention
**Inouye 1996**[89]	General medical wards	Prospective cohort	Development cohort n=196<br><br>Validation cohort n=312	≥70 years<br><br>Mean=78.5	Consecutive, no delirium at baseline<br><br>During weekdays	Intubated, coma, severe aphasia, terminally ill; discharged in <48 hours; previously recruited; risk factor data missing (development cohort); isolation (validation cohort);	Yes<br><br>Patients/ physicians declined:<br><br>Development=29<br><br>Validation=56
**Foy 1995**[90]	Department of Medicine	Prospective	418	≥60 years<br><br>Mean=72	Normal cognitive function-MMSE ≥ 24 within 24h of admission<br><br>(between 12:00 Sunday and 10:00 Friday)	Urgent resuscitation;<br>semi-coma/ coma;<br>admission for day procedure/ terminal care;<br>blind; aphasic,<br>non-English speaking	Verbal consent<br><br>(n=39 refused)
**Pompei 1994**[91]	Medical and surgical wards	Prospective Cohort	Derivation cohort n=432<br><br>Test cohort n=323	Derivation cohort: Aged ≥ 65 years<br><br>Test cohort: Aged ≥ 70 years		Discharged/ unavailable to researchers within first 48 h; inability to participate in the daily interviews e.g., too ill or in	Yes<br>(n=306 refused<br>n=109 unable to provide consent due to cognitive impairment)

Author	Setting	Study Design	Sample Size	Age (years)	Inclusion	Exclusion	Consent process
						isolation, non-English speaking impairment, coma, aphasic	
**Inouye 1993**[92]	General medicine ward	Prospective cohort	Development n=107<br><br>Validation n=174	≥70 years<br><br>Development= 79.3<br><br>Validation= 78.4	From emergency service<br><br>No delirium or severe dementia at baseline	Terminally ill; violent behaviour, intubated; <48 hours hospitalisation; language barrier	Yes (n=3 patient, family, or physician declined)
**Levkoff 1992**[93]	General medical and surgical wards	Prospective	325	≥65<br><br>Mean = 81.4	From the Hebrew Rehabilitation Centre for Aged or defined community of East Boston	Direct ICU or psychiatric unit admission; severe language/ hearing problems; active TB<br><br>Eligible patient but not recruited: moribund; limited staffing; research team unable to contact before discharge	Yes<br><br>(n=57 patient, family, nurse, physician refused)
**Schor 1992**[94]	General medical and surgical wards	Prospective Cohort	291	≥65<br><br>Mean = 80.5	From the Hebrew Rehabilitation Centre for Aged or defined community of East Boston	ICU admission; severe hearing problems; psychiatric illness; active tuberculosis	Yes (n=1 no proxy to give consent)

[a]Studies with no exclusion criteria and no consent required/declined (n=2)

Supplementary Table 3. Selected studies (of acute general (internal) medicine or geriatric wards) included in meta-analysis on delirium-associated mortality and from updated literature search.

Study	Setting	Country	Sample size	Delirium sample	Mean age	Consent issues	Exclusion criteria	Age range	Time period follow up	Adjustments	Comorbidity burden	Illness Severity	Frailty
**Ajmera 2024**[52]	Geriatric ward	India	200 (but 158 followed up at 1 year after discharge)- loss to f/u n=15, died during hospital stay n=27)	40	$\geq$ 60 years<br><br>Mean 73.1/8.83	Written informed consent from the patient/immediate caregiver.<br>(n=1 refused consent)	Hospital stay <24h, history of psychiatric illness, alcohol withdrawal delirium	60+	1-year	Age, sex, IQCODE-SD score, CCI, BPRS, DRS-R-98	Yes	No	No
**Omuojine 2023**[55]	Medical wards admission from the outpatient clinic or through the A&E	Ghana	483	260	Median 70 (range 60-98)	Consent from patients and/or spouses or adult children<br>(n=113 no consent)	Deafness, non-English speaking or non-Akan-Twi (local language) speaking, discharged or died before enrolment, suspected or confirmed COVID-19	60+	within 28-day/in-hospital	Age, education, marital status, occupation, multimorbidity and TEWS	Yes	Yes	No
**Cullum 2022**[95]	Acute General Medicine	New Zealand	200	67	Mean= 82.2/5.4	Not required	Unconscious, terminally ill	75+	1 year	Age, gender, ethnicity	No	No	No
**Rawle 2021**[96]	Acute Medical unit	England	577	77	Mean= 83/7.4	Verbal consent from patient or carer	No available information on admission medications	70+	2 years	Residence, Waterlow score, CCI, dementia	Yes	No	No
**Du Plooy 2020**[8]	General Medical Wards	South Africa	808	12.3%	Median (IQR)= 51 (36-65)	Consent required (n=35 refused)<br><br>No mention of proxy	Unavailable, died, aphasia, GCS <12	18+	12-month and inpatient	None	No	No	No
**Franco 2020**[7]	Internal Medicine Wards	Colombia	200	50	Mean<br><br>No delirium= 73.4/8.2<br>SSD 78.2/9.4<br>Delirium= 80.9/7.7	Consent or proxy required	Stupor/coma, severe language or hearing difficulty	60+	During admission	None	No	No	No
**Peralta-Cuervo 2020**[97]	Geriatric unit	Colombia	1599	51.03%	Mean<br><br>Delirium= 84.9/6.6<br>No delirium= 85.8/6.1	No mention of consent	Incomplete CAM (but all patient had)	75+	In-hospital	Age, sex, pressure ulcers on admission, functional impairment, malnutrition, dementia, infection and	No	No	No (Functional impairment)

Study	Setting	Country	Sample size	Delirium sample	Mean age	Consent issues	Exclusion criteria	Age range	Time period follow up	Adjustments	Comorbidity burden	Illness Severity	Frailty
										limited social network			
**Rigor 2020[59]**	Internal Medicine Ward	Portugal	198	28.3%	Mean 79.9/7.5	No mention of consent.	Not reported.	65+	12-month and in-hospital	Age, gender, Charlson Comorbidity Index, dementia, number of drugs, anticholinergic burden	Yes	No	No
**Khor 2019[98]**	Acute Medical Wards	Malaysia	161 (screened for delirium and not excluded)	43 (26.7%)	Mean= 76.5/7.77	Not mentioned but n=6 refused screening	Terminally ill, severe hearing impairment, incomplete assessment	65+	Inpatient	None	No	No	No
**Detroyer 2018[99]**	Geriatric Ward	Belgium	Pre-and post intervention study Before= 81 After=79	Control=21 (25.9%) Intervention= 17 (21.5%)	Mean Control= 83.2/5.1 Intervention= 83.8/5.6	Consent/proxy required. (n=54 declined)	Severe hearing/visual problems, very unwell or terminally ill, isolated due to infection, unable to converse, readmission, expected discharge <24 after admission	70+	12 month and in-hospital)	Baseline functional status score and gender	No	No	No (baseline functional status)
**Diwell 2018[13]**	Acute unplanned medical admission	UK	610	69	Mean=83/7	Verbal consent or verbal assent required n=23 refused	Inadequate English skills necessary to complete basic cognitive assessments, missing data, very unwell, untraceable	70+	4 years + Mortality data censored 6/10/2011 (Admitted between 4/6/2007 to 4/ 11/2007)	Age, gender, CCI, Waterlow and APACHE II	Yes	Yes	No
**Avelino-Silva 2017[100]**	Geriatric Ward	Brazil	1409	657	Mean= 80/9	Consent required. No proxy mentioned. (n=7 no consent)	Palliative care admission, incomplete data on main variables, LOS <48h, no authorisation for research use of hospital data.	60+	12-month and in-hospital	Age, sex, marital status, referring unit, functional status, nutritional status, comorbidities, polypharmacy, vital signs, GFR, urea, albumin, total leucocytes,	Yes	No (Vital signs)	No (functional status)

Study	Setting	Country	Sample size	Delirium sample	Mean age	Consent issues	Exclusion criteria	Age range	Time period follow up	Adjustments	Comorbidity burden	Illness Severity	Frailty
										and C-reactive protein			
**Dharmarajan 2017[101 a]**	General medicine service (non-intensive care)	US	469	15% (70)	Not reported.	Consent or proxy required	Terminal illness, unable to participate in interviews, LOS <48 hours	70+	90-day	Demographic characteristics, lifestyle factors, chronic health history (including ADL, IADL etc.), acute illness severity, adverse hospital exposures	Yes	Yes	No (functional status)
**Melanie Dani, 2018[102]**	Acute Medical Unit	UK	710	73	83.1	Consent or verbal assent from family carers or key nurse	Admission for <48 hours, inadequate English for cognitive assessment	70+	3 years	Age and sex, frailty	No	No	Yes
**Dimitrios Adamis, 2018[103]**	Elderly medical wards	Ireland	200	46	81.1	No consent mentioned	Terminal illness, severe aphasia, intubation, severe sensory problems, non-English speaking.	70+	One year mortality	-	No	No	No
**Carlos Jorge-Ripper, 2017[104]**	General internal medicine unit (sepsis vs no infection)	Spain	119	83	75.8	Informed consent from patients or relatives	Delirium related to alcohol withdrawal, sepsis-associated advanced multi-organ failure, palliative admission	65-90	Short-term (in-hospital) and long term (follow-up after discharge-unclear duration)	Charlson Comorbidity Index, malnutrition, global mental recovery	Yes	No	No
**Maria-Laura Muresan, 2016[105]**	Acute medical admissions	Ireland	200	46	81.13	Informed consent from patient or assent from relatives required.	Severe aphasia; intubation; severe sensory problems; non-English speaking, no assent from consultant physician	70+	1 year	Age, LOS, APACHE II, MOCA	No	Yes	No
**Francisco J. Noriega, 2015[106]**	Cardiology unit	Spain	203	35	81.6	Informed consent required.	Planned hospitalisation, terminal condition in first 24 hours, delirium on admission	75+	12-month	Age, comorbidity, and initial diagnosis, presence of major geriatric syndrome (severe functional dependence,	Yes	No	No

Study	Setting	Country	Sample size	Delirium sample	Mean age	Consent issues	Exclusion criteria	Age range	Time period follow up	Adjustments	Comorbidity burden	Illness Severity	Frailty
										CI, depression or frailty)			
**S Jean Hsieh, 2015[107]**	Non-ICU inpatient ward	USA	260	38	Ever delirious= 83<br>Never delirious=76	verbal consent/ surrogate if delirious (135/435 eligible denied consent)	Direct ICU admission, non-English speaking, unable to assess for delirium (coma, severe dementia, severe psychiatric illness), unavailable (diagnostic tests or procedures)	65+	In-hospital	Age, REMS (Rapid Emergency Medicine Score)	No	Yes	No
**Dasgupta 2014[108]**	General medicine inpatient	Canada	1235	355	82.6	Consent by patient or caregiver (n=451 refused)	Transferred to another non-medical service (e.g., ICU or surgical service) within 7 days of admission or from other inpatient units palliative admission, life expectancy <6months, severe hearing impairment, communication difficulties, non-English speaking, nursing home resident, completely dependent, enrolled in another study	70+	3 months	-	No	No	No
**Filippo Pieralli, 2014[109]**	Internal Medicine Units (with community acquired pneumonia	Italy	434	110	81.8	No consent mentioned-retrospective study of medical records.	Hospital-acquired or healthcare-associated, immunocompromised, or had antineoplastic treatment within 60 days of admission.	65+	In-hospital	COPD, male sex, CURB 65	No	Yes (pneumonia severity)	No
**Rungnirand Praditsuwan, 2013[110]**	General Medical Wards	Thailand	225	110	78	Informed consent from patient and proxy	Intubated, communication difficulty, uncooperative, transferred to other units, death within 48 h, too unwell.	70+	3-month	Age >80, malignancy, severe illness, infection, pre-renal azotemia	No<br>Only certain conditions	Yes	No
**Shanmugam Uthamalingam, 2011[111]**	Cardiology unit (hospitalised with acute decompensated heart failure	USA	883	151	79	No consent mentioned	Nursing home residence, from skilled nursing/acute rehabilitation facility, delirium due to infection or other identified cause, intubated, history of cancer 6 months, lost to follow-up	65+	90-day	Age, gender, cardiac risk factors, dementia, ADLs, iADLS, previous HF, CAD, AF, LVEF, ACE-i/ARBs, β	Yes	No	No<br>ADLs

Study	Setting	Country	Sample size	Delirium sample	Mean age	Consent issues	Exclusion criteria	Age range	Time period follow up	Adjustments	Comorbidity burden	Illness Severity	Frailty
										blockers,CCI, other blood markers etc			
**Malaz Boustani, 2010[112]**	General Medicine Service (have cognitive impairment at the time of hospital admission)	USA	424	163	Cognitive impairment documented= 79.1<br><br>Cognitive impairment undocumented 76.1	No consent mentioned	Previously enrolled in study, enrolled in another clinical study, aphasic/unresponsive during screening, non-English speaking.	65+	30-day post-hospital	Age, gender, race, Charlson comorbidity index, and SPMSQ at screening	Yes	No	No
**Gianluca Isaia, 2009[113]**	Traditional Geriatric Hospital Ward (GHW) and Geriatric Home Hospitalisation service (GHHS)	Italy	GHW= 60<br><br>GHHS=84	GHW= 10<br><br>GHHS=4	84.7	Patient/proxy (n=84 refused)	No caregiver, terminal illness, assessment not feasible (e.g., coma, severe dementia, aphasia, intubation)	75+	6-months	-	No	No	No
**Matias Gonzalez, 2009[114 a]**	General Medical Ward	Chile	542	192	78	Informed consent from patients/ surrogates	Severe aphasia, coma, and assessment not feasible	65+	3-month	Age, sex, APACHE II score, Charlson Index, Pfeffer score, and Barthel score	Yes	Yes	No<br><br>Pfeffer score
**Kannayiram Alagiakrishnan, 2009[115]**	General Internal Medicine (deemed to be at high risk of delirium)	Canada	132	20	Median<br><br>Delirium=81<br><br>No delirium=79	Informed consent from patient or proxy	Delirium on admission, CAM +ve, hospital stay <72 hours, coma on admission, alcohol/drug withdrawal or intoxication	65+	In-hospital	-	No	No	No
**Martin G. Cole, 2008[116]**	Medical/ Geriatric services (from ED)	Canada	210	115	No delirium=83.7<br><br>Delirium= 83.3	Informed consent; Assent and family member consent if delirious/ dementia	Primary diagnosis of stroke, oncology admission with terminal illness, ICU/ CMU (cardiac) admission unless transferred to a medical ward within 48 hours of admission, non-English/French speaking	65+	12 months	Age, sex, illness severity, Charlson score and dementia status	Yes	Yes	No
**J. Holden, 2008[117]**	General Medical Wards	New Zealand	216	54	Delirious= 80.9 Non-delirious= 78.5	Verbal consent required from	Unable to assess (non-English speaking, aphasic, too ill,	65+	Likely while in hospital	-	No	No	No

Study	Setting	Country	Sample size	Delirium sample	Mean age	Consent issues	Exclusion criteria	Age range	Time period follow up	Adjustments	Comorbidity burden	Illness Severity	Frailty
						patients, carers or nursing staff	coma), transferred to another service/ hospital within 48h of admission						
**Dimitrios Adamis, 2007**[118]	Elderly medical unit (Admitted to unit within 3 days of admission)	UK	164	47	84.6	Informed consent or assent	Previously enrolled in study, terminal illness, unable to assess (severe aphasia, hearing/ visual impairment)	70+	in hospital, 6 months	Age, gender, Barthel index, MMSE, Acute Physiology Score, albumin, IFN-γ, IL-6	No	Yes	Yes
**Agneta Edlund, 2006**[83]	General Internal Medicine Wards	Sweden	400	125	Delirious= 81.9<br><br>Non-delirious=79.4	Informed consent from patient or NOK	$\geq$70	70+	12 months	-	No	No	No
**Dimitrios Adamis, 2006**[119 a]	Elderly care unit	UK	94	33	82.8	Written informed consent or assent from relatives	Severe aphasia, non-English speaking, previously enrolled in the study	70+	In-hospital	Age, gender, MMSE, CAM, APS, BISEP, Katz ADL and Delirium Rating Scale	Yes<br><br>(BISEP)	Yes	No<br><br>Katz ADL
**Matias Gonzalez, 2005**[120]	Medical and traumatology	Spain	149	58	Delirium=78.47<br><br>Non-delirium=76.36	Consent from participants or their legal representative	Severe aphasia and language barriers, hospital stay <72hr, lost to follow-up	65+	3-month	Dementia, age, overall functioning level (Karnofsky Performance Status)	No	No	No<br><br>Karnofsky Performance Status
**Douglas L. Leslie, 2005**[121]	General Medical Unit	USA	919	115	80	Informed consent from patients/proxy	Assessment not feasible (severe dementia, language barrier, severe aphasia, intubation, coma, or respiratory isolation), terminal illness, hospital stay $\leq$48 hours, previously enrolled in the study	70+	12-month	Age, male sex, ADL impairment, and Charlson Comorbidity Index score	Yes	No	No<br><br>ADL
**Bonnie J. Wakefield, 2002**[122]	General medicine units	USA	117	16	73	Consent required (n=117 refused)	Unable to participate (coma, deaf, blind, mute, or aphasic), expected hospital stay <48 hours.	65+	In-hospital	-	No	No	No
**Kenneth Rockwood, 1999**[123]	General Medicine Service	Canada	203	38	79	Informed consent from patient or proxy	Cognitive impairment <3 months before death	65+	3 years	Age, sex, comorbid illness, dementia, frailty, marital status, living arrangement	Yes	No	Yes

Study	Setting	Country	Sample size	Delirium sample	Mean age	Consent issues	Exclusion criteria	Age range	Time period follow up	Adjustments	Comorbidity burden	Illness Severity	Frailty
**Jacob Feldman, 1999[124]**	Acute Geriatric Care Unit	Israel	61	11	Delirium=83.2<br><br>No delirium=80.5	No consent mentioned	Not admitted to tge unit on the day of admission, elective admission, admission for rehabilitation, aphasic, deafness, expected LOS <48 h, moribund conditions, not assessed within 48 h of admission	70+	likely while in hospital	-	No	No	No
**Shaun O' Keeffe, 1997[125 a]**	Acute Geriatric Unit	Ireland	225	94	Delirium=82<br>No delirium= 82	No consent mentioned.	Not admitted to the unit on admission day, elective admission, severe aphasia/ deafness, expected LOS <48 hours, not assessed by a study doctor within 48 hours of admission	Geriatric unit (age inclusion not specified, mean= 82 years)	6 months after discharge	Age, illness severity, burden of comorbid disease, disability score (Katz activities of daily living scale), and dementia	Yes	Yes	No<br><br>ADL
**Mark Ardern, 1993[126]**	Acute medical ward	UK	163	23	76	No consent mentioned.	Self-harm, LOS <24 hours, admission from outside the Oxfordshire Health District, interhospital transfer	65+	while in hospital	-	No	No	No
**Joseph Francis, 1992[127]**	General medical wards	USA	229	50	78	No consent mentioned (n=34 did not agree)	Transferred from other hospitals/ nursing homes, terminal illness, severe dementia, hospital stay <48 hours, aphasia, deafness, blindness, non-English speaking	70+	2 years	Cancer, ADL (Katz A/B vs dependent), cognitive impairment (Dementia Rating Scale)	No	No	No<br><br>ADL
**Rosalind Ramsay, 1991[128]**	Acute geriatric admissions (aged ≥ 75 as well as those specifically referred for rehabilitation)	UK	110	22	Not reported for patients assessed for delirium.<br><br>Consecutive admission (n=119): Median age=83 (range 72-99)	Verbal consent (none refused)	n=9 not assessed for delirium, unclear specific reason	75+	10 week	Severity of physical illness	No	Yes	No
**Kenneth Rockwood, 1989[49]**	General Medical Services	Canada	80	20	76.8	No consent mentioned	Admitted to CCU or ICU	65+	In-hospital	-	No	No	No

[a]Studies with results of mortality adjusted for comorbidity burden, illness severity and functional impairment (i.e. not frailty), n=4

References

1. Gibb K, Seeley A, Quinn T, et al. The consistent burden in published estimates of delirium occurrence in medical inpatients over four decades: a systematic review and meta-analysis study. *Age Ageing*. 2020;**49**(3):352-60.
2. Siddiqi N, House AO, Holmes JD. Occurrence and outcome of delirium in medical in-patients: a systematic literature review. *Age Ageing*. 2006;**35**(4):350-64.
3. Ormseth CH, LaHue SC, Oldham MA, Josephson SA, Whitaker E, Douglas VC. Predisposing and Precipitating Factors Associated With Delirium: A Systematic Review. *JAMA Netw Open*. 2023;**6**(1):e2249950.
4. Aung Thein MZ, Pereira JV, Nitchingham A, Caplan GA. A call to action for delirium research: Meta-analysis and regression of delirium associated mortality. *BMC Geriatr*. 2020;**20**(1):325.
5. Lagarto L, Albuquerque E, Loureiro D, et al. Arousal changes and delirium in acute medically-ill male older patients with and without dementia: a prospective study during hospitalization. *Aging Ment Health*. 2020;**24**(5):820-7.
6. Botero Urrea M, González MC, Villa García MM, et al. Validation of the delirium diagnostic tool-provisional (DDT-Pro) in geriatric medical inpatients with diagnostic permutations of the 3Ds with and without delirium. *J Psychosom Res*. 2024;**185**:111880.
7. Franco JG, Trzepacz PT, Sepúlveda E, et al. Delirium diagnostic tool-provisional (DDT-Pro) scores in delirium, subsyndromal delirium and no delirium. *Gen Hosp Psychiatry*. 2020;**67**:107-14.
8. Du Plooy N, Day C, Manning K, et al. Prevalence and outcome of delirium among acute general medical inpatients in Cape Town, South Africa. *S Afr Med J*. 2020;**110**(6):519-24.
9. Blandfort S, Gregersen M, Rahbek K, Juul S, Damsgaard EM. Single-bed rooms in a geriatric ward prevent delirium in older patients. *Aging Clin Exp Res*. 2020;**32**(1):141-7.
10. Adamis D, Coada I, Eikelenboom P, et al. Delirium, insulin-like growth factor I, growth hormone in older inpatients. *World J Psychiatry*. 2020;**10**(9):212-22.
11. Song Q, Dai M, Zhao Y, Lin T, Huang L, Yue J. Association between stress hyperglycemia ratio and delirium in older hospitalized patients: a cohort study. *BMC Geriatr*. 2022;**22**(1):277.
12. Song Q, Zhao Y, Lin T, Yue J. Perivascular spaces visible on magnetic resonance imaging predict subsequent delirium in older patients. *Front Aging Neurosci*. 2022;**14**:897802.
13. Diwell RA, Davis DH, Vickerstaff V, Sampson EL. Key components of the delirium syndrome and mortality: greater impact of acute change and disorganised thinking in a prospective cohort study. *BMC Geriatr*. 2018;**18**(1):24.
14. Pouw MA, Calf AH, Georg RR, de Rooij SE, Ter Maaten JC, van Munster BC. Diagnostic accuracy of the Dutch version of the 4AT for delirium detection in a mixed patient population and setting. *Aging Clin Exp Res*. 2023;**35**(8):1705-10.
15. Paddick SM, Gamassa E, Mwaluwinga N, et al. Preliminary evaluation of a smartphone application (DelApp) for identification of delirium in sub-Saharan Africa. *Acta Neuropsychiatr*. 2023;**Jun 22**:1-9.
16. Geriatric Medicine Research C. Increasing frailty is associated with higher prevalence and reduced recognition of delirium in older hospitalised inpatients: results of a multi-centre study. *Eur Geriatr Med*. 2023;**14**(2):325-32.
17. Franco JG, Trzepacz PT, Velásquez-Tirado JD, et al. Discriminant Performance of Dysexecutive and Frontal Release Signs for Delirium in Patients With High Dementia Prevalence: Implications for Neural Network Impairment. *J Acad Consult Liaison Psychiatry*. 2021;**62**(1):56-69.
18. Geriatric Medicine Research C. Delirium is prevalent in older hospital inpatients and associated with adverse outcomes: results of a prospective multi-centre study on World Delirium Awareness Day. *BMC Med*. 2019;**17**(1):229.
19. Bellelli G, Zambon A, Volpato S, et al. The association between delirium and sarcopenia in older adult patients admitted to acute geriatrics units: Results from the GLISTEN multicenter observational study. *Clin Nutr*. 2018;**37**(5):1498-504.
20. Yam KK, Shea YF, Chan TC, et al. Prevalence and risk factors of delirium and subsyndromal delirium in Chinese older adults. *Geriatr Gerontol Int*. 2018;**18**(12):1625-8.
21. Chan KY, Cheng LS, Mak IW, Ng SW, Yiu MG, Chu CM. Delirium is a Strong Predictor of Mortality in Patients Receiving Non-invasive Positive Pressure Ventilation. *Lung*. 2017;**195**(1):115-25.
22. Grandahl MG, Nielsen SE, Koerner EA, Schultz HH, Arnfred SM. Prevalence of delirium among patients at a cancer ward: Clinical risk factors and prediction by bedside cognitive tests. *Nord J Psychiatry*. 2016;**70**(6):413-7.
23. Jackson TA, MacLullich AM, Gladman JR, Lord JM, Sheehan B. Undiagnosed long-term cognitive impairment in acutely hospitalised older medical patients with delirium: a prospective cohort study. *Age Ageing*. 2016;**45**(4):493-9.

24. Kozak HH, Uguz F, Kilinc I, et al. Delirium in patients with acute ischemic stroke admitted to the non-intensive stroke unit: Incidence and association between clinical features and inflammatory markers. *Neurol Neurochir Pol*. 2017;**51**(1):38-44.
25. Adamis D, Rooney S, Meagher D, Mulligan O, McCarthy G. A comparison of delirium diagnosis in elderly medical inpatients using the CAM, DRS-R98, DSM-IV and DSM-5 criteria. *Int Psychogeriatr*. 2015;**27**(6):883-9.
26. Holtta E. H. LM-L, Laurila J. V. , Strandberg T. E., Tilvis R. S., Pitkala K. H. ,. Psychotic symptoms of dementia, their relationship with delirium and prognostic value. *European Geriatric Medicine*. 2015;**6**(3):257-61.
27. Pendlebury ST, Lovett NG, Smith SC, et al. Observational, longitudinal study of delirium in consecutive unselected acute medical admissions: age-specific rates and associated factors, mortality and re-admission. *BMJ Open*. 2015;**5**(11):e007808.
28. Uchida M, Okuyama T, Ito Y, et al. Prevalence, course and factors associated with delirium in elderly patients with advanced cancer: a longitudinal observational study. *Jpn J Clin Oncol*. 2015;**45**(10):934-40.
29. Bonetti F, Magon S, Gasperini B, et al. Risk factors associated to delirium in hospitalized elderly patients. *Giornale di Gerontologia*. 2012;**60**:142-8.
30. Praditsuwan R, Limmathuroskul D, Assanasen J, et al. Prevalence and incidence of delirium in Thai older patients: a study at general medical wards in Siriraj Hospital. *J Med Assoc Thai*. 2012;**95 Suppl 2**:S245-50.
31. Thomas C, Kreisel SH, Oster P, Driessen M, Arolt V, Inouye SK. Diagnosing delirium in older hospitalized adults with dementia: adapting the confusion assessment method to international classification of diseases, tenth revision, diagnostic criteria. *J Am Geriatr Soc*. 2012;**60**(8):1471-7.
32. Travers C, Byrne G, Pachana N, Klein K, Gray L. Prospective observational study of dementia and delirium in the acute hospital setting. *Intern Med J*. 2013;**43**(3):262-9.
33. Paci C, Gobbato R, Carboni T, Sanguigni S, Santone A, Coccia G. Quetiapine responsive delirium in acute stroke. *Rivista di Psichiatria*. 2008;**43**(2):101-3.
34. Sheng AZ, Shen Q, Cordato D, Zhang YY, Yin Chan DK. Delirium within three days of stroke in a cohort of elderly patients. *J Am Geriatr Soc*. 2006;**54**(8):1192-8.
35. Lundstrom M, Edlund A, Karlsson S, Brannstrom B, Bucht G, Gustafson Y. A multifactorial intervention program reduces the duration of delirium, length of hospitalization, and mortality in delirious patients. *J Am Geriatr Soc*. 2005;**53**(4):622-8.
36. Laurila JV, Pitkala KH, Strandberg TE, Tilvis RS. The impact of different diagnostic criteria on prevalence rates for delirium. *Dement Geriatr Cogn Disord*. 2003;**16**(3):156-62.
37. Laurila JV, Pitkala KH, Strandberg TE, Tilvis RS. Detection and documentation of dementia and delirium in acute geriatric wards. *Gen Hosp Psychiatry*. 2004;**26**(1):31-5.
38. Cole MG, McCusker J, Bellavance F, et al. Systematic detection and multidisciplinary care of delirium in older medical inpatients: a randomized trial. *CMAJ*. 2002;**167**(7):753-9.
39. Regazzoni CJ, Aduriz M, Recondo M. [Acute confusion syndrome in the hospitalized elderly]. *Medicina (B Aires)*. 2000;**60**(3):335-8.
40. Zanocchi M, Vallero F, Norelli L, Zaccagna B, Spada S, Fabris F. [Acute confusion in the geriatric patient]. *Recenti Prog Med*. 1998;**89**(5):229-34.
41. O'Keeffe ST, Lavan JN. Predicting delirium in elderly patients: development and validation of a risk-stratification model. *Age Ageing*. 1996;**25**(4):317-21.
42. Cole MG, Primeau FJ, Bailey RF, et al. Systematic intervention for elderly inpatients with delirium: a randomized trial. *CMAJ*. 1994;**151**(7):965-70.
43. Braekhus A, Engedal K. [Delirium (acute confusion) among elderly patients after admission to a medical department]. *Tidsskr Nor Laegeforen*. 1994;**114**(22):2613-5.
44. Kolbeinsson H, Jonsson A. Delirium and dementia in acute medical admissions of elderly patients in Iceland. *Acta Psychiatr Scand*. 1993;**87**(2):123-7.
45. Gaudet M, Pfitzenmeyer P, Tavernier-Vidal B, Lechenet M. Les états confusionnels en milieu interniste gériatrique court séjour. *Psychologie médicale*. 1993;**25**(7):611-4.
46. Jitapunkul S, Pillay I, Ebrahim S. Delirium in newly admitted elderly patients: a prospective study. *Q J Med*. 1992;**83**(300):307-14.
47. Johnson JC, Gottlieb GL, Sullivan E, et al. Using DSM-III criteria to diagnose delirium in elderly general medical patients. *J Gerontol*. 1990;**45**(3):M113-9.
48. Francis J, Martin D, Kapoor WN. A prospective study of delirium in hospitalized elderly. *JAMA*. 1990;**263**(8):1097-101.
49. Rockwood K. Acute confusion in elderly medical patients. *J Am Geriatr Soc*. 1989;**37**(2):150-4.
50. Cameron DJ, Thomas RI, Mulvihill M, Bronheim H. Delirium: a test of the Diagnostic and Statistical Manual III criteria on medical inpatients. *J Am Geriatr Soc*. 1987;**35**(11):1007-10.
51. Anthony JC, LeResche L, Niaz U, von Korff MR, Folstein MF. Limits of the 'Mini-Mental State' as a screening test for dementia and delirium among hospital patients. *Psychol Med*. 1982;**12**(2):397-408.
52. Ajmera Y, Paul K, Khan MA, et al. The evaluation of frequency and predictors of delirium and its short-term and long-term outcomes in hospitalized older adults'. *Asian J Psychiatr*. 2024;**94**:103990.
53. Al Farsi RS, Al Alawi AM, Al Huraizi AR, et al. Delirium in Medically Hospitalized Patients: Prevalence, Recognition and Risk Factors: A Prospective Cohort Study. *J Clin Med*. 2023;**12**(12).

54. Dolan C, Mohd Zubir M, Melvin V, McCarthy G, Meagher D, Adamis D. Delirium occurrence in older Irish adults admitted to an acute medical hospital: a prospective cohort study. *Ir J Psychol Med*. 2023;**40**(3):369-77.
55. Omuojine JP, Bello T, Wemakor S, et al. Risk factors and outcomes of delirium in hospitalized older Ghanaians. *Int J Geriatr Psychiatry*. 2023;**38**(4):e5912.
56. Song J, Cheng C, Sheng K, et al. Association between the reactivity of local cerebral oxygen saturation after hypo-to-hypercapnic tests and delirium after abdominal surgery in older adults: A prospective study. *Front Psychiatry*. 2022;**13**:907870.
57. Zhang M, Zhang X, Gao L, Yue J, Jiang X. Incidence, predictors and health outcomes of delirium in very old hospitalized patients: a prospective cohort study. *BMC Geriatr*. 2022;**22**(1):262.
58. Blandfort S, Gregersen M, Rahbek K, Juul S, Damsgaard EM. The short IQCODE as a predictor for delirium in hospitalized geriatric patients. *Aging Clin Exp Res*. 2020;**32**(10):1969-76.
59. Rigor J, Rueff Rato I, Ferreira PM, et al. Prehospital Anticholinergic Burden Is Associated With Delirium but Not With Mortality in a Population of Acutely Ill Medical Patients. *J Am Med Dir Assoc*. 2020;**21**(4):481-5.
60. Zhao Y, Yue J, Lei P, et al. Neutrophil-lymphocyte ratio as a predictor of delirium in older internal medicine patients: a prospective cohort study. *BMC Geriatr*. 2021;**21**(1):334.
61. Chouet J, Sacco G, Karras SN, Llewellyn DJ, Sanchez-Rodriguez D, Annweiler C. Vitamin D and Delirium in Older Adults: A Case-Control Study in Geriatric Acute Care Unit. *Front Neurol*. 2020;**11**:1034.
62. Zrour C, Haddad R, Zoghbi M, Kharsa Z, Hijazi M, Naja W. Prospective, multi-centric benchmark study assessing delirium: prevalence, incidence and its correlates in hospitalized elderly Lebanese patients. *Aging Clin Exp Res*. 2020;**32**(4):689-97.
63. Feast AR, White N, Lord K, Kupeli N, Vickerstaff V, Sampson EL. Pain and delirium in people with dementia in the acute general hospital setting. *Age Ageing*. 2018;**47**(6):841-6.
64. Lewis EG, Banks J, Paddick SM, et al. Risk Factors for Delirium in Older Medical Inpatients in Tanzania. *Dement Geriatr Cogn Disord*. 2017;**44**(3-4):160-70.
65. O'Regan NA, Fitzgerald J, Adamis D, Molloy DW, Meagher D, Timmons S. Predictors of Delirium Development in Older Medical Inpatients: Readily Identifiable Factors at Admission. *J Alzheimers Dis*. 2018;**64**(3):775-85.
66. Moorey HC, Zaidman S, Jackson TA. Delirium is not associated with anticholinergic burden or polypharmacy in older patients on admission to an acute hospital: an observational case control study. *BMC Geriatr*. 2016;**16**(1):162.
67. Egberts A, Wijnbeld EH, Fekkes D, et al. Neopterin: a potential biomarker for delirium in elderly patients. *Dement Geriatr Cogn Disord*. 2015;**39**(1-2):116-24.
68. Fortini A, Morettini A, Tavernese G, Facchini S, Tofani L, Pazzi M. Delirium in elderly patients hospitalized in internal medicine wards. *Intern Emerg Med*. 2014;**9**(4):435-41.
69. Hein C, Forgues A, Piau A, Sommet A, Vellas B, Nourhashemi F. Impact of polypharmacy on occurrence of delirium in elderly emergency patients. *J Am Med Dir Assoc*. 2014;**15**(11):850 e11-5.
70. Carrasco GM, Villarroel DL, Calderon PJ, Martinez FG, Andrade AM, Gonzalez TM. [Development and validation of a clinical predictive model for delirium in hospitalized older people]. *Rev Med Chil*. 2014;**142**(7):826-32.
71. Joosten E, Demuynck M, Detroyer E, Milisen K. Prevalence of frailty and its ability to predict in hospital delirium, falls, and 6-month mortality in hospitalized older patients. *BMC Geriatr*. 2014;**14**:1.
72. Ritchie CW, Newman TH, Leurent B, Sampson EL. The association between C-reactive protein and delirium in 710 acute elderly hospital admissions. *Int Psychogeriatr*. 2014;**26**(5):717-24.
73. Douglas VC, Hessler CS, Dhaliwal G, et al. The AWOL tool: derivation and validation of a delirium prediction rule. *J Hosp Med*. 2013;**8**(9):493-9.
74. Martinez JA, Belastegui A, Basabe I, et al. Derivation and validation of a clinical prediction rule for delirium in patients admitted to a medical ward: an observational study. *BMJ Open*. 2012;**2**(5).
75. Srinonprasert V, Pakdeewongse S, Assanasen J, et al. Risk factors for developing delirium in older patients admitted to general medical wards. *J Med Assoc Thai*. 2011;**94 Suppl 1**:S99-104.
76. Franco JG, Valencia C, Bernal C, et al. Relationship between cognitive status at admission and incident delirium in older medical inpatients. *J Neuropsychiatry Clin Neurosci*. 2010;**22**(3):329-37.
77. van Munster BC, Yazdanpanah M, Tanck MW, et al. Genetic polymorphisms in the DRD2, DRD3, and SLC6A3 gene in elderly patients with delirium. *Am J Med Genet B Neuropsychiatr Genet*. 2010;**153B**(1):38-45.
78. van Munster BC, Korevaar JC, Korse CM, Bonfrer JM, Zwinderman AH, de Rooij SE. Serum S100B in elderly patients with and without delirium. *Int J Geriatr Psychiatry*. 2010;**25**(3):234-9.

79. Yang FM, Inouye SK, Fearing MA, Kiely DK, Marcantonio ER, Jones RN. Participation in activity and risk for incident delirium. *J Am Geriatr Soc*. 2008;**56**(8):1479-84.
80. de Rooij SE, van Munster BC, Korevaar JC, Levi M. Cytokines and acute phase response in delirium. *J Psychosom Res*. 2007;**62**(5):521-5.
81. Inouye SK, Zhang Y, Jones RN, Kiely DK, Yang F, Marcantonio ER. Risk factors for delirium at discharge: development and validation of a predictive model. *Arch Intern Med*. 2007;**167**(13):1406-13.
82. McAvay GJ, Van Ness PH, Bogardus ST, Jr., et al. Depressive symptoms and the risk of incident delirium in older hospitalized adults. *J Am Geriatr Soc*. 2007;**55**(5):684-91.
83. Edlund A, Lundstrom M, Karlsson S, Brannstrom B, Bucht G, Gustafson Y. Delirium in older patients admitted to general internal medicine. *J Geriatr Psychiatry Neurol*. 2006;**19**(2):83-90.
84. Jones RN, Yang FM, Zhang Y, Kiely DK, Marcantonio ER, Inouye SK. Does educational attainment contribute to risk for delirium? A potential role for cognitive reserve. *J Gerontol A Biol Sci Med Sci*. 2006;**61**(12):1307-11.
85. Joosten E, Lemiengre J, Nelis T, Verbeke G, Milisen K. Is anaemia a risk factor for delirium in an acute geriatric population? *Gerontology*. 2006;**52**(6):382-5.
86. Korevaar JC, van Munster BC, de Rooij SE. Risk factors for delirium in acutely admitted elderly patients: a prospective cohort study. *BMC Geriatr*. 2005;**5**:6.
87. Wilson K, Broadhurst C, Diver M, Jackson M, Mottram P. Plasma insulin growth factor-1 and incident delirium in older people. *Int J Geriatr Psychiatry*. 2005;**20**(2):154-9.
88. Villalpando-Berumen JM, Pineda-Colorado AM, Palacios P, Reyes-Guerrero J, Villa AR, Gutierrez-Robledo LM. Incidence of delirium, risk factors, and long-term survival of elderly patients hospitalized in a medical specialty teaching hospital in Mexico City. *Int Psychogeriatr*. 2003;**15**(4):325-36.
89. Inouye SK, Charpentier PA. Precipitating factors for delirium in hospitalized elderly persons. Predictive model and interrelationship with baseline vulnerability. *JAMA*. 1996;**275**(11):852-7.
90. Foy A, O'Connell D, Henry D, Kelly J, Cocking S, Halliday J. Benzodiazepine use as a cause of cognitive impairment in elderly hospital inpatients. *J Gerontol A Biol Sci Med Sci*. 1995;**50**(2):M99-106.
91. Pompei P, Foreman M, Rudberg MA, Inouye SK, Braund V, Cassel CK. Delirium in hospitalized older persons: outcomes and predictors. *J Am Geriatr Soc*. 1994;**42**(8):809-15.
92. Inouye SK, Viscoli CM, Horwitz RI, Hurst LD, Tinetti ME. A predictive model for delirium in hospitalized elderly medical patients based on admission characteristics. *Ann Intern Med*. 1993;**119**(6):474-81.
93. Levkoff SE, Evans DA, Liptzin B, et al. Delirium. The occurrence and persistence of symptoms among elderly hospitalized patients. *Arch Intern Med*. 1992;**152**(2):334-40.
94. Schor JD, Levkoff SE, Lipsitz LA, et al. Risk factors for delirium in hospitalized elderly. *JAMA*. 1992;**267**(6):827-31.
95. Cullum S, Kubba Y, Varghese C, Coomarasamy C, Hopkins J. The evidence for introducing case-finding for delirium and dementia in older medical inpatients in a New Zealand hospital. *Australas Psychiatry*. 2022;**30**(3):303-7.
96. Rawle MJ, McCue L, Sampson EL, Davis D, Vickerstaff V. Anticholinergic Burden Does Not Influence Delirium Subtype or the Delirium-Mortality Association in Hospitalized Older Adults: Results from a Prospective Cohort Study. *Drugs Aging*. 2021;**38**(3):233-42.
97. Peralta-Cuervo AF, Garcia-Cifuentes E, Castellanos-Perilla N, Chavarro-Carvajal DA, Venegas-Sanabria LC, Cano-Gutierrez CA. Delirium prevalence in a Colombian hospital, association with geriatric syndromes and complications during hospitalization. *Rev Esp Geriatr Gerontol*. 2021;**56**(2):69-74.
98. Khor HM, Ong HC, Tan BK, et al. Assessment of Delirium Using the Confusion Assessment Method in Older Adult Inpatients in Malaysia. *Geriatrics (Basel)*. 2019;**4**(3).
99. Detroyer E, Dobbels F, Teodorczuk A, et al. Effect of an interactive E-learning tool for delirium on patient and nursing outcomes in a geriatric hospital setting: findings of a before-after study. *BMC Geriatr*. 2018;**18**(1):19.
100. Avelino-Silva TJ, Campora F, Curiati JA, Jacob-Filho W. Association between delirium superimposed on dementia and mortality in hospitalized older adults: A prospective cohort study. *PLoS Med*. 2017;**14**(3):e1002264.
101. Dharmarajan K, Swami S, Gou RY, Jones RN, Inouye SK. Pathway from Delirium to Death: Potential In-Hospital Mediators of Excess Mortality. *J Am Geriatr Soc*. 2017;**65**(5):1026-33.
102. Dani M, Owen LH, Jackson TA, Rockwood K, Sampson EL, Davis D. Delirium, Frailty, and Mortality: Interactions in a Prospective Study of Hospitalized Older People. *J Gerontol A Biol Sci Med Sci*. 2018;**73**(3):415-8.
103. Adamis D, Meagher D, Rooney S, Mulligan O, McCarthy G. A comparison of outcomes according to different diagnostic systems for delirium (DSM-5, DSM-IV, CAM, and DRS-R98). *Int Psychogeriatr*. 2018;**30**(4):591-6.
104. Jorge-Ripper C, Aleman MR, Ros R, et al. Prognostic value of acute delirium recovery in older adults. *Geriatr Gerontol Int*. 2017;**17**(8):1161-7.
105. Muresan ML, Adamis D, Murray O, O'Mahony E, McCarthy G. Delirium, how does it end? Mortality as an outcome in older medical inpatients. *Int J Geriatr Psychiatry*. 2016;**31**(4):349-54.

106. Noriega FJ, Vidan MT, Sanchez E, et al. Incidence and impact of delirium on clinical and functional outcomes in older patients hospitalized for acute cardiac diseases. *Am Heart J*. 2015;**170**(5):938-44.
107. Hsieh SJ, Madahar P, Hope AA, Zapata J, Gong MN. Clinical deterioration in older adults with delirium during early hospitalisation: a prospective cohort study. *BMJ Open*. 2015;**5**(9):e007496.
108. Dasgupta M, Brymer C. Prognosis of delirium in hospitalized elderly: worse than we thought. *Int J Geriatr Psychiatry*. 2014;**29**(5):497-505.
109. Pieralli F, Vannucchi V, Mancini A, et al. Delirium is a predictor of in-hospital mortality in elderly patients with community acquired pneumonia. *Intern Emerg Med*. 2014;**9**(2):195-200.
110. Praditsuwan R, Sirisuwat A, Assanasen J, et al. Short-term clinical outcomes in delirious older patients: a study at general medical wards in a university hospital in Thailand. *Geriatr Gerontol Int*. 2013;**13**(4):972-7.
111. Uthamalingam S, Gurm GS, Daley M, Flynn J, Capodilupo R. Usefulness of acute delirium as a predictor of adverse outcomes in patients >65 years of age with acute decompensated heart failure. *Am J Cardiol*. 2011;**108**(3):402-8.
112. Boustani M, Baker MS, Campbell N, et al. Impact and recognition of cognitive impairment among hospitalized elders. *J Hosp Med*. 2010;**5**(2):69-75.
113. Isaia G, Astengo MA, Tibaldi V, et al. Delirium in elderly home-treated patients: a prospective study with 6-month follow-up. *Age (Dordr)*. 2009;**31**(2):109-17.
114. Gonzalez M, Martinez G, Calderon J, et al. Impact of delirium on short-term mortality in elderly inpatients: a prospective cohort study. *Psychosomatics*. 2009;**50**(3):234-8.
115. Alagiakrishnan K, Marrie T, Rolfson D, et al. Gaps in patient care practices to prevent hospital-acquired delirium. *Can Fam Physician*. 2009;**55**(10):e41-6.
116. Cole MG, You Y, McCusker J, Ciampi A, Belzile E. The 6 and 12 month outcomes of older medical inpatients who recover from delirium. *Int J Geriatr Psychiatry*. 2008;**23**(3):301-7.
117. Holden J, Jayathissa S, Young G. Delirium among elderly general medical patients in a New Zealand hospital. *Intern Med J*. 2008;**38**(8):629-34.
118. Adamis D, Treloar A, Darwiche FZ, Gregson N, Macdonald AJ, Martin FC. Associations of delirium with in-hospital and in 6-months mortality in elderly medical inpatients. *Age Ageing*. 2007;**36**(6):644-9.
119. Adamis D, Treloar A, Martin FC, Macdonald AJ. Recovery and outcome of delirium in elderly medical inpatients. *Arch Gerontol Geriatr*. 2006;**43**(2):289-98.
120. Gonzalez M dPJ, Valdes M, Matrai S, Peri JM, Fuente E. Delirium: a predictor of mortality in the elderly. *Eur J Psychiatr*. 2005;**19**(3):165–71.
121. Leslie DL, Zhang Y, Holford TR, Bogardus ST, Leo-Summers LS, Inouye SK. Premature death associated with delirium at 1-year follow-up. *Arch Intern Med*. 2005;**165**(14):1657-62.
122. Wakefield BJ. Behaviors and outcomes of acute confusion in hospitalized patients. *Appl Nurs Res*. 2002;**15**(4):209-16.
123. Rockwood K, Cosway S, Carver D, Jarrett P, Stadnyk K, Fisk J. The risk of dementia and death after delirium. *Age Ageing*. 1999;**28**(6):551-6.
124. Feldman J, Yaretzky A, Kaizimov N, Alterman P, Vigder C. Delirium in an acute geriatric unit: clinical aspects. *Arch Gerontol Geriatr*. 1999;**28**(1):37-44.
125. O'Keeffe S, Lavan J. The prognostic significance of delirium in older hospital patients. *J Am Geriatr Soc*. 1997;**45**(2):174-8.
126. Ardern M, Mayou R, Feldman E, Hawton K. Cognitive impairment in the elderly medically ill: How often is it missed? *International Journal of Geriatric Psychiatry*. 1993;**8**(11):929-37.
127. Francis J, Kapoor WN. Prognosis after hospital discharge of older medical patients with delirium. *J Am Geriatr Soc*. 1992;**40**(6):601-6.
128. Ramsay R, Wright, P., Katz, A., Bielawska, C., & Katona, C. . The detection of psychiatric morbidity and its effects on outcome in acute elderly medical admissions. *International Journal of Geriatric Psychiatry*. 1991;**6(12)**:861–6.

Supplementary Methods

Description of the acute medical admissions service at the John Radcliffe Hospital

In the U.K., all acute emergency care is provided through the National Health Service (NHS). The Oxford University Hospitals NHS Foundation Trust (OUHFT) consists of four hospitals (John Radcliffe Hospital, Nuffield Orthopaedic Centre and Churchill Hospital in Oxford, and the Horton General Hospital in Banbury) providing all acute hospital care in Oxfordshire, UK with a catchment area of ~800,000 people. The John Radcliffe Hospital provides the acute general medicine admissions service for the entire Oxfordshire region with the exception of Banbury and the northernmost part of the region. The Oxfordshire population is broadly in line with the population of England as a whole in terms of age distribution and ethnic mix (~86% of the background Oxfordshire population are White based on the 2021 Census Data)[1] and all levels of deprivation are represented although the region is less deprived overall.

Acute general medicine at the John Radcliffe hospital is run as a firm-based system with each firm participating in the acute medical take according to the rota. Each firm is led by a Consultant Physician (Attending Physician or Hospitalist) and includes senior and junior resident doctors (previously known as medical registrar, senior house officer, and house officer). The firm admits consecutive acute general medical patients over a specified time window on set days over an eight-week period (see example of rota below for a given firm's medical take rota). Our cohorts are therefore representative of acute general medicine admissions to the Oxfordshire region as a whole. Both STP/SCS are Consultant Physicians and Geriatricians with >15 years' experience in leading an acute medicine (hospitalist) firm.

Patients requiring medical admission are referred directly from the Emergency Department or from the General Practitioner or paramedic to the acute medical on-take team. There is no separate admissions path for geriatric medicine patients although the small number of patients who require highly specialised care may be admitted directly to the relevant specialist unit. The majority of patients admitted under an acute medicine firm remain under the care of the same medical team and are reviewed daily on weekdays and as required at weekends by the weekend covering team where the firm are not on weekend duties.

It has been mandatory since 2013 for all OUHFT patients aged ≥65 years (≥70 years until 2024) with unplanned (emergency) admission to acute medicine to undergo cognitive screening (see below) usually administered on admission as part of the clerking process by resident doctors who are trained in the use of the cognitive screen. Since 2015, the cognitive screen has been implemented into the electronic patient record (EPR) and is mandatory for all patients with unplanned admission irrespective of specialty.

References

1. Office for National Statistics. Regional ethnic diversity. 2022. https://www.ethnicity-facts-figures.service.gov.uk/uk-population-by-ethnicity/national-and-regional-populations/regional-ethnic-diversity/latest/ (accessed 18th May 2025).

Cognitive screen and delirium diagnosis

In 2010, STP designed a cognitive screen (including the MMSE and CAM for delirium) to be administered on admission by resident doctors to all patients aged ≥70 years. In a feasibility pilot in 2010, the screen was administered to all patients admitted to acute general medicine under the care of STP/SCS by resident doctors who were trained in the use of the MMSE and CAM by STP. For patients who were untestable owing to being too unwell, too confused or for other reasons, a multichoice list was used to record the reasons for untestability. Following this pilot, the screen was changed to the 10-point Abbreviated Mental Test Score (AMTS) and CAM since the MMSE was too long to administer as part of standard care at admission. The revised screen (AMTS+CAM) was thereafter administered to all patients admitted to acute general medicine under the care of STP/SCS by the resident doctors working on the STP/SCS team.

The AMTS provides a measure of the patient's cognitive function *whether or not they have delirium*. Many patients are not delirious but may nevertheless be impaired because of dementia (which may be undiagnosed) or mild or transient cognitive impairments. In addition, documentation of a normal AMTS may be helpful in establishing the patient's baseline (accepting that the AMTS is specific but not overly sensitive for cognitive impairments). The AMTS is not itself diagnostic of delirium but it does inform the application of the CAM. The AMTS allows identification of a cognitive deficit and its severity, but the next step is to determine whether this is caused by delirium, dementia, delirium superimposed on dementia or something else. OUHFT training around delirium highlights the need to use information from the clinical assessment process to decide whether the CAM screen is positive and then whether a clinical diagnosis of delirium can be made according to the DSM-IV (now DSM-5) criteria.

In the current study, all patients admitted under the care of STP/SCS, were reviewed as soon as possible (<12 hours and usually <4 hours) after admission by STP or SCS and at least every 48 hours thereafter until discharge (or death) during daily team ward rounds or ad hoc according to clinical need *irrespective of on-admission CAM status* ie the patients were all seen by STP/SCSC as part of the patients' standard acute medical care. Diagnosis of delirium was made according to the DSM-IV criteria by STP after review of all available information including the on-admission screening results, discussions with the team resident doctors and the wider multidisciplinary team, and information from medical records and informants including family members.

Example of a weekly acute medicine firm rota

	**Monday**	**Tuesday**	**Wednesday**	**Thursday**	**Friday**	**Saturday**	**Sunday**
**Week 1**							Night
**Week 2**		Night			Evening	Evening + Night	
**Week 3**				Evening			
**Week 4**	Night		Day			Day	Evening
**Week 5**		Day		Night			
**Week 6**	Evening			Day			
**Week 7**	Day		Evening		Night		Day
**Week 8**		Evening			Day		
**Week 9**			Night				
**Note: Day take: 08:00-16:00, Evening take: 16:00-21:00, Night take: 21:00-08:00**							

Supplementary Results

Supplementary Figure 1. Venn diagram displaying numbers with prevalent delirium, incident delirium and both across the entire adult age range

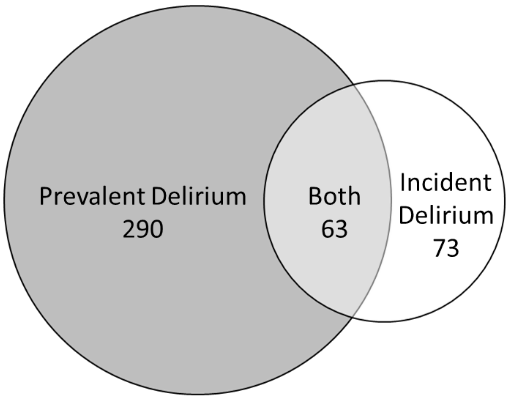

Supplementary Figure 2. Age-specific a) delirium occurrence, b) delirium occurrence by subtype, c) dementia prevalence, and d) delirium occurrence by dementia status.

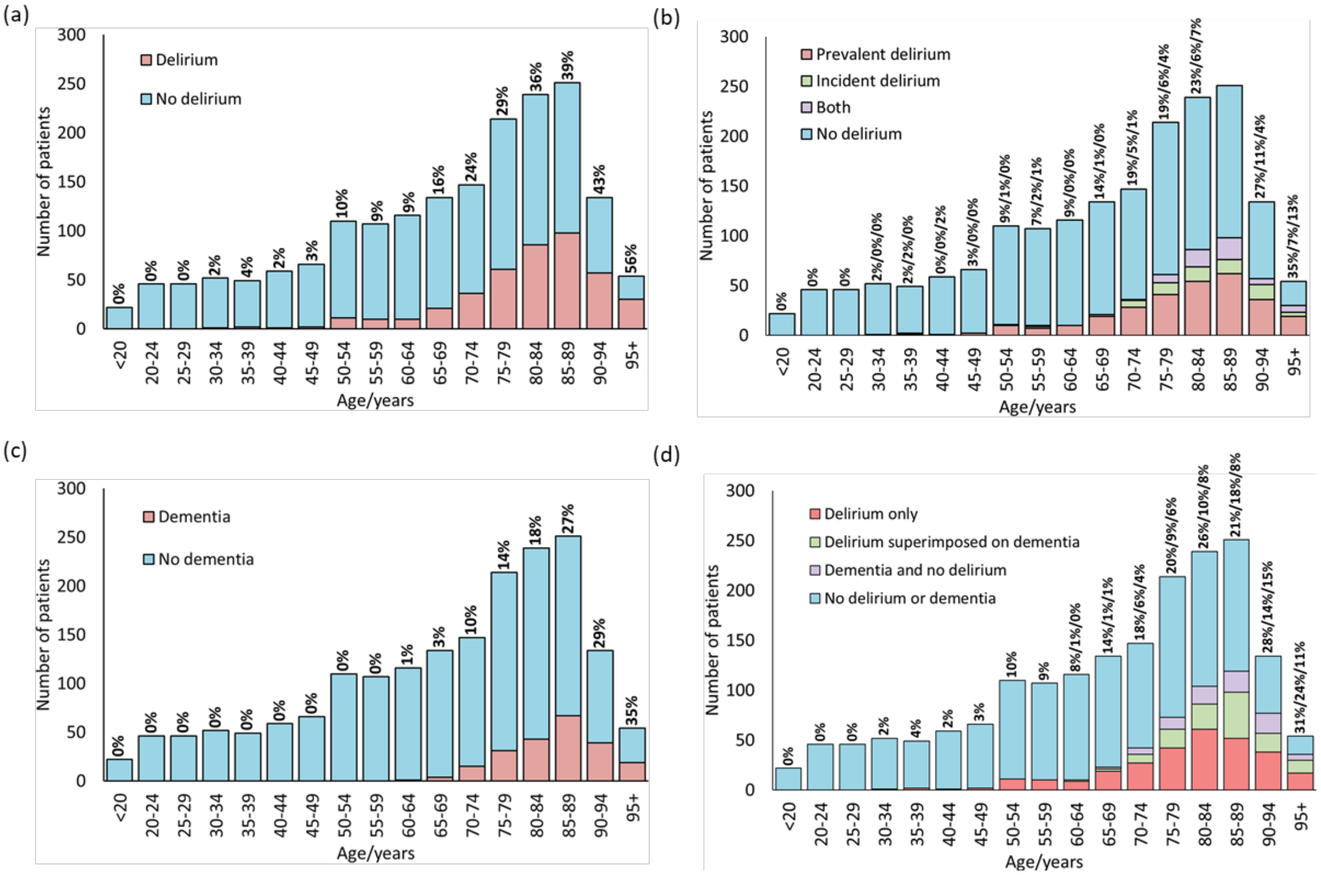

Note: Percentage displayed in figure is rounded to nearest whole number.

Supplementary Figure 3. Delirium occurrence vs dementia prevalence by age group

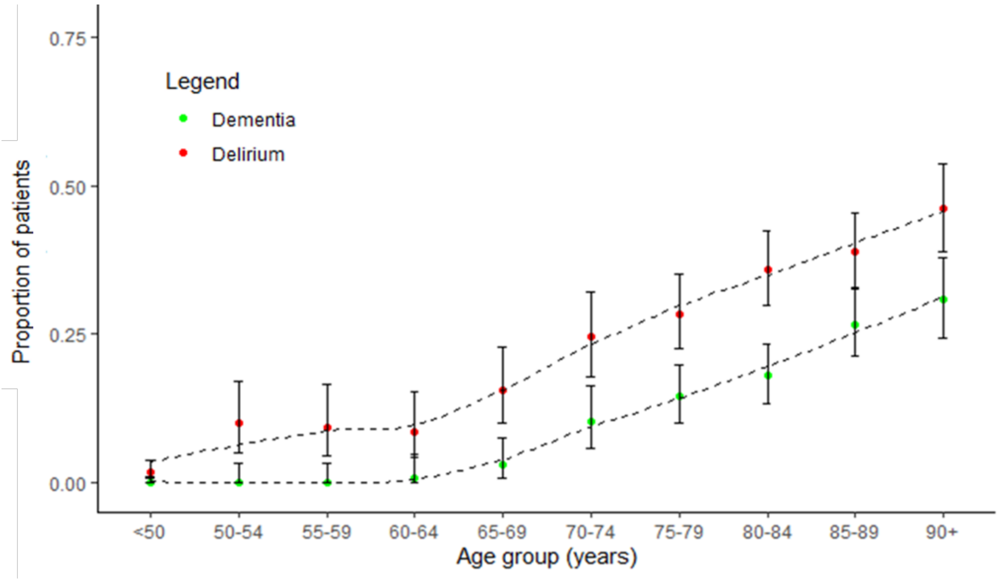

Supplementary Figure 4. Venn diagram displaying number with delirium, dementia and delirium superimposed on dementia stratified by age groups.

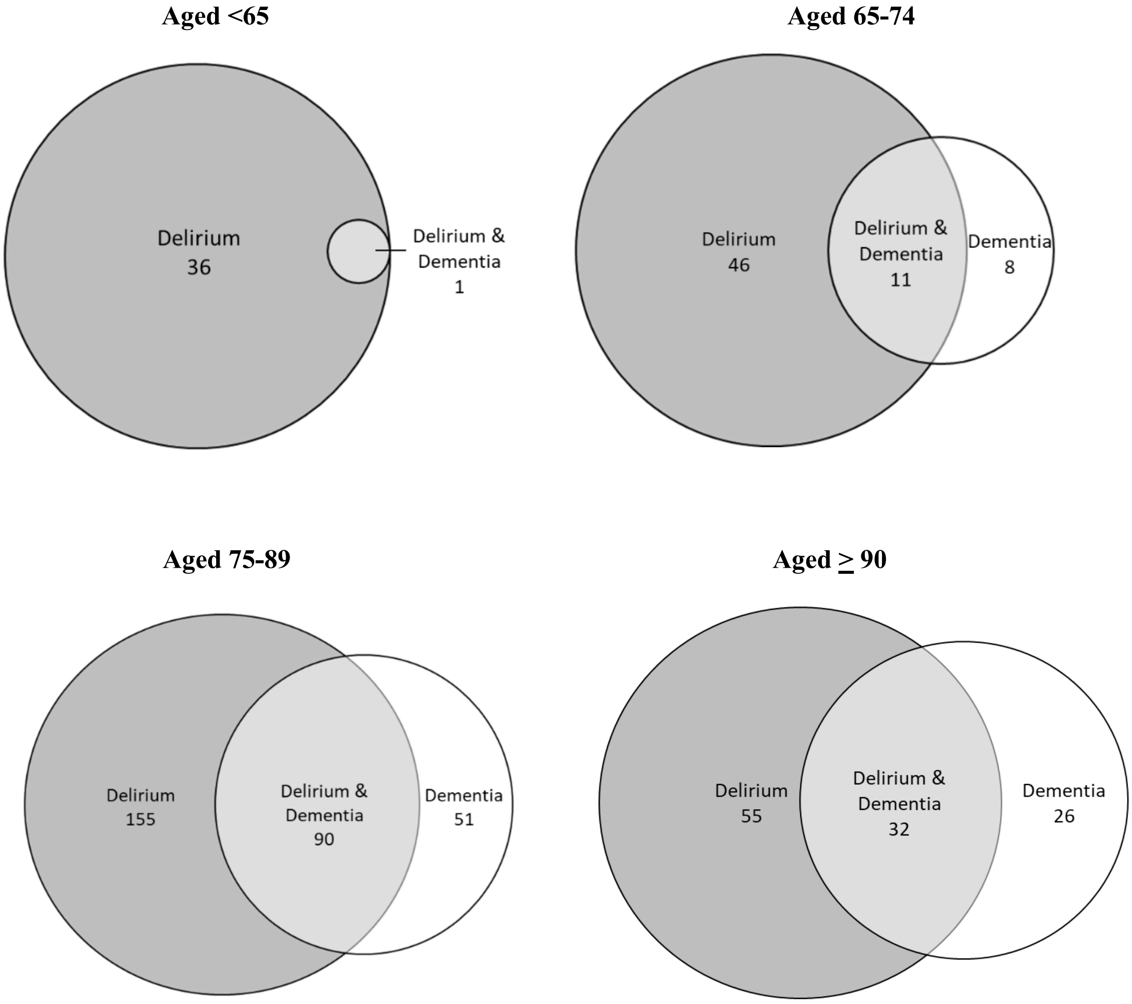

Supplementary Figure 5. Age-specific occurrence of delirium only, delirium superimposed on dementia and dementia only.

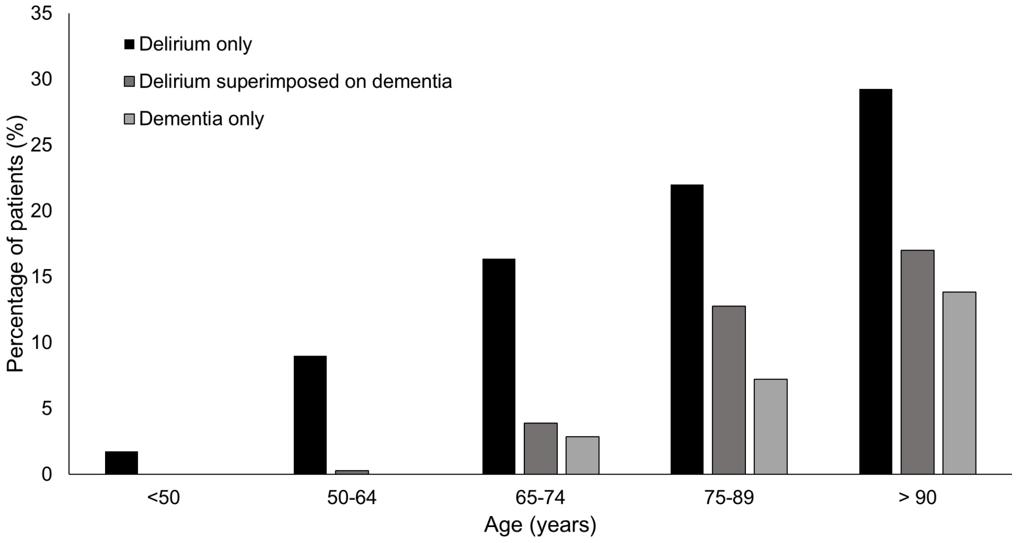

Supplementary Table 4. Characteristics of younger patients with delirium.

Number	Age Range	Prevalent Delirium	Incident Delirium	Brain Vulnerability	Diagnosis	SIRS
**1**	50-54	Yes	No	Acquired brain disease- Brain injury from accident	Post-ictal confusion state	0
**2**	60-64	Yes	No	Acquired brain disease- Previous stroke	Infection	2
**3**	55-59	Yes	No	Acquired brain disease- Recurrence of glioma	Status epilepticus; Progression of glioma	2
**4**	30-34	Yes	No	Alcohol	Alcohol withdrawal; Drug overdose (Keppra)	1
**5**	35-39	No	Yes	Alcohol	End stage alcoholic liver disease; Pleural effusion	2
**6**	55-59	No	Yes	Alcohol	Seizure; Intracranial haemorrhage; Psychosis	3
**7**	55-59	No	Yes	Alcohol	Alcoholic liver disease, Hepatic encephalopathy	1
**8**	50-54	Yes	No	Epilepsy	Seizure	1
**9**	60-64	Yes	No	Epilepsy	Infection; Heart failure; Carbon dioxide retention on background of chronic obstructive pulmonary disease	1
**10**	35-39	Yes	No	Learning disability/ Neurodevelopmental disorder	Infection	1
**11**	50-54	Yes	No	Learning disability/ Neurodevelopmental disorder	Infection; Constipation; Dehydration on background of renal failure	2
**12**	60-64	Yes	No	Learning disability/ Neurodevelopmental disorder	Infection	3
**13**	50-54	Yes	No	Mental health- Bipolar disorder	Infection	1
**14**	60-64	Yes	No	Mental health- Bipolar disorder	Infection; Acute Kidney Injury	2[a]
**15**	50-54	No	Yes	Mental health- Depression	Infection; Seizure	3
**16**	50-54	Yes	No	Mental health- Schizoaffective under Section 3	Dehydration	0[b]
**17**	45-49	Yes	No	Mental health- Stress	Functional disorder	1
**18**	55-59	Yes	No	Multiple Sclerosis	Infection	2
**19**	60-64	Yes	No	Multiple Sclerosis	Infection	2
**20**	55-59	Yes	No	Multiple Sclerosis	Infection	-[c]
**21**	50-54	Yes	No	Acquired brain disease- Previous craniotomy; Alcohol; Cerebral atrophy	Alcohol Withdrawal	3
**22**	60-64	Yes	No	Acquired brain disease- Glioma resection, Previous stroke; Epilepsy	Refractory epilepsy; Infection	4
**23**	60-64	Yes	No	Acquired brain disease- Previous intracranial haemorrhage; Mental health- depression,	No clear organic cause identified	1
**24**	60-64	Yes	No	Alcohol-related; Mental health- Schizoaffective, Epilepsy	Infection; Seizure	2
**25**	60-64	Yes	No	Alcohol-related; Mental health- Depression	Traumatic subarachnoid haemorrhage; Diabetic Ketoacidosis; Acute Kidney Injury	2
**26**	45-49	Yes	No	Epilepsy; Learning disability/ Neurodevelopmental disorder	Infection	1
**27**	55-59	Yes	Yes	Epilepsy; Learning disability/ Neurodevelopmental disorder	Infection; Seizure	2
**28**	60-64	Yes	No	Epilepsy; Learning disability/ Neurodevelopmental disorder	Hyperglycaemia	0

**29**	40-44	Yes	Yes	None identified	Palindromic rheumatism; Acute kidney injury	3
**30**	50-54	Yes	No	None identified	Hypertensive encephalopathy; Cerebral infarcts, Hyperglycaemia	1
**31**	50-54	Yes	No	None identified	Infection	4
**32**	50-54	Yes	No	None identified	Hypertensive encephalopathy	1
**33**	55-59	Yes	No	None identified	Hypoglycaemia	1
**34**	55-59	Yes	No	None identified	Cerebral metastasis	1
**35**	55-59	Yes	No	None identified	Electrolyte imbalance- hyponatraemia	1
**36**	55-59	Yes	No	None identified	Infection	2
**37**	50-54	Yes	No	None identified	For further outpatient neurology investigation	0

[a]Incomplete data: Temperature marked as afebrile.
[b]Incomplete data: Temperature marked as afebrile, respiratory rate value missing.
[c]Missing data.

Supplementary Table 5. Factors associated with prevalent delirium only in patients aged ≥65 years (n=259)

	All n=1043	Prevalent delirium n=259	No delirium n=784	OR	p-value	Adjusted OR[a]	Adjusted p-value
**Demographic factors**							
Age >75 years	752	208 (80.3)	544 (69.4)	**1.80 (1.28-2.53)**	**<0.001**		
Female	502	142 (54.8)	399 (50.9)	1.17 (0.88-1.55)	0.272		
**Medical history**							
Dementia	180	95 (36.7)	85(10.8)	**4.76 (3.40-6.68)**	**<0.001**	**4.23 (2.99-5.99)**	**<0.001**
Falls	442	163 (62.9)	279 (35.6)	**3.06 (2.29-4.10)**	**<0.001**	**2.73 (2.02-3.69)**	**<0.001**
TIA/stroke	189	59 (22.8)	130 (16.6)	**1.48 (1.05-2.09)**	**0.027**	1.32 (0.92-1.87)	0.128
Depression	153	49 (18.9)	104 (13.3)	**1.52 (1.05-2.21)**	**0.027**	**1.62 (1.10-2.37)**	**0.013**
Other psychiatric history	94	26 (10.0)	68 (8.7)	1.17 (0.73-1.88)	0.521	1.22 (0.75-1.98)	0.423
Visual impairment	145	61 (23.6)	84 (10.7)	**2.57 (1.78-3.70)**	**<0.001**	**2.18 (1.49-3.18)**	**<0.001**
Hearing impairment[b]	45	21/209 (10.0)	24/580 (4.1)	**2.59 (1.41-4.76)**	**0.002**	**2.06 (1.10-3.85)**	**0.024**
Medications >3	801	210 (81.1)	591 (75.4)	1.27 (0.88-1.83)	0.199	1.21 (0.84-1.75)	0.312
Medications >7	422	94 (36.3)	328 (41.8)	0.76 (0.56-1.01)	0.059	0.77 (0.57-1.04)	0.086
Previous dependency	333	135 (52.1)	198 (25.3)	**3.21 (2.40-4.30)**	**<0.001**	**2.88 (2.12-3.89)**	**<0.001**
Care Home	92	45 (17.4)	47 (6.0)	**3.29 (2.13-5.09)**	**<0.001**	**2.91 (1.86-4.53)**	**<0.001**
Charlson score				**1.04 (1.02-1.05)**	**<0.001**	**1.03 (1.02-1.05)**	**<0.001**
**HFRS**				**1.17 (1.14-1.20)**	**<0.001**	**1.16 (1.13-1.20)**	**<0.001**
Low Risk	536	67 (25.9)	469 (59.8)				
Moderate Risk	386	132 (51.0)	254 (32.4)	**3.64 (2.61-5.07)**	**<0.001**	**3.43 (2.45-4.80)**	**<0.001**
High Risk	84	56 (21.6)	28 (3.6)	**14.0 (8.32-23.6)**	**<0.001**	**12.5 (7.32-21.3)**	**<0.001**
**Clinical parameters[c]**							
Low cognitive score	298	147/182 (80.8)	151/531 (28.4)	**10.6 (6.99-16.0)**	**<0.001**	**10.9 (7.11-16.7)**	**<0.001**
Clinical dehydration	294	139 (53.7)	155 (19.8)	**4.73 (3.50-6.39)**	**<0.001**	**4.57 (3.37-6.20)**	**<0.001**
Respiratory rate >20	186	54/210 (25.7)	132/569 (23.2)	1.15 (0.80-1.65)	0.465	1.13 (0.78-1.63)	0.530
Abnormal temperature	261	79 (30.5)	182 (23.2)	**1.39 (1.02-1.90)**	**0.038**	1.31 (0.96-1.80)	0.093
Abnormal WCC	334	110 (42.5)	224 (28.6)	**1.82 (1.36-2.44)**	**<0.001**	**1.81 (1.35-2.43)**	**<0.001**
Na <135 mmol/L	246	78/226 (34.5)	168/631 (26.6)	**1.45 (1.05-2.01)**	**0.025**	**1.43 (1.03-1.99)**	**0.035**
CRP >6 mmol/L	558	176/220 (80.0)	382/537 (71.1)	**1.62 (1.11-2.37)**	**0.012**	**1.59 (1.08-2.34)**	**0.018**
Urea:Creatinine ratio >100:1	278	84/224 (37.5)	194/621 (31.2)	**1.32 (0.96-1.82)**	**0.088**	1.22 (0.88-1.69)	0.237
SIRS ≥ 2	341	103 (39.8)	238 (30.4)	**1.46 (1.09-1.96)**	**0.012**	**1.42 (1.06-1.91)**	**0.020**
PSPS ≥ 6 Braden <19	364	162/235 (68.9)	202/571 (35.4)	**4.05 (2.93-5.61)**	**<0.001**	**3.76 (2.71-5.23)**	**<0.001**
MUST >0	98	37/155 (23.9)	61/345 (17.7)	1.46 (0.92-2.32)	0.108	1.36 (0.85-2.17)	0.198
**Diagnosis**							
Infection	481	179 (69.1)	302 (38.5)	**3.57 (2.65-4.82)**	**<0.001**	**3.38 (2.50-4.57)**	**<0.001**
Cardiac	125	8 (3.1)	117 (14.9)	**0.18 (0.09-0.38)**	**<0.001**	**0.18 (0.09-0.37)**	**<0.001**
Stroke	44	13 (5.0)	31 (4.0)	1.28 (0.66-2.49)	0.461	1.19 (0.61-2.32)	0.618
Other	444	69 (26.6)	375 (47.8)	**0.40 (0.29-0.54)**	**<0.001**	**0.42 (0.30-0.57)**	**<0.001**
**During admission**							
Urinary incontinence	278	135 (52.1)	143 (18.2)	**4.89 (3.61-6.63)**	**<0.001**	**4.50 (3.30-6.13)**	**<0.001**
Faecal incontinence	114	59 (22.8)	55 (7.0)	**3.91 (2.62-5.83)**	**<0.001**	**3.55 (2.37-5.33)**	**<0.001**
Bedbound	104	51 (19.7)	53 (6.8)	**3.38 (2.24-5.12)**	**<0.001**	**3.28 (2.15-5.00)**	**<0.001**
Sleep disturbance	116	68 (26.3)	48 (6.1)	**5.47 (3.66-8.18)**	**<0.001**	**5.45 (3.62-8.19)**	**<0.001**
Constipation	121	41 (15.8)	80 (10.2)	**1.64 (1.09-2.46)**	**0.017**	**1.53 (1.01-2.30)**	**0.044**
Inpatient falls	38	19 (7.3)	19 (2.4)	**3.20 (1.67-6.15)**	**<0.001**	**3.28 (1.69-6.36)**	**<0.001**
Urinary catheter insertion	95	47 (18.1)	48 (6.1)	**3.40 (2.21-5.22)**	**<0.001**	**3.20 (2.07-4.95)**	**<0.001**
CT brain scanning	265	121 (46.7)	144 (18.4)	**3.88 (2.86-5.26)**	**<0.001**	**3.93 (2.89-5.35)**	**<0.001**

Numbers are n (%), unless specified otherwise.
Denominator for respective variables are shown where missing data exceed 5%.
Patients who had both prevalent and incident delirium (n=61) are not included in this analysis.
[a]Adjusted for age and sex.
[b]All missing cases from Cohort 1 or 2.
[c]Clinical parameters are defined as follow, where applicable: Low cognitive score: AMTS <9 or MMSE <24; Abnormal temperature: temperature >38°C or <36°C; abnormal WCC (white cell count): $<4\times10^9$ or $>12\times10^9$ cells/L

AMTS: Abbreviated Mental Test Score; CRP: C-reactive protein; HFRS: Hospital Frailty Risk Score; MMSE: Mini-Mental State Examination; MUST: Malnutrition Universal Screening Tool; Na: Sodium; PSPS: Pressure Score Prediction Score; SIRS: systemic inflammatory response syndrome; TIA: transient ischaemic attack.

Supplementary Table 6. Factors associated with incident delirium only in patients aged ≥65 years (n=69)

	All n=853	Incident delirium n=69	No delirium n=784	OR	p-value	Adjusted OR[a]	Adjusted p-value
**Demographic factors**							
**Age >75 years**	604	60 (87.0)	544 (69.4)	**2.94 (1.44-6.02)**	**0.003**		
**Female**	415	39 (56.5)	399 (50.9)	1.25 (0.76-2.06)	0.371		
**Medical history**							
**Dementia**	103	18 (26.1)	85 (10.8)	**2.90 (1.62-5.20)**	**<0.001**	**2.27 (1.24-4.16)**	**0.008**
**Falls**	318	39 (56.5)	279 (35.6)	**2.34 (1.42-3.86)**	**<0.001**	**1.86 (1.10-3.14)**	**0.020**
**TIA/stroke**	141	11 (15.9)	130 (16.6)	0.95 (0.49-1.86)	0.884	0.81 (0.41-1.60)	0.545
**Depression**	119	15 (21.7)	104 (13.3)	**1.81 (0.99-3.33)**	**0.055**	**2.05 (1.10-3.82)**	**0.024**
**Other psychiatric history**	75	7 (10.1)	68 (8.7)	1.18 (0.52-2.68)	0.689	1.24 (0.54-2.86)	0.613
**Visual impairment**	99	15 (21.7)	84 (10.7)	**2.32 (1.25-4.28)**	**0.007**	1.81 (0.96-3.41)	0.068
**Hearing impairment[b]**	28	4/41(9.8)	24/580 (4.1)	2.51 (0.83-7.60)	0.105	1.97 (0.63-6.17)	0.247
**Medications >3**	650	59 (85.5)	591 (75.4)	1.61 (0.80-3.21)	0.179	1.49 (0.74-3.00)	0.262
**Medications >7**	362	34 (49.3)	328 (41.8)	1.26 (0.77-2.06)	0.366	1.25 (0.76-2.06)	0.373
**Previous dependency**	231	33 (47.8)	198 (25.3)	**2.70 (1.64-4.45)**	**<0.001**	**2.19 (1.30-3.70)**	**0.003**
**Care Home**	55	8 (11.6)	47 (6.0)	2.05 (0.93-4.54)	0.076	1.59 (0.70-3.60)	0.264
**Charlson score**				**1.05 (1.03-1.07)**	**<0.001**	**1.04 (1.02-1.07)**	**<0.001**
**HFRS**				**1.15 (1.10-1.20)**	**<0.001**	**1.14 (1.09-1.19)**	**<0.001**
**Low Risk**	484	15/65 (23.1)	469/751(62.5)				
**Moderate Risk**	292	38/65 (58.5)	254/751 (33.8)	**4.68 (2.52-8.67)**	**<0.001**	**4.20 (2.23-7.89)**	**<0.001**
**High Risk**	40	12/65 (18.5)	28/751 (3.7)	**13.4 (5.73-31.3)**	**<0.001**	**10.8 (4.45-26.1)**	**<0.001**
**Clinical parameters[c]**							
**Low cognitive score**	179	28/50 (56.0)	151/531 (28.4)	**3.20 (1.78-5.77)**	**<0.001**	**2.64 (1.43-4.88)**	**0.002**
**Clinical dehydration**	185	30 (43.5)	155 (19.8)	**3.17 (1.90-5.28)**	**<0.001**	**2.99 (1.79-5.02)**	**<0.001**
**Respiratory rate >20**	145	13/40 (32.5)	132/569 (23.2)	1.59 (0.80-3.18)	0.185	1.63 (0.81-3.26)	0.168
**Abnormal temperature**	200	18 (26.1)	182 (23.2)	1.13 (0.64-1.99)	0.672	1.06 (0.60-1.87)	0.852
**Abnormal WCC**	245	21 (30.4)	224 (28.6)	1.11 (0.65-1.90)	0.703	1.09 (0.63-1.88)	0.753
**Na <135 mmol/L**	186	18/44 (40.9)	168/631 (26.6)	**1.91 (1.02-3.57)**	**0.043**	**1.89 (1.00-3.55)**	**0.049**
**CRP >6 mmol/L**	420	38/41 (92.7)	382/537 (71.1)	**5.14 (1.56-16.9)**	**0.007**	**5.09 (1.54-16.8)**	**0.007**
**Urea:Creatinine ratio >100:1**	214	20/43 (46.5)	194/621 (31.2)	**1.91 (1.03-3.57)**	**0.041**	1.81 (0.95-3.42)	0.070
**SIRS ≥ 2**	262	24 (34.8)	238 (30.4)	1.17 (0.70-1.98)	0.547	1.14 (0.67-1.93)	0.632
**PSPS ≥ 6 Braden <19**	239	37/49 (75.5)	202/571 (35.4)	**5.63 (2.87-11.0)**	**<0.001**	**5.11 (2.58-10.1)**	**<0.001**
**MUST >0**	75	14/41 (34.1)	61/345 (17.7)	**2.41 (1.20-4.87)**	**0.014**	2.03 (0.99-4.18)	0.053
**Diagnosis**							
**Infection**	341	39 (56.5)	302 (38.5)	**2.08 (1.26-3.41)**	**0.004**	**1.92 (1.16-3.17)**	**0.011**
**Cardiac**	127	10 (14.5)	117 (14.9)	0.97 (0.48-1.94)	0.923	0.93 (0.46-1.87)	0.828
**Stroke**	37	6 (8.7)	31 (4.0)	2.31 (0.93-5.75)	0.071	2.05 (0.81-5.16)	0.129
**Other**	392	17 (24.6)	375 (47.8)	**0.36 (0.20-0.63)**	**<0.001**	**0.38 (0.22-0.68)**	**<0.001**
**During admission**							
**Urinary incontinence**	172	29 (42.0)	143 (18.2)	**3.23 (1.94-5.39)**	**<0.001**	**2.83 (1.68-4.76)**	**<0.001**
**Faecal incontinence**	66	11 (15.9)	55 (7.0)	**2.50 (1.24-5.04)**	**0.010**	**2.14 (1.05-4.37)**	**0.036**
**Bedbound**	71	18 (26.1)	53 (6.8)	**4.85 (2.65-8.88)**	**<0.001**	**4.57 (2.46-8.47)**	**<0.001**
**Sleep disturbance**	84	36 (52.2)	48 (6.1)	**17.1 (9.79-29.9)**	**<0.001**	**16.3 (9.21-28.8)**	**<0.001**
**Constipation**	106	26 (37.7)	80 (10.2)	**5.38 (3.13-9.24)**	**<0.001**	**4.90 (2.83-8.49)**	**<0.001**
**Inpatient falls**	35	16 (23.2)	19 (2.4)	**12.1 (5.89-24.9)**	**<0.001**	**11.2 (5.38-23.4)**	**<0.001**
**Urinary catheter insertion**	68	20 (29.0)	48 (6.1)	**6.22 (3.43-11.3)**	**<0.001**	**5.52 (3.01-10.1)**	**<0.001**
**CT brain scanning**	178	34 (49.3)	144 (18.4)	**4.30 (2.59-7.12)**	**<0.001**	**4.07 (2.44-6.78)**	**<0.001**

Numbers are n (%), unless specified otherwise
Denominator for respective variables are shown where missing data exceed 5%.
Patients who had both prevalent and incident delirium (n=61) are not included in this analysis.
[a]Adjusted for age and sex.
[b] All missing cases from Cohort 1 or 2.
[c]Clinical parameters are defined as follow, where applicable: Low cognitive score: AMTS <9 or MMSE <24; Abnormal temperature: temperature >38°C or <36°C; abnormal WCC (white cell count): $<4\times10^9$ or $>12\times10^9$ cells/L

AMTS: Abbreviated Mental Test Score; CRP: C-reactive protein; HFRS: Hospital Frailty Risk Score; MMSE: Mini-Mental State Examination; MUST: Malnutrition Universal Screening Tool; Na: Sodium; PSPS: Pressure Score Prediction Score; SIRS: systemic inflammatory response syndrome; TIA: transient ischaemic attack.

Supplementary Table 7. Factors associated with delirium in patients aged >65 years excluding readmissions.

	All n=1146	Delirium n=378	No delirium n=768	OR	p-value	Adjusted OR[a]	Adjusted p-value
**Demographic factors**							
**Age >75 years**	850	320 (84.7)	530 (69.0)	**2.48 (1.80-3.41)**	**<0.001**		
**Female**	592	203 (53.7)	389 (50.7)	1.13 (0.88-1.45)	0.331		
**Medical history**							
**Dementia**	209	125 (33.1)	84 (10.9)	**4.02 (2.95-5.50)**	**<0.001**	**3.35 (2.43-4.61)**	**<0.001**
**Falls**	500	229 (60.6)	271 (35.3)	**2.81 (2.18-3.62)**	**<0.001**	**2.34 (1.80-3.05)**	**<0.001**
**TIA/stroke**	211	85 (22.5)	126 (16.4)	**1.47 (1.08-2.01)**	**0.014**	1.26 (0.92-1.73)	0.156
**Depression**	170	71 (18.8)	99 (12.9)	**1.56 (1.12-2.18)**	**0.009**	**1.67 (1.19-2.36)**	**0.003**
**Other psychiatric history**	98	36 (9.5)	62 (8.1)	1.19 (0.78-1.83)	0.424	1.30 (0.84-2.03)	0.241
**Visual impairment**	168	88 (23.3)	80 (10.4)	**2.61 (1.87-3.64)**	**<0.001**	**2.10 (1.49-2.96)**	**<0.001**
**Hearing impairment**	51	28/282 (9.9)	23/555 (4.1)	**2.55 (1.44-4.52)**	**0.001**	**1.92 (1.07-3.47)**	**0.030**
**Medications >3**	888	312 (82.5)	576 (75.0)	**1.41 (1.02-1.94)**	**0.039**	1.33 (0.95-1.85)	0.093
**Medications >7**	457	141 (37.3)	316 (41.1)	0.81 (0.63-1.04)	0.098	0.82 (0.63-1.07)	0.138
**Previous dependency**	379	190 (50.3)	189 (24.6)	**3.09 (2.38-4.00)**	**<0.001**	**2.61 (1.99-3.42)**	**<0.001**
**Care Home**	104	59 (15.6)	45 (5.9)	**2.96 (1.97-4.46)**	**<0.001**	**2.48 (1.63-3.77)**	**<0.001**
**Charlson score**				**1.04 (1.03-1.05)**	**<0.001**	**1.04 (1.02-1.05)**	**<0.001**
**HFRS**				**1.18 (1.15-1.21)**	**<0.001**	**1.16 (1.13-1.20)**	**<0.001**
**Low Risk**	553	93 (24.6)	460 (59.9)				
**Moderate Risk**	449	201 (53.2)	248 (32.3)	**4.01 (3.00-5.36)**	**<0.001**	**3.63 (2.70-4.88)**	**<0.001**
**High Risk**	101	74 (19.6)	27 (3.5)	**13.6 (8.27-22.2)**	**<0.001**	**11.1 (6.67-18.3)**	**<0.001**
**Clinical parameters**							
**Low cognitive score[c]**	357	209/274 (76.3)	148/517 (28.6)	**8.02 (5.72-11.2)**	**<0.001**	**7.70 (5.44-10.9)**	**<0.001**
**Clinical dehydration**	340	187 (49.5)	153 (19.9)	**3.95 (3.02-5.17)**	**<0.001**	**3.78 (2.87-4.97)**	**<0.001**
**Respiratory rate >20**	208	80/279 (28.7)	128/553 (23.1)	1.34 (0.96-1.85)	0.083	1.33 (0.95-1.85)	0.099
**Abnormal temperature**	287	110 (29.1)	177 (23.0)	**1.33 (1.00-1.75)**	**0.048**	**1.24 (0.94-1.66)**	**0.134**
**Abnormal WCC**	373	153 (40.5)	220 (28.6)	1.69 (1.31-2.19)	<0.001	1.68 (1.29-2.19)	<0.001
**Na <135 mmol/L**	270	104/303 (34.3)	166/615 (27.0)	**1.41 (1.05-1.90)**	**0.022**	**1.39 (1.03-1.88)**	**0.033**
**CRP >6 mmol/L**	608	236/292 (80.8)	372/522 (71.3)	**1.70 (1.20-2.41)**	**0.003**	**1.63 (1.15-2.33)**	**0.007**
**Urea:Creatinine ratio >100:1**	306	118/299 (39.5)	188/605 (31.1)	**1.45 (1.08-1.93)**	**0.012**	1.31 (0.97-1.77)	0.075
**SIRS > 2**	385	151 (39.9)	234	1.50 (1.16-1.95)	0.002	**1.47 (1.12-1.91)**	**0.005**
**PSPS > 6 or Braden <19**	418	224/321 (69.8)	194/557 (34.8)	**4.32 (3.22-5.81)**	**<0.001**	**3.93 (2.91-5.30)**	**<0.001**
**MUST >0**	119	58/227 (25.6)	61/337 (18.1)	**1.55 (1.03-2.33)**	**0.034**	1.39 (0.92-2.11)	0.122
**Diagnosis**							
**Infection**	540	249 (65.9)	291 (37.9)	**3.16 (2.45-4.09)**	**<0.001**	**2.94 (2.26-3.82)**	**<0.001**
**Cardiac**	141	28 (7.4)	113 (14.7)	**0.46 (0.30-0.72)**	**<0.001**	**0.44 (0.28-0.68)**	**<0.001**
**Stroke**	53	22 (5.8)	31 (4.0)	1.47 (0.84-2.57)	0.179	1.37 (0.77-2.43)	0.285
**Other**	473	101 (26.7)	372 (48.4)	**0.39 (0.30-0.51)**	**<0.001**	**0.42 (0.32-0.55)**	**<0.001**
**During admission**							
**Urinary incontinence**	330	194 (51.3)	136 (17.7)	**4.90 (3.72-6.44)**	**<0.001**	**4.33 (3.27-5.72)**	**<0.001**
**Faecal incontinence**	133	79 (20.9)	54 (7.0)	**3.49 (2.40-5.05)**	**<0.001**	**3.01 (2.06-4.40)**	**<0.001**
**Bedbound**	137	85 (22.5)	52 (6.8)	**3.99 (2.75-5.78)**	**<0.001**	**3.84 (2.63-5.62)**	**<0.001**
**Sleep disturbance**	173	125 (33.1)	48 (6.3)	**7.44 (5.18-10.7)**	**<0.001**	**7.10 (4.91-10.3)**	**<0.001**
**Constipation**	160	83 (22.0)	77 (10.0)	**2.51 (1.79-3.52)**	**<0.001**	**2.28 (1.61-3.22)**	**<0.001**
**Inpatient falls**	61	42 (11.1)	19 (2.5)	**4.94 (2.83-8.62)**	**<0.001**	**4.80 (2.72-8.46)**	**<0.001**
**Urinary catheter insertion**	127	81 (21.4)	46 (6.0)	**4.27 (2.90-6.29)**	**<0.001**	**3.88 (2.61-5.75)**	**<0.001**
**CT brain scanning**	330	187 (49.5)	143 (18.6)	**4.26 (3.25-5.59)**	**<0.001**	**4.35 (3.29-5.75)**	**<0.001**

Numbers are n (%), unless specified otherwise.
Denominator for respective variables are shown where missing data exceed 5%.
[a]Adjusted for age and sex.
[b]All missing cases from Cohort 1 or 2.

Low cognitive score: AMTS <9 or MMSE <24; Abnormal temperature: temperature >38°C or <36°C; abnormal WCC (white cell count): <4×10^9 or >12×10^9 cells/L
AMTS: Abbreviated Mental Test Score; CRP: C-reactive protein; HFRS: Hospital Frailty Risk Score; MMSE: Mini-Mental State Examination; MUST: Malnutrition Universal Screening Tool; Na: Sodium; PSPS: Pressure Score Prediction Score; SIRS: systemic inflammatory response syndrome; TIA: transient ischaemic attack.

Supplementary Table 8. Factors associated with delirium in patients aged >65 years stratified by comorbid dementia status

	Dementia (n=218)							No Dementia (n=955)						
	All	Delirium n=133	No delirium n=85	OR	p-value	Adjusted OR[a]	Adjusted p-value	All	Delirium n=256	No delirium n=699	OR	p-value	Adjusted OR[a]	Adjusted p-value
**Demographic factors**														
**Age >75 years**	197	121	76	1.19 (0.48-2.97)	0.703			674	206	468	**2.03 (1.44-2.88)**	**<0.001**		
**Female**	124	68	56	**0.54 (0.31-0.95)**	**0.033**			485	142	343	1.29 (0.97-1.72)	0.080		
**Medical history**														
**Falls**	152	98	54	1.61 (0.89-2.89)	0.113	1.51 (0.83-2.74)	0.178	365	140	225	**2.53 (1.89-3.39)**	**<0.001**	**2.17 (1.60-2.94)**	**<0.001**
**TIA/stroke**	55	33	22	0.95 (0.51-1.77)	0.859	0.88 (0.47-1.66)	0.694	162	54	108	**1.46 (1.01-2.10)**	**0.042**	1.27 (0.88-1.84)	0.207
**Depression**	54	34	20	1.12 (0.59-2.11)	0.734	1.29 (0.67-2.48)	0.447	125	41	84	1.39 (0.93-2.09)	0.107	**1.53 (1.01-2.31)**	**0.045**
**Other psychiatric history**	20	9	11	0.49 (0.19-1.23)	0.130	0.53 (0.21-1.34)	0.179	85	28	57	1.38 (0.85-2.22)	0.191	1.48 (0.91-2.42)	0.114
**Visual impairment**	50	38	12	**2.43 (1.19-4.99)**	**0.015**	**2.34 (1.14-4.83)**	**0.021**	125	53	72	**2.27 (1.54-3.35)**	**<0.001**	**1.83 (1.22-2.74)**	**0.003**
**Hearing impairment[b]**	14	11/105	3/59	2.18 (0.58-8.17)	0.246	2.22 (0.59-8.39)	0.241	39	18/189	21/521	**2.51 (1.30-4.82)**	**0.006**	1.93 (0.98-3.80)	0.056
**Medications >3**	171	111	60	1.85 (0.94-3.66)	0.077	1.95 (0.97-3.89)	0.060	743	212	531	1.36 (0.93-2.00)	0.113	1.26 (0.85-1.86)	0.247
**Medications >7**	75	41	34	0.62 (0.35-1.11)	0.107	0.61 (0.34-1.08)	0.091	402	108	294	0.96 (0.71-1.28)	0.758	0.97 (0.72-1.30)	0.826
**Previous dependency**	165	103	62	1.27 (0.68-2.39)	0.450	1.39 (0.73-2.64)	0.315	231	95	136	**2.43 (1.78-3.34)**	**<0.001**	**2.09 (1.51-2.89)**	**<0.001**
**Care Home**	70	45	25	1.23 (0.68-2.21)	0.495	1.37 (0.75-2.52)	0.304	40	18	22	**2.32 (1.22-4.40)**	**0.010**	1.88 (0.98-3.62)	0.060
**Charlson score**				1.01 (0.98-1.03)	0.562	1.01 (0.98-1.03)	0.645				**1.02 (1.01-1.04)**	**0.009**	**1.02 (1.00-1.04)**	**0.018**
**HFRS**				**1.10 (1.04-1.15)**	**<0.001**	**1.10 (1.05-1.15)**	**<0.001**				**1.18 (1.14-1.22)**	**<0.001**	**1.17 (1.13-1.21)**	**<0.001**
**Low Risk**	41	13	28					523	82	441				
**Moderate Risk**	106	69	37	**4.02 (1.86-8.67)**	**<0.001**	**4.11 (1.89-8.97)**	**<0.001**	352	135	217	**3.35 (2.43-4.60)**	**<0.001**	**3.08 (2.23-4.26)**	**<0.001**
**High Risk**	64	50	14	**7.69 (3.17-18.6)**	**<0.001**	**7.98 (3.25-19.5)**	**<0.001**	44	30	14	**11.5 (5.86-22.7)**	**<0.001**	**9.40 (4.70-18.8)**	**<0.001**
**Clinical parameters[c]**														
**Low cognitive score**	135	85/91	50/57	1.98 (0.63-6.23)	0.241	1.99 (0.62-6.38)	0.245	230	129/189	101/474	**7.94 (5.45-11.6)**	**<0.001**	**7.75 (5.27-11.4)**	**<0.001**
**Clinical dehydration**	92	73	19	**4.30 (2.32-7.95)**	**<0.001**	**4.41 (2.36-8.22)**	**<0.001**	258	122	136	**3.78 (2.77-5.15)**	**<0.001**	**3.67 (2.68-5.02)**	**<0.001**
**Respiratory rate >20**	44	31/104	13/58	1.47 (0.70-3.10)	0.312	1.48 (0.70-3.14)	0.303	169	50/186	119/511	1.21 (0.83-1.78)	0.328	1.22 (0.83-1.80)	0.318
**Abnormal temperature**	62	45	17	**1.94 (1.02-3.70)**	**0.044**	**1.95 (1.01-3.78)**	**0.048**	233	68	165	1.13 (0.82-1.57)	0.461	1.09 (0.78-1.52)	0.607
**Abnormal WCC**	79	54	25	1.58 (0.88-2.83)	0.126	1.51 (0.83-2.72)	0.175	300	101	199	**1.65 (1.22-2.23)**	**0.001**	**1.64 (1.21-2.23)**	**0.002**
**Na <135 mmol/L**	36	23/109	13/65	1.07 (0.50-2.29)	0.862	1.10 (0.51-2.38)	0.813	**237**	82/205	155/566	**1.77 (1.27-2.47)**	**<0.001**	**1.72 (1.22-2.41)**	**0.002**
**CRP >6 mmol/L**	112	80/105	32/57	**2.50 (1.26-4.98)**	**0.009**	**2.58 (1.27-5.24)**	**0.009**	515	165/198	350/480	**1.86 (1.22-2.84)**	**0.006**	**1.83 (1.19-2.82)**	**0.006**
**Urea:Creatinine ratio >100:1**	57	37/108	20/63	1.12 (0.58-2.17)	0.737	1.18 (0.60-2.30)	0.635	257	83/202	174/558	**1.54 (1.10-2.15)**	**0.011**	1.37 (0.97-1.93)	0.074
**SIRS > 2**	85	61	24	**2.02 (1.12-3.65)**	**0.020**	**1.97 (1.08-3.60)**	**0.027**	306	92	214	1.26 (0.93-1.71)	0.134	1.25 (0.92-1.71)	0.152
**PSPS > 6 Braden <19**	126	88/112	38/61	**2.22 (1.12-4.41)**	**0.023**	**2.31 (1.15-4.65)**	**0.019**	305	141/219	164/510	**3.81 (2.73-5.32)**	**<0.001**	**3.60 (2.57-5.04)**	**<0.001**
**MUST >0**	34	20/72	14/32	0.50 (0.21-1.18)	0.112	0.52 (0.21-1.25)	0.142	85	38/160	47/313	**1.76 (1.09-2.84)**	**0.020**	1.59 (0.98-2.60)	0.061
**Diagnosis**														
**Infection**	125	95	30	**4.58 (2.56-8.21)**	**<0.001**	**4.56 (2.49-8.36)**	**<0.001**	434	162	272	**2.71 (2.01-3.64)**	**<0.001**	**2.56 (1.90-3.46)**	**<0.001**
**Cardiac**	11	6	5	0.76 (0.22-2.56)	0.653	0.78 (0.23-2.68)	0.692	134	22	112	**0.49 (0.30-0.80)**	**0.004**	**0.47 (0.29-0.76)**	**0.002**
**Stroke**	8	2	6	0.20 (0.04-1.02)	0.053	0.24 (0.05-1.21)	0.084	45	20	25	**2.29 (1.25-4.19)**	**0.008**	**2.09 (1.13-3.88)**	**0.019**
**Other**	80	33	47	**0.27 (0.15-0.48)**	**<0.001**	**0.27 (0.15-0.48)**	**<0.001**	399	71	328	**0.43 (0.32-0.59)**	**<0.001**	**0.46 (0.33-0.63)**	**<0.001**
**During admission**														
**Urinary incontinence**	139	96	43	**2.61 (1.47-4.62)**	**0.001**	**2.65 (1.48-4.73)**	**0.001**	205	105	100	**4.14 (2.98-5.74)**	**<0.001**	**3.72 (2.67-5.19)**	**<0.001**
**Faecal incontinence**	59	41	18	1.68 (0.89-3.17)	0.112	1.72 (0.90-3.28)	0.101	78	41	37	**3.39 (2.12-5.43)**	**<0.001**	**3.00 (1.86-4.85)**	**<0.001**
**Bedbound**	49	36	13	**2.08 (1.03-4.20)**	**0.042**	**2.19 (1.07-4.48)**	**0.031**	90	50	40	**3.98 (2.55-6.21)**	**<0.001**	**3.86 (2.45-6.06)**	**<0.001**
**Sleep disturbance**	58	48	10	**4.29 (2.03-9.07)**	**<0.001**	**4.51 (2.11-9.66)**	**<0.001**	**120**	82	38	**8.22 (5.40-12.5)**	**<0.001**	**7.88 (5.15-12.1)**	**<0.001**
**Constipation**	47	33	14	1.69 (0.84-3.39)	0.139	1.69 (0.84-3.41)	0.143	**119**	53	66	**2.48 (1.67-3.68)**	**<0.001**	**2.24 (1.50-3.34)**	**<0.001**
**Inpatient falls**	20	17	3	**4.04 (1.15-14.2)**	**0.030**	**3.77 (1.06-13.4)**	**0.041**	**42**	26	16	**4.83 (2.54-9.16)**	**<0.001**	**4.68 (2.44-8.99)**	**<0.001**
**Urinary catheter insertion**	27	24	3	**6.07 (1.77-20.9)**	**0.004**	**5.53 (1.59-19.2)**	**0.007**	**104**	59	45	**4.33 (2.84-6.58)**	**<0.001**	**3.85 (2.51-5.90)**	**<0.001**
**CT brain scanning**	77	60	17	**3.29 (1.75-6.18)**	**<0.001**	**3.39 (1.77-6.48)**	**<0.001**	**258**	131	127	**4.70 (3.44-6.41)**	**<0.001**	**4.58 (3.34-6.29)**	**<0.001**

[a]Adjusted for age and sex.
[b]All missing cases from Cohort 1 or 2.

[c]Clinical parameters are defined as follow, where applicable: Low cognitive score: AMTS <9 or MMSE <24; Abnormal temperature: temperature >38°C or <36°C; abnormal WCC (white cell count): <4×109 or >12×109 cells/L

AMTS: Abbreviated Mental Test Score; CRP: C-reactive protein; HFRS: Hospital Frailty Risk Score; MMSE: Mini-Mental State Examination; MUST: Malnutrition Universal Screening Tool; Na: Sodium; PSPS: Pressure Score Prediction Score; SIRS: systemic inflammatory response syndrome; TIA: transient ischaemic attack.

Supplementary Table 9a. Outcomes in any delirium (patients aged ≥65 years).

	All n=1173	Delirium n=389	No delirium n=784	OR	p-value	Adjusted OR[a]	Adjusted p-value	Adjusted OR[b]	Adjusted p-value[b]
**LOS >7 days**	333	180 (46.3)	153 (19.5)	**3.55 (2.72-4.64)**	**<0.001**	**3.30 (2.52-4.33)**	**<0.001**	**2.48 (1.84-3.35)**	**<0.001**
**Death during admission**	100	60 (15.4)	40 (5.1)	**3.39 (2.23-5.17)**	**<0.001**	**2.97 (1.93-4.55)**	**<0.001**	**2.45 (1.52-3.94)**	**<0.001**

	All n=1073	Delirium n=329	No delirium n=744	OR	p-value	Adjusted OR[a]	Adjusted p-value	Adjusted OR[b]	Adjusted p-value[b]
**Patients who survived admission**									
**Increased care**	234	133 (40.4)	101 (13.6)	**4.29 (3.16-5.81)**	**<0.001**	**3.78 (2.77-5.16)**	**<0.001**	**2.41 (1.70-3.40)**	**<0.001**
**New placement**	42	24 (7.3)	18 (2.4)	**3.17 (1.70-5.93)**	**<0.001**	**2.47 (1.30-4.68)**	**0.006**	1.57 (0.77-3.19)	0.218

Numbers are n (%), unless specified otherwise.
[a]Adjusted for age and sex.
[b]Adjusted for age, sex, comorbidity burden, illness severity and frailty (HFRS).

LOS: Length of stay

Supplementary Table 9b. Sensitivity analysis of outcomes associated with delirium in patients aged >65 years, replacing frailty with pre-admission dependency and comorbid dementia.

	All	Delirium n=389	No delirium n=784	OR	p-value	Adjusted OR[a]	Adjusted p-value	Adjusted OR[b]	Adjusted p-value[b]
**All patients aged ≥65, n=1173**									
**LOS >7 days**	333	180 (46.3)	153 (19.5)	**3.55 (2.72-4.64)**	**<0.001**	**2.48 (1.84-3.35)**	**<0.001**	**3.33 (2.47-4.48)**	**<0.001**
**Death during admission**	100	60 (15.4)	40 (5.1)	**3.39 (2.23-5.17)**	**<0.001**	**2.45 (1.52-3.94)**	**<0.001**	**2.64 (1.65-4.20)**	**<0.001**
		**Delirium n=329**	**No delirium n=744**	**OR**	**p-value**	**Adjusted OR[a]**	**Adjusted p-value**	**Adjusted OR[b]**	**Adjusted p-value[b]**
**Patients who survived admission, n=1073**									
**Increased care**	234	133 (40.4)	101 (13.6)	**4.29 (3.16-5.81)**	**<0.001**	**2.41 (1.70-3.40)**	**<0.001**	**3.60 (2.57-5.05)**	**<0.001**
**New placement**	42	24 (7.3)	18 (2.4)	**3.17 (1.70-5.93)**	**<0.001**	1.57 (0.77-3.19)	0.218	1.51 (0.74-3.06)	0.255

Numbers are n (%), unless specified otherwise
[a]Adjusted for age, sex, comorbidity burden, illness severity and frailty (HFRS).
[b]Adjusted for age, sex, comorbidity burden, illness severity, pre-admission dependency and previous dementia.

LOS: Length of stay

Supplementary Table 10a. Outcomes in prevalent delirium only (patients aged >65 years, n=259)

	All n=1043	Prevalent Delirium n=259	No prevalent delirium n=784	OR	p-value	Adjusted OR[a]	Adjusted p-value	Adjusted OR[b]	Adjusted p-value[b]
**LOS >7 days**	243	90 (34.7)	153 (19.5)	**2.20 (1.61-3.00)**	**<0.001**	**2.08 (1.52-2.85)**	**<0.001**	**1.50 (1.05-2.13)**	**0.024**
**Death during admission**	75	35 (13.5)	40 (5.1)	**2.91 (1.80-4.69)**	**<0.001**	**2.57 (1.58-4.18)**	**<0.001**	**1.92 (1.12-3.31)**	**0.018**

	All n=968	Prevalent Delirium n=224	No prevalent delirium n=744	OR	p-value	Adjusted OR[a]	Adjusted p-value	Adjusted OR[b]	Adjusted p-value[b]
**Patients who survived admission**									
**Increased care**	181	80 (35.7)	101 (13.6)	**3.51 (2.49-4.95)**	**<0.001**	**3.18 (2.23-4.52)**	**<0.001**	**1.86 (1.26-2.76)**	**0.002**
**New placement**	31	13 (5.8)	18 (2.4)	**2.49 (1.20-5.16)**	**0.014**	1.99 (0.95-4.20)	0.069	1.25 (0.55-2.85)	0.588

Numbers are n (%), unless specified otherwise.
[a]Adjusted for age and sex.
[b]Adjusted for age, sex, comorbidity burden, illness severity and frailty (HFRS).

LOS: Length of stay

Supplementary Table 10b. Outcomes in incident delirium (patients aged >65 years, n=69)

	All n=853	Incident Delirium n=69	No incident delirium n=784	OR	p-value	Adjusted OR[a]	Adjusted p-value	Adjusted OR[b]	Adjusted p-value[b]
**All patients aged >65**									
**LOS >7 days**	206	53 (76.8)	153 (19.5)	**13.7 (7.60-24.6)**	**<0.001**	**12.7 (7.01-22.9)**	**<0.001**	**9.33 (4.97-17.5)**	**<0.001**
**Death during admission**	55	15 (21.7)	40 (5.1)	**5.17 (2.69-9.94)**	**<0.001**	**4.53 (2.32-8.81)**	**<0.001**	**3.92 (1.85-8.31)**	**<0.001**

	All n=798	Incident Delirium n=54	No incident delirium n=744	OR	p-value	Adjusted OR[a]	Adjusted p-value	Adjusted OR[b]	Adjusted p-value[b]
**Patients who survived admission**									
**Increased care**	131	30 (55.6)	101 (13.6)	**7.90 (4.44-14.1)**	**<0.001**	**6.85 (3.79-12.4)**	**<0.001**	**4.80 (2.50-9.25)**	**<0.001**
**New placement**	22	4 (7.4)	18 (2.4)	**3.21 (1.05-9.86)**	**0.041**	2.43 (0.77-7.64)	0.128	1.18 (0.31-4.42)	0.812

Numbers are n (%), unless specified otherwise.
[a]Adjusted for age and sex.
[b]Adjusted for age, sex, comorbidity burden, illness severity and frailty (HFRS).

LOS: Length of stay

Supplementary Table 11. Outcomes in delirium (patients aged >65 years) excluding patients with readmissions.

	All n=1146	Delirium n=378	No delirium n=768	OR	p-value	Adjusted OR[a]	Adjusted p-value	Adjusted OR[b]	Adjusted p-value[b]
**LOS >7 days**	327	176 (46.6)	151 (19.7)	**3.56 (2.72-4.66)**	**<0.001**	**3.30 (2.51-4.34)**	**<0.001**	**2.46 (1.82-3.34)**	**<0.001**
**Death during admission**	99	59 (15.6)	40 (5.2)	**3.37 (2.21-5.14)**	**<0.001**	**2.92 (1.90-4.50)**	**<0.001**	**2.43 (1.51-3.92)**	**<0.001**

	All n=1047	Delirium n=319	No delirium n=728	OR	p-value	Adjusted OR[a]	Adjusted p-value	Adjusted OR[b]	Adjusted p-value[b]
**Patients who survived admission**									
**Increased care**	229	129 (40.4)	100 (13.7)	**4.23 (3.11-5.75)**	**<0.001**	**3.70 (2.70-5.06)**	**<0.001**	**2.36 (1.67-3.36)**	**<0.001**
**New placement**	41	23 (7.2)	18 (2.5)	**3.06 (1.63-5.76)**	**<0.001**	**2.36 (1.24-4.49)**	**0.009**	1.46 (0.71-3.01)	0.300

Numbers are n (%), unless specified otherwise.
[a]Adjusted for age and sex.
[b]Adjusted for age, sex, comorbidity burden, illness severity and frailty (HFRS).

LOS: Length of stay

Supplementary Table 12. Outcomes in delirium (patients aged ≥65 years) stratified by comorbid dementia status.

DEMENTIA

	**All n=218**	**Delirium n=133**	**No Delirium n=85**	**OR**	**p-value**	**Adjusted OR[a]**	**Adjusted p-value**	**Adjusted OR[b]**	**Adjusted p-value[b]**
**LOS >7 days**	84	59 (44.4)	25 (29.4)	**1.91 (1.07-3.41)**	**0.028**	**1.91 (1.06-3.43)**	**0.030**	1.81 (0.95-3.46)	0.072
**Death during admission**	29	21 (15.8)	8 (9.4)	1.81 (0.76-4.28)	0.181	1.84 (0.76-4.47)	0.176	1.47 (0.58-3.75)	0.419
	**All n=189**	**Delirium n=112**	**No Delirium n=77**	**OR**	**p-value**	**Adjusted OR[a]**	**Adjusted p-value**	**Adjusted OR[b]**	**Adjusted p-value[b]**
**Patient who survived admission**									
**Increased care**	66	43 (38.4)	23 (29.9)	1.44 (0.77-2.67)	0.253	1.37 (0.72-2.57)	0.335	1.14 (0.56-2.32)	0.714
**New placement**	20	13 (11.6)	7 (9.1)	1.29 (0.49-3.41)	0.602	1.29 (0.49-3.44)	0.607	1.26 (0.43-3.68)	0.673

NO DEMENTIA

	**All n=955**	**Delirium n=256**	**No Delirium n=699**	**OR**	**p-value**	**Adjusted OR[a]**	**Adjusted p-value**	**Adjusted OR[b]**	**Adjusted p-value[b]**
**LOS >7 days**	**249**	121 (47.3)	128 (18.3)	**4.00 (2.93-5.46)**	**<0.001**	**3.72 (2.71-5.10)**	**<0.001**	**2.84 (2.00-4.02)**	**<0.001**
**Death during admission**	**71**	39 (15.2)	32 (4.6)	**3.75 (2.29-6.13)**	**<0.001**	**3.40 (2.06-5.61)**	**<0.001**	**3.02 (1.73-5.25)**	**<0.001**
	**All n=884**	**Delirium n=217**	**No Delirium n=667**	**OR**	**p-value**	**Adjusted OR[a]**	**Adjusted p-value**	**Adjusted OR[b]**	**Adjusted p-value[b]**
**Patient who survived admission**									
**Increased care**	**168**	90 (41.5)	78 (11.7)	**5.32 (3.71-7.61)**	**<0.001**	**4.76 (3.30-6.86)**	**<0.001**	**3.01 (2.01-4.51)**	**<0.001**
**New placement**	22	11 (5.1)	11 (1.0)	**3.19 (1.36-7.47)**	**0.007**	2.29 (0.95-5.50)	0.064	1.29 (0.48-3.47)	0.619

Numbers are n (%), unless specified otherwise
[a]Adjusted for age and sex.
[b]Adjusted for age, sex, comorbidity burden, illness severity and frailty (HFRS).

Supplementary Figure 6. Kaplan-Meier survival curve from admission and up to 10 years follow-up for all patients aged >65 years

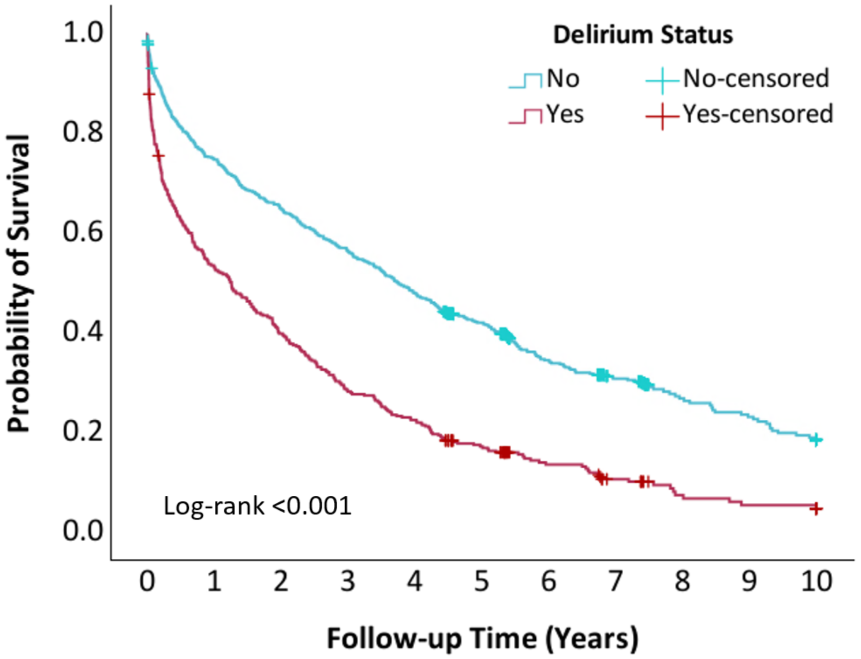

Number at risk (number censored)

Delirium	389(0)	206(2)	154(2)	109(2)	87(2)	58(11)	33(26)	21(31)	11(36)	8(36)	8(43)
No Delirium	784(0)	584(4)	506(4)	439(4)	374(4)	281(53)	165(128)	115(161)	58(208)	50(208)	40(248)

Supplementary Table 13a. Mortality risk across follow-up time.

	HR	p-value	Adjusted HR[a]	p-value
30 days	2.77 (1.97-3.90)	<0.001	2.03 (1.40-2.97)	<0.001
6 months	2.30 (1.83-2.90)	<0.001	1.72 (1.33-2.23)	<0.001
2 years	2.16 (1.81-2.57)	<0.001	1.71 (1.40-2.08)	<0.001
5 years	2.10 (1.82-2.43)	<0.001	1.60 (1.36-1.89)	<0.001
10 years	2.03 (1.77-2.33)	<0.001	1.52 (1.30-1.77)	<0.001

[a]Adjusted for age, sex, comorbidity burden, illness severity and frailty (HFRS).

Supplementary Table 13b. Sensitivity analysis of mortality risk across follow-up time, replacing frailty with pre-admission dependency and comorbid dementia.

	HR	p-value	Adjusted HR[a]	p-value	Adjusted HR[b]	p-value
30 days	2.77 (1.97-3.90)	<0.001	2.03 (1.40-2.97)	<0.001	2.04 (1.41-2.94)	<0.001
6 months	2.30 (1.83-2.90)	<0.001	1.72 (1.33-2.23)	<0.001	1.70 (1.32-2.19)	<0.001
2 years	2.16 (1.81-2.57)	<0.001	1.71 (1.40-2.08)	<0.001	1.64 (1.35-1.99)	<0.001
5 years	2.10 (1.82-2.43)	<0.001	1.60 (1.36-1.89)	<0.001	1.57(1.34-1.84)	<0.001
10 years	2.03 (1.77-2.33)	<0.001	1.52 (1.30-1.77)	<0.001	1.51 (1.30-1.76)	<0.001

[a]Adjusted for age, sex, comorbidity burden, illness severity and frailty (HFRS).
[b]Adjusted for age, sex, comorbidity burden, illness severity, previous dependency, previous dementia.

Supplementary Table 13c. Mortality risk of patients who survived preceding follow-up time.

	HR	p-value	Adjusted HR[a]	p-value	Adjusted HR[b]	p-value
30 days (n=1173)	2.77 (1.97-3.90)	<0.001	2.03 (1.40-2.97)	<0.001	2.04 (1.41-2.94)	<0.001
6 months (n=1040)	1.97 (1.44-2.69)	<0.001	1.51 (1.05-2.16)	0.027	1.46 (1.02-2.09)	0.037
2 years (n=882)	1.98 (1.51-2.60)	<0.001	1.67 (1.23-2.28)	0.001	1.55 (1.15-2.09)	0.004
5 years (n=666)	1.97 (1.53-2.55)	<0.001	1.43 (1.06-1.92)	0.019	1.46 (1.11-1.94)	0.008
10 years (n=403)	1.52 (0.98-2.35)	0.063	0.93 (0.55-1.58)	0.785	1.11 (0.67-1.83)	0.683

[a]Adjusted for age, sex, comorbidity burden, illness severity and frailty (HFRS).
[b]Adjusted for age, sex, comorbidity burden, illness severity, previous dependency, previous dementia.

Supplementary Table 14. Proportion of patients aged $\geq$ 65 years who were alive after 5 years and 10 years of follow-up from admission.

Survival status at 5-year follow-up

	All (n=980)	Delirium (n=327)	No Delirium (n=653)
**Alive at 5 years**	345	60 (18%)	285 (44%)
**Died at 5 years**	635	267 (82%)	368 (56%)

As mortality data was censored on 20th April 2023, this analysis excluded Cohort 6 patients (n=193) who were admitted between September-November 2018 and did not reach 5-year follow-up.

Survival status at 10-year follow-up

	All (n=309)	Delirium (n=96)	No Delirium (n=213)
**Alive at 10 years**	53	9 (9%)	44 (21%)
**Died at 10 years**	256	87 (91%)	169 (79%)

As mortality data was censored on 20th April 2023, this analysis excluded Cohort 3-6 patients (n=864) who were admitted from October 2015 and did not reach 10-year follow-up.

Supplementary Table 15a. Inpatient mortality risk in patients with delirium compared to those without delirium stratified by age group, dementia status, residence and frailty.

	OR	p-value	Adjusted OR[a]	p-value
Age group				
65-74 (n=281)	5.81 (2.06-16.4)	<0.001	4.38 (1.18-16.3)	0.028
75-89 (n=704)	2.77 (1.56-4.92)	<0.001	1.96 (1.02-3.75)	0.043
≥90 (n=188)	2.60 (1.18-5.77)	0.018	2.86 (1.14-7.16)	0.025
Known history of dementia				
Yes (n=218)	1.81 (0.76-4.28)	0.181	1.47 (0.58-3.75)	0.419
No (n=955)	3.75 (2.29-6.13)	<0.001	3.02 (1.73-5.25)	<0.001
Residence				
Care home (n=110)	1.95 (0.69-5.54)	0.208	1.49 (0.47-4.69)	0.499
Home (n=1029)	3.54 (2.18-5.73)	<0.001	2.85 (1.64-4.93)	<0.001
Frailty				
Low (n=564)	4.89 (2.30-10.4)	<0.001	2.75 (1.16-6.51)	0.022
Moderate (n=458)	2.30 (1.26-4.20)	0.007	2.03 (1.08-3.82)	0.028
High (n=108)	1.62 (0.43-6.16)	0.481	1.98 (0.48-8.22)	0.345

[a]Adjusted for age, sex, comorbidity burden, illness severity and frailty (HFRS).

Outcome for death during admission was binarised.

Supplementary Table 15b. Mortality risk over 10-year follow-up period in patients with delirium compared to those without delirium stratified by age group

	Age 65-74 (n=281)				Age 75-89 (n=704)				Age ≥ 90 (n=188)			
Follow-up Time	OR	p-value	Adjusted OR[a]	p-value	OR	p-value	Adjusted OR[a]	p-value	OR	p-value	Adjusted OR[a]	p-value
Death during admission	5.81 (2.06-16.4)	<0.001	4.38 (1.18-16.3)	0.028	2.77 (1.56-4.92)	<0.001	1.96 (1.02-3.75)	0.043	2.60 (1.18-5.77)	0.018	2.86 (1.14-7.16)	0.025
Follow-up Time	HR	p-value	Adjusted HR[a]	p-value	HR	p-value	Adjusted HR[a]	p-value	HR	p-value	Adjusted HR[a]	p-value
30 days	5.26 (2.08-13.3)	<0.001	3.50 (1.12-10.9)	0.031	2.18 (1.39-3.42)	<0.001	1.67 (1.01-2.75)	0.044	2.23 (1.16-4.29)	0.016	2.35 (1.17-4.73)	0.016
6 months	4.09 (2.24-7.44)	<0.001	2.68 (1.28-5.62)	0.009	1.91 (1.42-2.57)	<0.001	1.55 (1.12-2.16)	0.009	1.74 (1.09-2.78)	0.020	1.71 (1.01-2.88)	0.045
2 years	3.19 (2.02-5.02)	<0.001	1.84 (1.06-3.19)	0.031	1.81 (1.44-2.26)	<0.001	1.61 (1.25-2.08)	<0.001	1.78 (1.24-2.56)	0.002	1.83 (1.22-2.74)	0.004
5 years	2.86 (1.96-4.19)	<0.001	1.87 (1.18-2.96)	0.008	1.75 (1.46-2.10)	<0.001	1.50 (1.22-1.84)	<0.001	1.75 (1.29-2.39)	<0.001	1.78 (1.26-2.51)	0.001
10 years	2.56 (1.79-3.68)	<0.001	1.58 (1.03-2.42)	0.038	1.67 (1.41-1.98)	<0.001	1.42 (1.16-1.72)	<0.001	1.77 (1.30-2.39)	<0.001	1.83 (1.30-2.58)	<0.001

[a]Adjusted for age, sex, comorbidity burden, illness severity and frailty (HFRS).

Supplementary Table 15c. Mortality risk over 10-year follow-up period in patients with delirium compared to those without delirium stratified by dementia status

	Dementia Hx (n=218)				No Dementia Hx (n=955)			
Follow-up Time	OR	p-value	Adjusted OR[a]	p-value	OR	p-value	Adjusted OR[a]	p-value
Death during admission	1.81 (0.76-4.28)	0.181	1.47 (0.58-3.75)	0.419	3.75 (2.29-6.13)	<0.001	3.02 (1.73-5.25)	<0.001
Follow-up Time	HR	p-value	Adjusted HR[a]	p-value	HR	p-value	Adjusted HR[a]	p-value
30 days	1.66 (0.80-3.45)	0.178	1.42 (0.66-3.04)	0.372	3.04 (2.05-4.52)	<0.001	2.27 (1.48-3.48)	<0.001
6 months	1.61 (1.00-2.59)	0.052	1.51 (0.91-2.53)	0.114	2.29 (1.75-3.02)	<0.001	1.74 (1.29-2.36)	<0.001
2 years	1.75 (1.20-2.56)	0.004	1.86 (1.22-2.82)	0.004	2.09 (1.70-2.57)	<0.001	1.63 (1.29-2.05)	<0.001
5 years	1.51 (1.12-2.03)	0.006	1.56 (1.12-2.16)	0.008	2.02 (1.70-2.39)	<0.001	1.53 (1.26-1.86)	<0.001
10 years	1.45 (1.09-1.92)	0.011	1.46 (1.07-2.00)	0.018	1.95 (1.66-2.29)	<0.001	1.47 (1.23-1.77)	<0.001

[a]Adjusted for age, sex, comorbidity burden, illness severity and frailty (HFRS).

Supplementary Table 15d. Mortality risk over 10-year follow-up in patients with delirium compared to those without delirium stratified by residence status.

	Care Home Residence (n=110)				Home Residence (n=1029)			
**Follow-up Time**	**OR**	**p-value**	**Adjusted OR[a]**	**p-value**	**OR**	**p-value**	**Adjusted OR[a]**	**p-value**
**Death during admission**	1.95 (0.69-5.54)	0.208	1.49 (0.47-4.69)	0.499	**3.54 (2.18-5.73)**	**<0.001**	**2.85 (1.64-4.93)**	**<0.001**
**Follow-up Time**	**HR**	**p-value**	**Adjusted HR[a]**	**p-value**	**HR**	**p-value**	**Adjusted HR[a]**	**p-value**
**30 days**	1.99 (0.87-4.51)	0.101	1.36 (0.56-3.27)	0.498	**2.68 (1.80-4.00)**	**<0.001**	**2.21 (1.42-3.44)**	**<0.001**
**6 months**	1.79 (0.99-3.24)	0.056	1.38 (0.72-2.67)	0.337	**2.20 (1.70-2.86)**	**<0.001**	**1.76 (1.31-2.37)**	**<0.001**
**2 years**	1.61 (0.98-2.66)	0.062	1.39 (0.79-2.43)	0.253	**2.11 (1.74-2.56)**	**<0.001**	**1.70 (1.37-2.12)**	**<0.001**
**5 years**	**1.71 (1.13-2.61)**	**0.012**	1.63 (1.00-2.66)	0.051	**2.03 (1.73-2.38)**	**<0.001**	**1.56 (1.30-1.87)**	**<0.001**
**10 years**	**1.81 (1.20-2.71)**	**0.004**	**1.77 (1.09-2.86)**	**0.021**	**1.95 (1.67-2.26)**	**<0.001**	**1.47 (1.24-1.74)**	**<0.001**

[a]Adjusted for age, sex, comorbidity burden, illness severity and frailty (HFRS).

Of 1173 patients aged $\geq$65 years, 34 were transferred from other hospitals including community hospital, psychiatric hospital or specialist orthopaedic hospital where no acute general medical service was available. Therefore, these patients were excluded from the analysis because information on pre-admission residence was unavailable.

Note: Patients from care home did not survive beyond 8 years.

Supplementary 15e. Mortality risk over 10-year follow-up in patients with delirium compared to those without delirium stratified by frailty status.

	Low (n=564)				Moderate (n=458)				High (n=108)			
**Follow-up Time**	**OR**	**p-value**	**Adjusted OR[a]**	**p-value**	**OR**	**p-value**	**Adjusted OR[a]**	**p-value**	**OR**	**p-value**	**Adjusted OR[a]**	**p-value**
**Death during admission**	**4.89 (2.30-10.4)**	**<0.001**	**2.75 (1.16-6.51)**	**0.022**	**2.30 (1.26-4.20)**	**0.007**	**2.03 (1.08-3.82)**	**0.028**	1.62 (0.43-6.16)	0.481	1.98 (0.48-8.22)	0.345
**Follow-up Time**	**HR**	**p-value**	**Adjusted HR[a]**	**p-value**	**HR**	**p-value**	**Adjusted HR [a]**	**p-value**	**HR**	**p-value**	**Adjusted HR [a]**	**p-value**
**30 days**	**4.04 (2.22-7.33)**	**<0.001**	**2.44 (1.26-4.71)**	**0.008**	**2.07 (1.27-3.39)**	**0.004**	**1.84 (1.11-3.05)**	**0.018**	0.99 (0.36-2.76)	0.990	1.25 (0.43-3.61)	0.685
**6 months**	**2.38 (1.55-3.65)**	**<0.001**	1.51 (0.94-2.42)	0.089	**1.72 (1.22-2.41)**	**0.002**	**1.59 (1.12-2.26)**	**0.010**	1.73 (0.80-3.71)	0.162	2.09 (0.94-4.62)	0.069
**2 years**	**2.01 (1.44-2.79)**	**<0.001**	**1.45 (1.01-2.09)**	**0.045**	**1.78 (1.38-2.30)**	**<0.001**	**1.69 (1.30-2.21)**	**<0.001**	1.36 (0.78-2.38)	0.285	1.59 (0.87-2.89)	0.132
**5 years**	**2.14 (1.65-2.79)**	**<0.001**	**1.61 (1.21-2.15)**	**0.001**	**1.63 (1.32-2.01)**	**<0.001**	**1.52 (1.22-1.90)**	**<0.001**	1.11 (0.71-1.73)	0.662	1.37 (0.82-2.29)	0.228
**10 years**	**1.82 (1.41-2.34)**	**<0.001**	**1.38 (1.05-1.81)**	**0.022**	**1.65 (1.35-2.02)**	**<0.001**	**1.53 (1.23-1.89)**	**<0.001**	1.11 (0.71-1.73)	0.662	1.38 (0.83-2.31)	0.216

[a]Adjusted for age, sex, comorbidity burden, illness severity and frailty (HFRS).
